# Silver-Catalyzed Controlled Intermolecular Cross-Coupling of Silyl Enol Ethers: Scalable Access to 1,4-Diketones

**DOI:** 10.1021/acs.orglett.2c01477

**Published:** 2022-06-17

**Authors:** Li Xu, Xiaoyi Liu, Gregory R. Alvey, Andrey Shatskiy, Jian-Quan Liu, Markus D. Kärkäs, Xiang-Shan Wang

**Affiliations:** † School of Chemistry and Materials Science, Jiangsu Key Laboratory of Green Synthesis for Functional Materials, Jiangsu Normal University, Xuzhou, Jiangsu 221116, China; ‡ Department of Chemistry, KTH Royal Institute of Technology, SE-100 44 Stockholm, Sweden

## Abstract

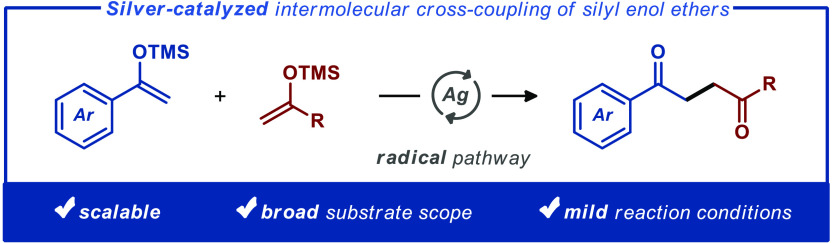

A protocol for silver-catalyzed controlled intermolecular cross-coupling of silyl enolates is disclosed. The protocol displays good functional group tolerance and allows efficient preparation of a series of synthetically useful 1,4-diketones. Preliminary mechanistic investigations suggest that the reaction proceeds through a one-electron process involving free radical species in which PhBr acts as the oxidant.

Metal-catalyzed cross-couplings and related reactions involving carbon-based nucleophilic reagents are widely employed in academia and industry.^1^ Cross-coupling reactions remain one of the most vibrant areas of chemistry and enable straightforward preparation of, for example, pharmaceuticals, agrochemicals, and materials. However, cross-coupling reactions that utilize coupling partners of a similar chemical nature are frequently accompanied by undesired homocoupling reactions. Therefore, further advancements in cross-coupling technologies to involve alternative precursors and more versatile reaction systems are required.

Enolates make up a multifaceted class of building blocks in organic synthesis and are widely applied in cross-coupling manifolds. Significant progress in cross-coupling reactions of enolates with electrophiles or free radical species has been detailed.^2,3^ These reactions allow various synthetic manipulations, enabling access to α-functionalized carbonyl compounds, which function as critical synthetic intermediates in the preparation of various natural products and pharmacophores.^4^ Oxidative cross-coupling of carbon-centered nucleophiles constitutes an excellent approach for forging new carbon–carbon bonds, providing a strategic alternative to conventional nucleophile–electrophile coupling reactions. However, selective intermolecular cross-coupling of two different enolate equivalents encompasses a significant challenge due to the similarities in the steric and electronic properties of the two coupling partners.^5^ To the best of our knowledge, only a handful of reports detailing the cross-coupling of two different enolates or enolate equivalents have been disclosed.^6^ For example, in 1975, Saegusa and co-workers reported the pioneering work on intermolecular cross-coupling of two distinct enolates to furnish unsymmetrical 1,4-diketone scaffolds.^7^ In this strategy, suppressing the homocoupling reactions was achieved by utilizing a large excess of one of the coupling partners, thus significantly reducing the atom economy of the developed process. Later, Ruzziconi^8^ and Ohshiro^9^ also achieved oxidative cross-coupling of two different trimethylsilyl enol ethers that is promoted by ceric and oxovanadium oxidants, respectively. Thereafter, Schmittel and co-workers established an unprecedented method for oxidative intramolecular cyclization of silyl bis-enol ethers.^10^ Thomson and Wirth independently made use of an intramolecular traceless silicon tether to connect two different enolates, rendering the reaction intramolecular and neatly avoiding selectivity-related issues (Figure 1).^11,12^ However, these protocols require elaborate substrate feedstocks that rely on multistep preparation. Furthermore, Hirao and Amaya reported the vanadium-induced intermolecular cross-coupling of two different enolates, capitalizing on the differences in reactivity between silyl- and boron-based enolate equivalents (Figure 1).^13^ Recently, Szpilman and co-workers disclosed a procedure for oxidative cross-coupling of two different enolate equivalents using a hypervalent iodine(III) compound (Koser’s reagent).^14^ However, this reaction requires extremely low temperatures and sequential addition of the coupling partners, decreasing the practicality of the disclosed protocol (Figure 1). Herein, we alleviate the drawbacks of the previously described cross-coupling manifolds of silyl enol ethers with the use of silver catalysis,^15,16^ providing a convenient method for the chemoselective preparation of a broad range of 1,4-dicarbonyl scaffolds under mild reaction conditions and using near-stoichiometric amounts of the two coupling partners (Figure 1).

**Figure 1:**
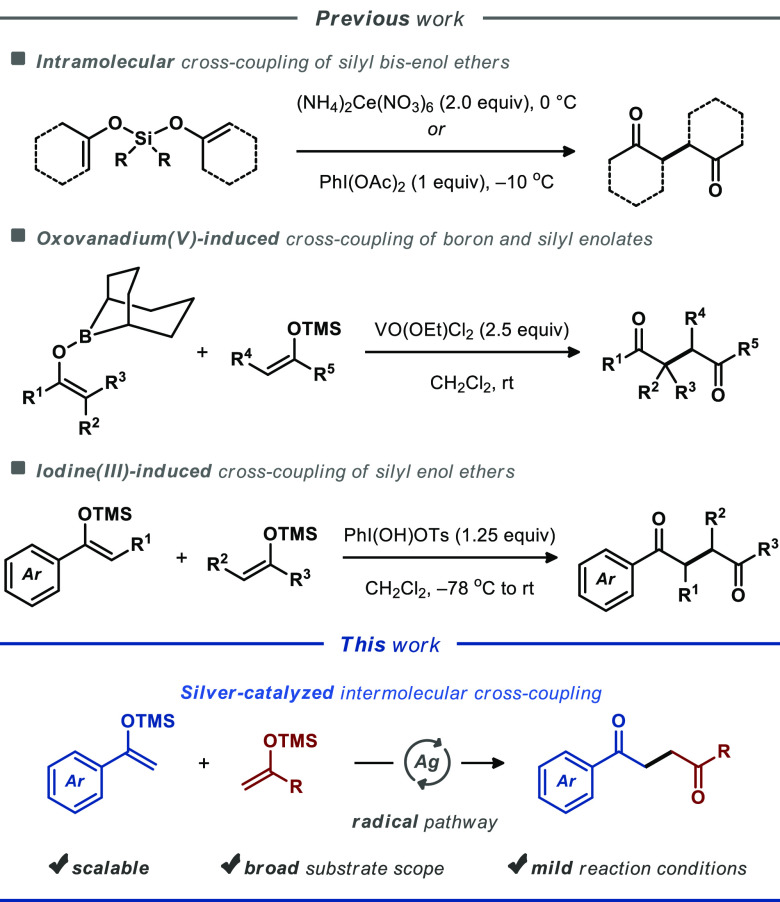
Oxidative cross-coupling strategies for the synthesis of 1,4-diketones.

At the beginning of our investigations, silyl enol ethers **1a** and **2a** were selected as model substrates to optimize the oxidative cross-coupling reaction (Table 1). To our delight, conducting the reaction in DMSO at 100 °C under air in the presence of AgF (30 mol %) and bromobenzene (2 equiv) exclusively afforded the desired 1,4-diketone **3a** in 9% isolated yield after 6 h, accompanied by the generation of homocoupling product **3s** in <5% yield (Table 1, entry 1). Performing the reaction under argon afforded the desired 1,4-diketone **3a** in 21% yield and homocoupling product **3s** in 11% yield (Table 1, entry 2). Encouraged by this result, we surveyed other silver-based precursors, including Ag_2_CO_3_, AgOTf, AgBF_4_, and AgOAc, and found that AgF displays the best reactivity while the others proved to be less efficient (Table 1, entries 3–6, respectively). Other metal-based catalysts, including InCl_3_, Cu(OTf)_2_, CuI, and Pd(OAc)_2_, provided a complex mixture of products and only trace amounts of product **3a** (Table 1, entries 7–10, respectively). Subsequently, switching the solvent to MeCN greatly increased the yield of cross-coupling product **3a** to 42% (Table 1, entry 11). In contrast, the use of aprotic or polar solvents, such as DMF, toluene, and DCE, had a negative effect on the reaction (Table 1, entries 12–14, respectively), while employing the protic solvent EtOH completely inhibited the reaction (Table 1, entry 15). Decreasing the reaction temperature from 100 °C to room temperature significantly improved the yield of the desired product **3a** to 72% (Table 1, entries 16–19), while decreasing the catalyst loading from 30 to 20 mol % had an insignificant influence on the reaction outcome (Table 1, entry 20). A control experiment in the absence of bromobenzene provided only trace amounts of the cross-coupling product (Table 1, entry 22), thus indicating that bromobenzene plays a critical role in the reaction.^17,18^

**Table 1:**
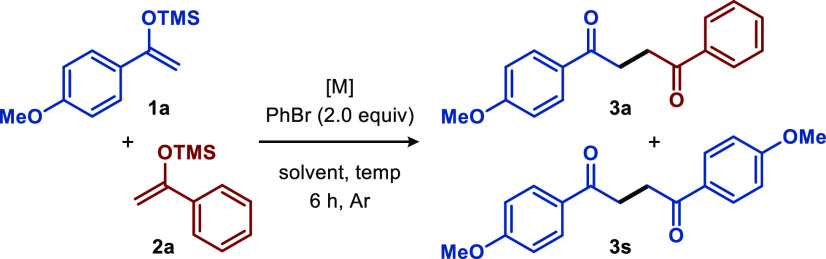
Optimization of the Reaction Conditions^a^

				yield (%)^b^	
entry	[M]	solvent	temp (°C)	**3a**	**3s**
1^c^	AgF	DMSO	100	9	<5
2	AgF	DMSO	100	21	11
3	Ag_2_CO_3_	DMSO	100	13	8
4	AgOTf	DMSO	100	0	0
5	AgBF_4_	DMSO	100	0	0
6	AgOAc	DMSO	100	15	9
7	InCl_3_	DMSO	100	0	0
8	Cu(OTf)_2_	DMSO	100	0	0
9	CuI	DMSO	100	0	0
10	Pd(OAc)_2_	DMSO	100	0	0
11	AgF	MeCN	100	42	29
12	AgF	DMF	100	28	21
13	AgF	toluene	100	35	26
14	AgF	DCE	100	27	13
15	AgF	EtOH	100	0	0
16	AgF	MeCN	80	47	31
17	AgF	MeCN	60	53	27
18	AgF	MeCN	40	64	25
19	AgF	MeCN	rt	72	19
20^d^	AgF	MeCN	rt	69	23
21^e^	AgF	MeCN	rt	43	28
22^f^	AgF	MeCN	rt	0	0
23	AgBr	MeCN	rt	23	12

a Reactions were carried out with **1a** (0.65 mmol), **2a** (0.5 mmol), catalyst (30 mol %), and PhBr (1.0 mmol) in solvent (2.0 mL) under argon for 6 h.

b Isolated yields of **3a** after purification by column chromatography.

c Reactions carried out under air.

d With 20 mol % catalyst.

e With 10 mol % catalyst.

f Reaction carried out in the absence of PhBr.

With the optimized reaction conditions in hand, we explored the generality of the established protocol (Scheme 1). An array of diversely functionalized silyl enol ethers **1** engaged in the desired cross-coupling reaction with silyl enol ethers **2** to afford the corresponding products **3** in good to excellent yields (Scheme 1). For example, aryl-based silyl enol ether motifs bearing electron-donating or electron-withdrawing moieties were tolerated in the cross-coupling reaction with **1a** to produce the corresponding products **3b–3h** in good yields. Gratifyingly, the utilization of heteroaryl silyl ethers, including 2-furyl and 2-thienyl, afforded the corresponding adducts **3i** and **3j**, respectively, illustrating the compatibility of the developed protocol. Subjecting alkyl-based silyl enol ethers to aryl-based silyl enol ethers generated the desired products **3q** and **3r** in good to excellent yields. Furthermore, the homocoupling reaction with aryl-based silyl enol ethers proceeds efficiently and yields the target compounds in ≤93% yield (Scheme 1). Finally, the structure of products **3** was supported through X-ray analysis of product **3ab** [CCDC 2143818 (see Scheme 1)]. To further explore the synthetic utility of the developed protocol, the applicability of the silver-catalyzed method was highlighted through a gram-scale reaction of **1a** and **2a** (Scheme 1). The reaction was performed on a 5 mmol scale and proceeded smoothly to give product **3a** (0.86 g, 64%). The highly functionalized 1,4-diketones developed by this protocol provide opportunities for a range of further synthetic manipulations, especially for heterocycle synthesis. For instance, synthetic conversion of diketone **3a** into functionalized pyrrole **4** and furan **5** was achieved in high yields by subjecting the diketone to ammonium acetate and triflic acid, respectively.^19^

**Scheme 1:**
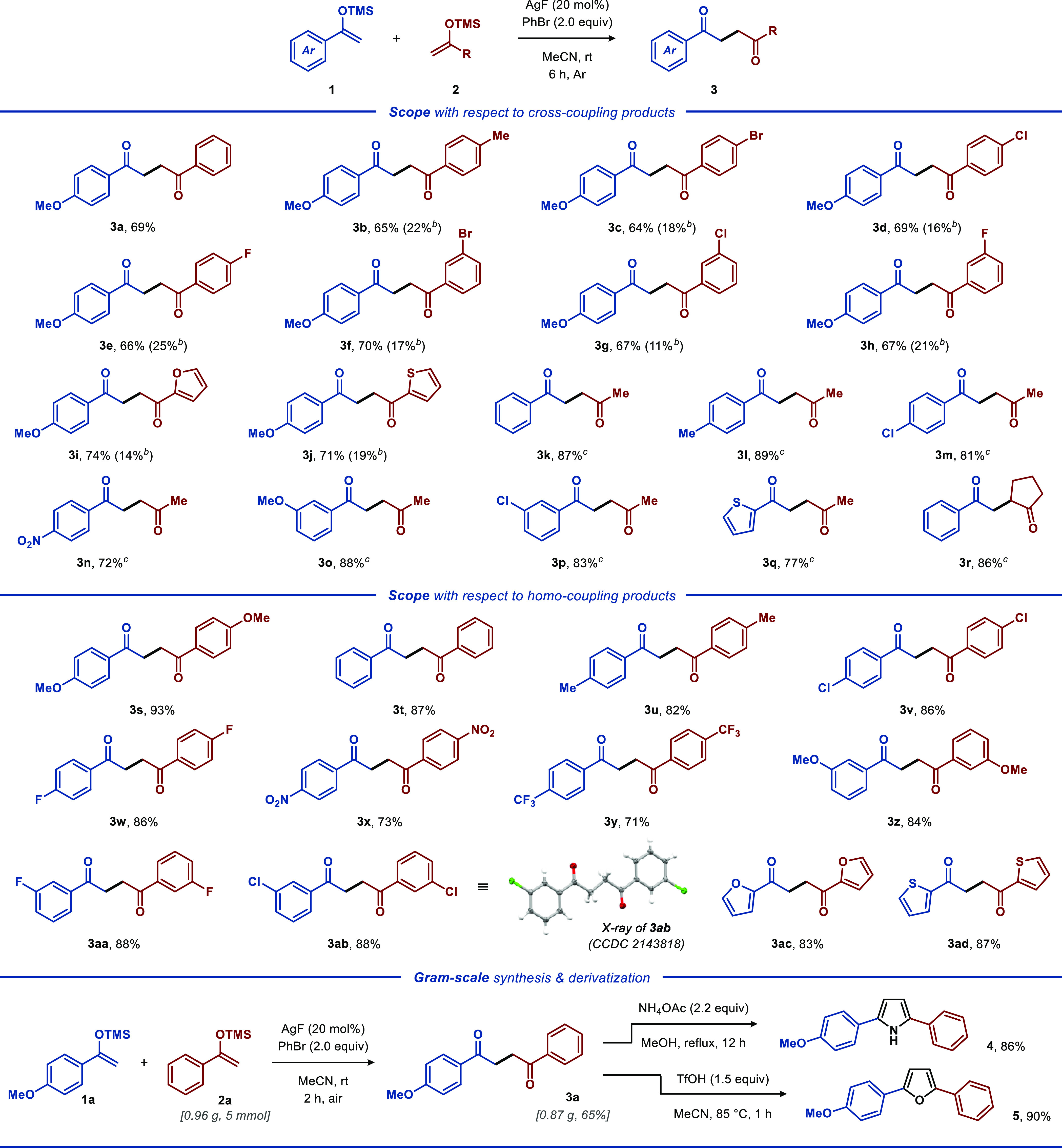
Silver-Catalyzed Synthesis of 1,4-Diketones All reactions were carried out with **1a** (0.65 mmol), **2a** (0.5 mmol), and AgF (20 mol %) in CH_3_CN (2.0 mL) at room temperature under air for 6 h. Yields are of isolated products after purification by column chromatography. Yields are of isolated homocoupling products of **1**. Reactions carried out at room temperature for 24 h.

Additional experiments were performed to gain insight into the mechanism of the developed transformation (Scheme 2). The silver-catalyzed homocoupling reaction of silyl enol ethers **1a** was completely suppressed by the addition of radical inhibitor TEMPO, indicating that the reaction proceeds through a free radical pathway.^20^ Additionally, we isolated TEMPO-based adduct **6** in 50% yield. To gain additional support for the proposed mechanism, ESI-MS experiments were performed, allowing detection of the phenyltrimethylsilane (PhTMS) byproduct (see Scheme 2 and Figure S1).^21^ Furthermore, when the reaction was conducted under the optimized conditions using AgBr as the silver precursor (Table 1, entry 23), only a small amount of the coupling product was produced, highlighting the essential role of fluoride for effective catalytic turnover. Also, we surmise that the developed reaction likely requires a nucleophilic counterion to mediate the desilylation of one of the silyl enol ethers upon conversion of Ag(II) intermediate **A** into AgF. Accordingly, while carbonate and acetate have sufficient nucleophilic character, OTf and BF_4_ cannot facilitate such a process (cf. Table 1, entries 3–6). Finally, considering the possible involvement of Ag(0) in the reaction, such processes would likely (although not necessarily) produce a silver mirror, as has been observed in our previous reports featuring catalytic amounts of Ag(I) catalysts.^22^ However, the formation of a silver mirror was not observed for the developed silyl enol ether cross-coupling reaction.

**Scheme 2:**
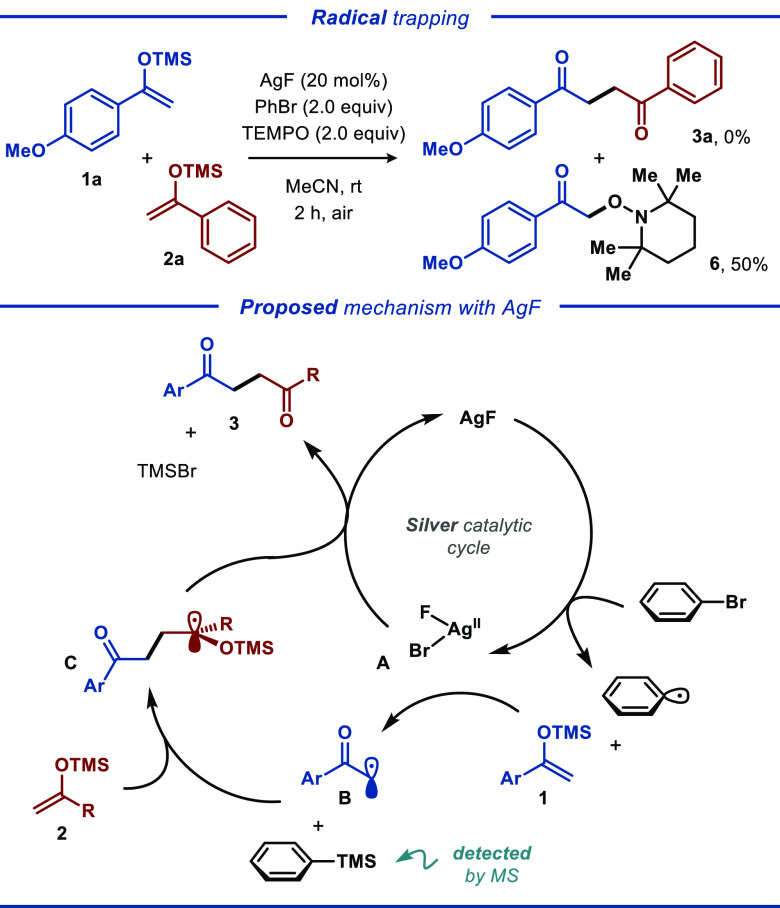
Radical Trapping Experiments and Proposed Mechanism

On the basis of the experimental results and literature precedents,^18,23^ a plausible mechanism for the cross-coupling reaction between **1** and **2** was proposed (Scheme 2). Initially, AgF abstracts a bromine atom from PhBr to generate Ag(II) intermediate **A** and a phenyl radical. Then, the transiently formed phenyl radical reacts with silyl enol ether **1** to furnish α-carbonyl radical **B** along with PhTMS. Finally, α-carbonyl radical **B** undergoes radical addition to silyl enol ether **2** to form carbon-centered radical **C**, which undergoes one-electron oxidation by Ag(II) species **A** to form cross-coupling product **3** along with regeneration of Ag(I), thereby closing the catalytic cycle. Preliminary mechanistic studies support the events mentioned above; however, further studies are required to elucidate the mechanism of this intriguing reaction. It is also possible that a radical–radical coupling mechanism could be operating under the disclosed reaction conditions, competing with the proposed mechanism. However, given the observed high selectivity, such a mechanism is not likely to play the key role.

In conclusion, we have developed a silver-catalyzed procedure for controlled intermolecular cross-coupling of silyl enol ethers. The protocol exhibits good functional group tolerance, allowing access to a range of synthetically valuable 1,4-diketones. A plausible free radical-based pathway is proposed in which PhBr acts as the oxidant. The disclosed method presents a versatile framework for oxidative carbon–carbon bond formation from unpretentious starting materials. It is anticipated that this reaction manifold will stimulate several new synthetic strategies. Further investigations of the reaction are ongoing in our laboratories.

## Supporting Information Available

Experimental procedures and characterization data for all of the compounds (PDF)
