# Supplementary material for: Silver-Catalyzed Controlled Intermolecular Cross-Coupling of Silyl Enol Ethers: Scalable Access to 1,4-Diketones
Source: Org Lett. 2022 Jun 17;24(25):4513–8. doi: 10.1021/acs.orglett.2c01477 (PMC9536665; doi:10.1021/acs.orglett.2c01477)
Supplement: Supplementary file 1 — ol2c01477_si_001.pdf [file ol2c01477_si_001.pdf]

*Supporting Information*

# Silver-Catalyzed Controlled Intermolecular Cross-Coupling of Silyl Enol Ethers: Scalable Access to 1,4-Diketones

Li Xu,[†] Xiaoyi Liu,[†] Gregory R. Alvey,[‡] Andrey Shatskiy,[‡] Jian-Quan Liu,[†,‡,]* Markus D. Kärkäs,[‡,]* Xiang-Shan Wang[†,]*

[†] School of Chemistry and Materials Science, Jiangsu Normal University, Xuzhou, Jiangsu 221116, China

[‡] Department of Chemistry, KTH Royal Institute of Technology, SE-100 44 Stockholm, Sweden

* E-mail: liujq316@jsnu.edu.cn (J.-Q.L.); karkas@kth.se (M.D.K.); xswang@jsnu.edu.cn (X.-S.W)

# Table of Contents

**I. General information** ........ **S1**

**II. Crystallography** ........ **S1**

**III. Synthesis of compounds 1** ........ **S3**

General procedure for the preparation of aryl silyl enol ethers ........ S3

**IV. Synthesis and analytical data of compounds 3** ........ **S3**

1-(4-Methoxyphenyl)-4-phenylbutane-1,4-dione (**3a**) ........ S3

Gram-scale synthesis of 1-(4-methoxyphenyl)-4-phenylbutane-1,4-dione (**3a**) ........ S3

1-(4-Methoxyphenyl)-4-(*p*-tolyl)butane-1,4-dione (**3b**) ........ S4

1-(4-Bromophenyl)-4-(4-methoxyphenyl)butane-1,4-dione (**3c**) ........ S5

1-(4-Chlorophenyl)-4-(4-methoxyphenyl)butane-1,4-dione (**3d**) ........ S5

1-(4-Fluorophenyl)-4-(4-methoxyphenyl)butane-1,4-dione (**3e**) ........ S6

1-(3-Bromophenyl)-4-(4-methoxyphenyl)butane-1,4-dione (**3f**) ........ S6

1-(3-Chlorophenyl)-4-(4-methoxyphenyl)butane-1,4-dione (**3g**) ........ S7

1-(3-Fluorophenyl)-4-(4-methoxyphenyl)butane-1,4-dione (**3h**) ........ S8

1-(Furan-2-yl)-4-(4-methoxyphenyl)butane-1,4-dione (**3i**) ........ S8

1-(4-Methoxyphenyl)-4-(thiophen-2-yl)butane-1,4-dione (**3j**) ........ S9

1-Phenylpentane-1,4-dione (**3k**) ........ S9

1-(*p*-Tolyl)pentane-1,4-dione (**3l**) ........ S10

1-(4-Chlorophenyl)pentane-1,4-dione (**3m**) ........ S10

1-(4-Nitrophenyl)pentane-1,4-dione (**3n**) ........ S11

1-(3-Methoxyphenyl)pentane-1,4-dione (**3o**) ........ S12

1-(3-Chlorophenyl)pentane-1,4-dione (**3p**) ........ S12

1-(Thiophen-2-yl)pentane-1,4-dione (**3q**) ........ S13

2-(2-Oxo-2-phenylethyl)cyclopentan-1-one (**3r**) ........ S13

1,4-Bis(4-methoxyphenyl)butane-1,4-dione (**3s**) ........ S14

1,4-Diphenylbutane-1,4-dione (**3t**) ........ S15

1,4-Di-*p*-tolylbutane-1,4-dione (**3u**) ........ S15

1,4-Bis(4-chlorophenyl)butane-1,4-dione (**3v**) ........ S16

1,4-Bis(4-fluorophenyl)butane-1,4-dione (**3w**) ........ S16

1,4-Bis(4-nitrophenyl)butane-1,4-dione (**3x**)....S17

1,4-Bis(4-(trifluoromethyl)phenyl)butane-1,4-dione (**3y**)....S17

1,4-Bis(3-methoxyphenyl)butane-1,4-dione (**3z**)....S18

1,4-Bis(3-fluorophenyl)butane-1,4-dione (**3aa**)....S18

1,4-Bis(3-chlorophenyl)butane-1,4-dione (**3ab**)....S19

1,4-Di(furan-2-yl)butane-1,4-dione (**3ac**)....S20

1,4-Di(thiophen-2-yl)butane-1,4-dione (**3ad**)....S20

2-(4-Methoxyphenyl)-5-phenyl-1*H*-pyrrole (**4**)....S21

2-(4-Methoxyphenyl)-5-phenylfuran (**5**)....S21

1-(4-Methoxyphenyl)-2-((2,2,6,6-tetramethylpiperidin-1-yl)oxy)ethan-1-one (**6**)....S22

**V. NMR spectra....S23**

1-(4-Methoxyphenyl)-4-phenylbutane-1,4-dione (**3a**)....S23

1-(4-Methoxyphenyl)-4-(*p*-tolyl)butane-1,4-dione (**3b**)....S24

1-(4-Bromophenyl)-4-(4-methoxyphenyl)butane-1,4-dione (**3c**)....S25

1-(4-Chlorophenyl)-4-(4-methoxyphenyl)butane-1,4-dione (**3d**)....S26

1-(4-Fluorophenyl)-4-(4-methoxyphenyl)butane-1,4-dione (**3e**)....S27

1-(3-Bromophenyl)-4-(4-methoxyphenyl)butane-1,4-dione (**3f**)....S29

1-(3-Chlorophenyl)-4-(4-methoxyphenyl)butane-1,4-dione (**3g**)....S30

1-(3-Fluorophenyl)-4-(4-methoxyphenyl)butane-1,4-dione (**3h**)....S31

1-(Furan-2-yl)-4-(4-methoxyphenyl)butane-1,4-dione (**3i**)....S33

1-(4-Methoxyphenyl)-4-(thiophen-2-yl)butane-1,4-dione (**3j**)....S34

1-Phenylpentane-1,4-dione (**3k**)....S35

1-(*p*-Tolyl)pentane-1,4-dione (**3l**)....S36

1-(4-Chlorophenyl)pentane-1,4-dione (**3m**)....S37

1-(4-Nitrophenyl)pentane-1,4-dione (**3n**)....S38

1-(3-Methoxyphenyl)pentane-1,4-dione (**3o**)....S39

1-(3-Chlorophenyl)pentane-1,4-dione (**3p**)....S40

1-(Thiophen-2-yl)pentane-1,4-dione (**3q**)....S41

2-(2-Oxo-2-phenylethyl)cyclopentan-1-one (**3r**)....S42

1,4-Bis(4-methoxyphenyl)butane-1,4-dione (**3s**)....S43

1,4-Diphenylbutane-1,4-dione (**3t**) ........ S44
1,4-Di-*p*-tolylbutane-1,4-dione (**3u**) ........ S45
1,4-Bis(4-chlorophenyl)butane-1,4-dione (**3v**) ........ S46
1,4-Bis(4-fluorophenyl)butane-1,4-dione (**3w**) ........ S47
1,4-Bis(4-nitrophenyl)butane-1,4-dione (**3x**) ........ S49
1,4-Bis(4-(trifluoromethyl)phenyl)butane-1,4-dione (**3y**) ........ S50
1,4-Bis(3-methoxyphenyl)butane-1,4-dione (**3z**) ........ S52
1,4-Bis(3-fluorophenyl)butane-1,4-dione (**3aa**) ........ S53
1,4-Bis(3-chlorophenyl)butane-1,4-dione (**3ab**) ........ S55
1,4-Di(furan-2-yl)butane-1,4-dione (**3ac**) ........ S56
1,4-Di(thiophen-2-yl)butane-1,4-dione (**3ad**) ........ S57
2-(4-Methoxyphenyl)-5-phenyl-1*H*-pyrrole (**4**) ........ S58
2-(4-Methoxyphenyl)-5-phenylfuran (**5**) ........ S59
1-(4-Methoxyphenyl)-2-((2,2,6,6-tetramethylpiperidin-1-yl)oxy)ethan-1-one (**6**) ........ S60
**VI. Detection of phenyltrimethylsilane (PhTMS) ........ S61**
**VII. References ........ S62**

## I. General information

All reagents were purchased from commercial sources and used without treatment unless otherwise indicated. The products were purified by column chromatography over silica gel. $^{1}H$ NMR and $^{13}C$ NMR spectra were recorded at 25 °C on a Varian spectrometer at 400 MHz and 101 MHz, respectively, with TMS as the internal standard. Mass spectra were recorded on BRUKER AutoflexIII Smartbeam MS-spectrometer. High-resolution mass spectra (HRMS) were recorded on Bruck microTof using an ESI-TOF method.

## II. Crystallography

Compound **3ab** (50 mg) was dissolved in a centrifuge tube with 150 µL $CDCl_3$. Upon standing for several days (seven days), crystals suitable for X-ray diffraction of **3ab** were obtained. The structures of the *N*-fused heterocyclic scaffolds were further established by X-ray diffraction. Single-crystal X-ray diffraction data for the reported compounds was recorded at a temperature of 296(2) K on an Oxford Diffraction Gemini R Ultra diffractometer using a ω scan technique with Mo-Kα radiation ($\lambda$ = 0.71073 Å). The structures were solved by the Direct Method of SHELXS-97 and refined by full-matrix least-squares techniques using the SHELXL-97 program.[1] Non-hydrogen atoms were refined with anisotropic temperature parameters, and hydrogen atoms of the ligands were refined as rigid groups. Basic information pertaining to crystal parameters and structure refinement is summarized in Table S1.

**Table S1.** Crystal structure and refinement data for compound **3ab** (thermal ellipsoids at 30% probability).

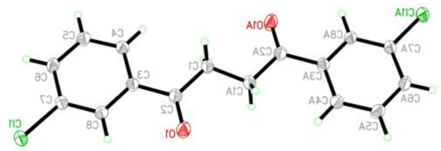

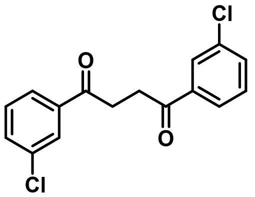


| | |
|---|---|
| Empirical formula | $C_{16}H_{12}Cl_2O_2$ |
| Temperature | 296(2) K |
| Wavelength | 0.71073 Å |
| Space group | P2(1)/c |
| Unit cell dimensions | a = 5.632(4) Å<br>b = 15.904(12) Å<br>c = 7.689(6) Å<br>alpha = 90 deg.<br>beta = 97.210(9) deg.<br>gamma = 90 deg. |
| Volume | 683.3(9) Å$^3$ |
| Z | 2 |
| Calculated density | 1.493 Mg/m$^3$ |
| Absorption coefficient | 0.472 mm$^{-1}$ |
| F(000) | 316 |
| Crystal size | 0.114 x 0.102 x 0.085 mm |
| Theta range for data collection | 2.56 to 25.00 deg. |
| Reflections collected / unique | 3335 / 1204 [R(int) = 0.0300] |
| Data / restraints / parameters | 1204 / 0 / 92 |
| Goodness-of-fit on F$^2$ | 1.077 |
| Final R indices [I>2sigma(I)] | R1 = 0.0320, wR2 = 0.0862 |
| R indices (all data) | R1 = 0.0329, wR2 = 0.0869 |

## III. Synthesis of compounds 1

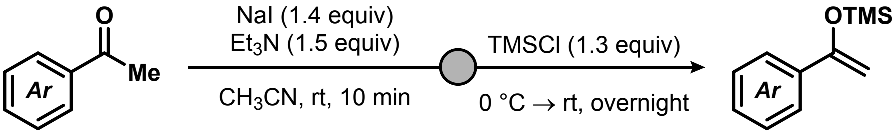


**General procedure for the preparation of aryl silyl enol ethers:**[2] NaI (210 mg, 1.4 mmol, 1.4 equiv) was placed in a tube and dried under vacuum using a heat gun. Upon cooling to room temperature, the tube was filled with argon. Then, dry $CH_3CN$ (1 mL), ketone (1 mmol, 1.0 equiv), and $Et_3N$ (210 *uL*, 1.5 mmol, 1.5 equiv) were successively added. The mixture was cooled with an ice/water bath, and TMSCl (166 µL, 1.3 mmol, 1.3 equiv) was added at 0 °C. The cooling bath was removed, and the mixture was stirred at room temperature for 12 h. Then, the volatile components were evaporated under vacuum. The solid residue was washed with petroleum ether (3 × 15 mL), the petroleum ether layers were decanted and filtered through a cotton plug. The combined filtrates were concentrated on a rotary evaporator, furnishing the silyl enol ether which was used without further purification.

***NOTE: All alkyl enol silyl enol ethers are commercially available.***

## IV. Synthesis and analytical data of compounds 3

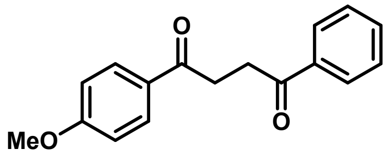


**1-(4-Methoxyphenyl)-4-phenylbutane-1,4-dione (3a)**[3]

To a 10 mL Schlenk tube equipped with a magnetic stir bar was added ((1-(4-methoxyphenyl)vinyl)oxy)trimethylsilane **1a** (144.5 mg, 0.65 mmol), trimethyl((1-phenylvinyl)oxy)silane **2a** (96 mg, 0.5 mmol), $CH_3CN$ (2.0 mL), PhBr (157 mg, 1.0 mmol), and AgF (13 mg, 0.1 mmol). The reaction mixture was stirred at 25 °C for about 6 h under argon. The resulting mixture was concentrated and the residue was taken up in ethyl acetate. The organic layer was washed with brine, dried over $Na_2SO_4$ and concentrated. Purification of the crude product by column chromatography (silica gel; petroleum ether/ethyl acetate 10:1) afforded **3a** in 69% yield (93 mg).

**Gram-scale synthesis of 1-(4-methoxyphenyl)-4-phenylbutane-1,4-dione (3a)**

To a 50 mL Schlenk tube equipped with a magnetic stir bar was added ((1-(4-methoxyphenyl)vinyl)oxy)trimethylsilane **1a** (1.445 g, 6.5 mmol), trimethyl((1-phenylvinyl)oxy)silane

**2a** (0.96 g, 5 mmol), $CH_3CN$ (20 mL), PhBr (1.57 g, 10 mmol), and AgF (130 mg, 1 mmol). The reaction mixture was stirred at 25 °C for about 12 h under argon. The resulting mixture was concentrated and the residue was taken up in ethyl acetate. The organic layer was washed with brine, dried over $Na_2SO_4$ and concentrated. Purification of the crude product by column chromatography (silica gel; petroleum ether/ethyl acetate 10:1) afforded **3a** in 65% yield (872 mg).

White solid; mp 99–100 °C; $^1$H NMR ($CDCl_3$, 400 MHz): $\delta_H$ 8.05–8.01 (m, 4H), 7.60–7.56 (m, 1H), 7.50–7.46 (m, 2H), 6.95 (d, *J* = 8.8 Hz, 2H), 3.80 (s, 3H), 3.46–3.41 (m 4H); $^{13}$C NMR ($CDCl_3$, 101 MHz): $\delta_C$ 198.9, 197.2, 163.5, 136.8, 133.1, 130.4, 129.8, 128.6, 128.1, 113.7, 55.5, 32.6, 32.2; HRMS (ESI-TOF, *m/z*): calcd for $C_{17}H_{17}O_3$ [M + H]$^+$, 269.1172; found, 269.1180.

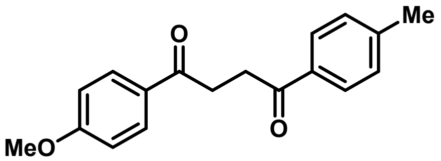


**1-(4-Methoxyphenyl)-4-(*p*-tolyl)butane-1,4-dione (3b)**[4]

To a 10 mL Schlenk tube equipped with a magnetic stir bar was added ((1-(4-methoxyphenyl)vinyl)oxy)trimethylsilane **1a** (144.5 mg, 0.65 mmol), trimethyl((1-(p-tolyl)vinyl)oxy)silane **2b** (103.2 mg, 0.5 mmol) $CH_3CN$ (2.0 mL), PhBr (157 mg, 1.0 mmol), and AgF (13 mg, 0.1 mmol). The reaction mixture was stirred at 25 °C for about 6 h under argon. The resulting mixture was concentrated and the residue was taken up in ethyl acetate. The organic layer was washed with brine, dried over $Na_2SO_4$ and concentrated. Purification of the crude product by column chromatography (silica gel; petroleum ether/ethyl acetate 10:1) afforded **3b** in 65% yield (91.8 mg).

White solid; mp 108–109 °C; $^1$H NMR ($CDCl_3$, 400 MHz): $\delta_H$ 8.02 (d, *J* = 8.4 Hz, 2H), 7.94 (d, *J* = 8.0 Hz, 2H), 7.27 (d, *J* = 8.0 Hz, 2H), 6.95 (d, *J* = 8.8 Hz, 2H), 3.88 (s, 3H), 3.45–3.40 (m, 4H), 2.42 (s, 3H); $^{13}$C NMR ($CDCl_3$, 101 MHz): $\delta_C$ 198.5, 197.3, 163.5, 143.9, 134.3, 130.4, 129.9, 128.2, 113.7, 55.5, 32.6, 32.3, 21.7; HRMS (ESI-TOF, *m/z*): calcd for $C_{18}H_{19}O_3$ [M + H]$^+$, 283.1329; found, 283.1334.

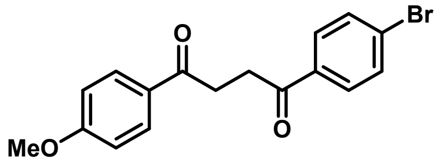

**1-(4-Bromophenyl)-4-(4-methoxyphenyl)butane-1,4-dione (3c)**

To a 10 mL Schlenk tube equipped with a magnetic stir bar was added ((1-(4-methoxyphenyl)vinyl)oxy)trimethylsilane **1a** (144.5 mg, 0.65 mmol), ((1-(4-bromophenyl)vinyl)oxy)trimethylsilane **2c** (135.6 mg, 0.5 mmol), $CH_3CN$ (2.0 mL), PhBr (157 mg, 1.0 mmol), and AgF (13 mg, 0.1 mmol). The reaction mixture was stirred at 25 °C for about 6 h under argon. The resulting mixture was concentrated and the residue was taken up in ethyl acetate. The organic layer was washed with brine, dried over $Na_2SO_4$ and concentrated. Purification of the crude product by column chromatography (silica gel; petroleum ether/ethyl acetate 10:1) afforded **3c** in 64% yield (111.1 mg).

White solid; mp 158–159 °C; $^1$H NMR ($CDCl_3$, 400 MHz): $\delta_H$ 8.01 (d, $J$ = 8.4 Hz, 2H), 7.90 (d, $J$ = 8.0 Hz, 2H), 7.62 (d, $J$ = 8.0 Hz, 2H), 6.95 (d, $J$ = 8.4 Hz, 2H), 3.88 (s, 3H), 3.43–3.38 (m, 4H); $^{13}$C NMR ($CDCl_3$, 101 MHz): $\delta_C$ 197.9, 197.0, 163.6, 135.6, 131.9, 130.4, 129.74, 129.67, 128.3, 113.7, 55.5, 32.5, 32.2; HRMS (ESI-TOF, $m/z$): calcd for $C_{17}H_{16}BrO_3$ $[M + H]^+$, 347.0277; found, 347.0264.

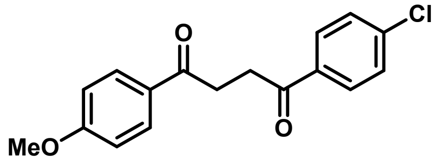

**1-(4-Chlorophenyl)-4-(4-methoxyphenyl)butane-1,4-dione (3d)**[8]

To a 10 mL Schlenk tube equipped with a magnetic stir bar was added ((1-(4-methoxyphenyl)vinyl)oxy)trimethylsilane **1a** (144.5 mg, 0.65 mmol), ((1-(4-chlorophenyl)vinyl)oxy)trimethylsilane **2d** (113.4 mg, 0.5 mmol), $CH_3CN$ (2.0 mL), PhBr (157 mg, 1.0 mmol), and AgF (13 mg, 0.1 mmol). The reaction mixture was stirred at 25 °C for about 6 h under argon. The resulting mixture was concentrated and the residue was taken up in ethyl acetate. The organic layer was washed with brine, dried over $Na_2SO_4$ and concentrated. Purification of the crude product by column chromatography (silica gel; petroleum ether/ethyl acetate 8:1) afforded **3d** in 69% yield (104 mg).

White solid; mp 132–134 °C; $^1$H NMR ($CDCl_3$, 400 MHz): $\delta_H$ 8.00 (dd, $J$ = 13.2, 8.4 Hz, 4H), 7.46 (d, $J$ = 8.4 Hz, 2H), 6.95 (d, $J$ = 8.8 Hz, 2H), 3.88 (s, 3H), 3.42–3.40 (m, 4H); $^{13}$C NMR ($CDCl_3$, 101 MHz): $\delta_C$ 197.7, 197.0, 163.6, 139.5, 135.2, 130.4, 129.8, 129.5, 128.9, 113.7, 55.5, 32.6, 32.2; HRMS (ESI-TOF, *m/z*): calcd for $C_{17}H_{16}ClO_3$ [M + H]$^+$, 303.0783; found, 303.0783.

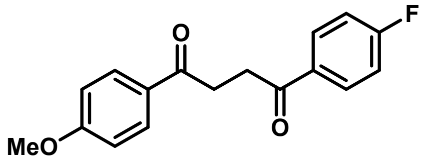


**1-(4-Fluorophenyl)-4-(4-methoxyphenyl)butane-1,4-dione (3e)**

To a 10 mL Schlenk tube equipped with a magnetic stir bar was added ((1-(4-methoxyphenyl)vinyl)oxy)trimethylsilane **1a** (144.5 mg, 0.65 mmol), ((1-(4-fluorophenyl)vinyl)oxy)trimethylsilane **2e** (105.2 mg, 0.5 mmol), $CH_3CN$ (2.0 mL), PhBr (157 mg, 1.0 mmol), and AgF (13 mg, 0.1 mmol). The reaction mixture was stirred at 25 °C for about 6 h under argon. The resulting mixture was concentrated and the residue was taken up in ethyl acetate. The organic layer was washed with brine, dried over $Na_2SO_4$ and concentrated. Purification of the crude product by column chromatography (silica gel; petroleum ether/ethyl acetate 10:1) afforded **3e** in 66% yield (94 mg).

White solid; mp 80–81 °C; $^1$H NMR ($CDCl_3$, 400 MHz): $\delta_H$ 8.07 (dd, $J$ = 8.4 , 5.6 Hz, 2H), 8.02 (d, $J$ = 8.8 Hz, 2H), 7.17–7.12 (m, 2H), 6.95 (d, $J$ = 8.8 Hz, 2H), 3.88 (s, 3H), 3.41 (s, 4H); $^{13}$C NMR ($CDCl_3$, 101 MHz): $\delta_C$ 197.3, 197.1, 165.8 (d, $J_{C–F}$ = 254.1 Hz), 163.6, 133.3 (d, $J_{C–F}$ = 3.1 Hz), 130.7 (d, $J_{C–F}$ = 9.2 Hz), 130.4, 129.8, 115.7 (d, $J_{C–F}$ = 21.7 Hz), 113.7, 55.5, 32.5, 32.2; $^{19}$F NMR ($CDCl_3$, 376 MHz): $\delta_F$ –105.3; HRMS (ESI-TOF, *m/z*): calcd for $C_{17}H_{16}FO_3$ [M + H]$^+$, 287.1078; found, 287.1094.

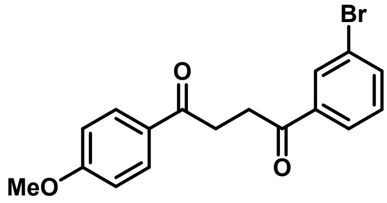


**1-(3-Bromophenyl)-4-(4-methoxyphenyl)butane-1,4-dione (3f)**

To a 10 mL Schlenk tube equipped with a magnetic stir bar was added ((1-(4-methoxyphenyl)vinyl)oxy)trimethylsilane **1a** (144.5 mg, 0.65 mmol), ((1-(3-bromophenyl)vinyl)oxy)trimethylsilane **2f** (135.6 mg, 0.5 mmol), $CH_3CN$ (2.0 mL), PhBr (157 mg, 1.0

mmol), and AgF (13 mg, 0.1 mmol). The reaction mixture was stirred at 25 °C for about 6 h under argon. The resulting mixture was concentrated and the residue was taken up in ethyl acetate. The organic layer was washed with brine, dried over $Na_2SO_4$ and concentrated. Purification of the crude product by column chromatography (silica gel; petroleum ether/ethyl acetate 10:1) afforded **3f** in 70% yield (122 mg).

White solid; mp 93–94 °C; $^1$H NMR ($CDCl_3$, 400 MHz): $\delta_H$ 8.16 (s, 1H), 8.02–7.95 (m, 3H), 7.70 (d, $J$ = 8.0 Hz, 1H), 7.36 (t, $J$ = 8.0 Hz, 1H), 6.95 (d, $J$ = 8.8 Hz, 2H), 3.88 (s, 3H), 3.42–3.40 (m, 4H); $^{13}$C NMR ($CDCl_3$, 101 MHz): $\delta_C$ 197.6, 196.9, 163.6, 138.6, 135.9, 131.2, 130.4, 130.2, 129.7, 126.7, 123.0, 113.7, 55.5, 32.7, 32.2; HRMS (ESI-TOF, *m/z*): calcd for $C_{17}H_{16}BrO_3$ [M + H]$^+$, 347.0277; found, 347.0264.

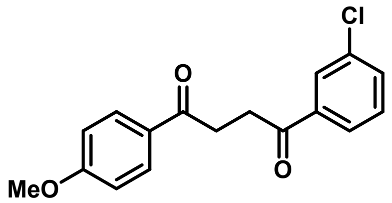


**1-(3-Chlorophenyl)-4-(4-methoxyphenyl)butane-1,4-dione (3g)**

To a 10 mL Schlenk tube equipped with a magnetic stir bar was added ((1-(4-methoxyphenyl)vinyl)oxy)trimethylsilane **1a** (144.5 mg, 0.65 mmol), ((1-(3-chlorophenyl)vinyl)oxy)trimethylsilane **2g** (113.4 mg, 0.5 mmol), $CH_3CN$ (2.0 mL), PhBr (157 mg, 1.0 mmol), and AgF (13 mg, 0.1 mmol). The reaction mixture was stirred at 25 °C for about 6 h under argon. The resulting mixture was concentrated and the residue was taken up in ethyl acetate. The organic layer was washed with brine, dried over $Na_2SO_4$ and concentrated. Purification of the crude product by column chromatography (silica gel; petroleum ether/ethyl acetate 10:1) afforded **3g** in 67% yield (101 mg).

White solid; mp 97–98 °C; $^1$H NMR ($CDCl_3$, 400 MHz): $\delta_H$ 8.03–8.01 (m, 3H), 7.92 (d, $J$ = 7.6 Hz, 1H), 7.55 (d, $J$ = 8.0 Hz, 1H), 7.45–7.41 (m, 1H), 6.96 (d, $J$ = 8.8 Hz, 2H), 3.88 (s, 3H), 3.43–3.41 (m 4H); $^{13}$C NMR ($CDCl_3$, 101 MHz): $\delta_C$ 197.7, 196.9, 163.6, 138.4, 134.9, 133.0, 130.4, 129.9, 129.7, 128.3, 126.2, 113.8, 55.5, 32.7, 32.2; HRMS (ESI-TOF, *m/z*): calcd for $C_{17}H_{16}ClO_3$ [M + H]$^+$, 303.0783; found, 303.0783.

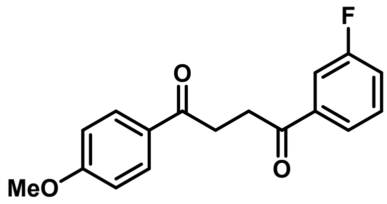

**1-(3-Fluorophenyl)-4-(4-methoxyphenyl)butane-1,4-dione (3h)**

To a 10 mL Schlenk tube equipped with a magnetic stir bar was added ((1-(4-methoxyphenyl)vinyl)oxy)trimethylsilane **1a** (144.5 mg, 0.65 mmol), ((1-(3-fluorophenyl)vinyl)oxy)trimethylsilane **2h** (105.2 mg, 0.5 mmol), $CH_3CN$ (2.0 mL), PhBr (157 mg, 1.0 mmol), and AgF (13 mg, 0.1 mmol). The reaction mixture was stirred at 25 °C for about 6 h under argon. The resulting mixture was concentrated and the residue was taken up in ethyl acetate. The organic layer was washed with brine, dried over $Na_2SO_4$ and concentrated. Purification of the crude product by column chromatography (silica gel; petroleum ether/ethyl acetate 13:1) afforded **3h** in 67% yield (95.9 mg).

White solid; mp 78–79 °C; $^1$H NMR ($CDCl_3$, 400 MHz): $\delta_H$ 8.01 (d, $J$ = 8.8 Hz, 2H), 7.83 (d, $J$ = 7.6 Hz, 1H), 7.71 (d, $J$ = 9.6 Hz, 1H), 7.49–7.44 (m, 1H), 7.30–7.26 (m, 1H), 6.95 (d, $J$ = 8.8 Hz, 2H), 3.88 (s, 3H), 3.42 (s, 4H); $^{13}$C NMR ($CDCl_3$, 101 MHz): $\delta_C$ 197.7, 196.9, 163.6, 162.9 (d, $J_{C–F}$ = 248.9 Hz), 138.9 (d, $J_{C–F}$ = 6.2 Hz), 130.3, 130.2 (d, $J_{C–F}$ = 7.8 Hz), 129.7, 123.9 (d, $J_{C–F}$ = 2.6 Hz), 120.1 (d, $J_{C–F}$ = 20.2 Hz), 114.8 (d, $J_{C–F}$ = 20.2 Hz), 113.7, 55.5, 32.7, 32.1; $^{19}$F NMR ($CDCl_3$, 376 MHz): $\delta_F$ –111.9; HRMS (ESI-TOF, *m/z*): calcd for $C_{17}H_{16}FO_3$ $[M + H]^+$, 287.1078; found, 287.1094.

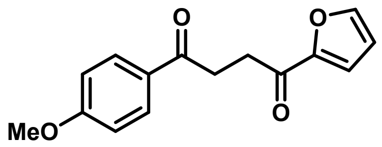

**1-(Furan-2-yl)-4-(4-methoxyphenyl)butane-1,4-dione (3i)**[4]

To a 10 mL Schlenk tube equipped with a magnetic stir bar was added ((1-(4-methoxyphenyl)vinyl)oxy)trimethylsilane **1a** (144.5 mg, 0.65 mmol), ((1-(furan-2-yl)vinyl)oxy)trimethylsilane **2i** (91.1 mg, 0.5 mmol), $CH_3CN$ (2.0 mL), PhBr (157 mg, 1.0 mmol), and AgF (13 mg, 0.1 mmol). The reaction mixture was stirred at 25 °C for about 6 h under argon. The resulting mixture was concentrated and the residue was taken up in ethyl acetate. The organic layer was washed with brine, dried over $Na_2SO_4$ and concentrated. Purification of the crude product by column chromatography (silica gel; petroleum ether/ethyl acetate 10:1) afforded **3i** in 74% yield (95.6 mg).

White solid; mp 94–95 °C; $^1$H NMR ($CDCl_3$, 400 MHz): $\delta_H$ 8.00 (d, $J$ = 8.4 Hz, 2H), 7.83 (d, $J$ = 3.6 Hz, 1H), 7.64 (d, $J$ = 4.8 Hz, 1H), 7.15 (t, $J$ = 4.4 Hz, 1H), 6.94 (d, $J$ = 8.4 Hz, 2H), 3.87 (s, 3H), 3.42–3.37 (m 4H); $^{13}$C NMR ($CDCl_3$, 101 MHz): $\delta_C$ 196.9, 191.8, 163.5, 143.9, 133.5, 132.0, 130.3, 129.7, 128.1, 113.7, 55.4, 33.2, 32.2; HRMS (ESI-TOF, $m/z$): calcd for $C_{15}H_{15}O_4$ $[M + H]^+$, 259.0965; found, 259.0984.

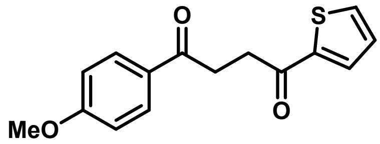

**1-(4-Methoxyphenyl)-4-(thiophen-2-yl)butane-1,4-dione (3j)**[4]

To a 10 mL Schlenk tube equipped with a magnetic stir bar was added ((1-(4-methoxyphenyl)vinyl)oxy)trimethylsilane **1a** (144.5 mg, 0.65 mmol), trimethyl((1-(thiophen-2-yl)vinyl)oxy)silane **2j** (99.2 mg, 0.5 mmol), $CH_3CN$ (2.0 mL), PhBr (157 mg, 1.0 mmol), and AgF (13 mg, 0.1 mmol). The reaction mixture was stirred at 25 °C for about 6 h under argon. The resulting mixture was concentrated and the residue was taken up in ethyl acetate. The organic layer was washed with brine, dried over $Na_2SO_4$ and concentrated. Purification of the crude product by column chromatography (silica gel; petroleum ether/ethyl acetate 10:1) afforded **3j** in 71% yield (97.4 mg).
White solid; mp 94–95 °C;$^1$H NMR ($CDCl_3$, 400 MHz): $\delta_H$ 8.00 (d, $J$ = 7.2 Hz, 2H), 7.83 (s, 1H), 7.64 (d, $J$ = 3.2 Hz, 1H), 7.16–7.14 (m, 1H), 6.94 (d, $J$ = 7.2 Hz, 2H), 3.87–3.86 (s, 3H), 3.43–3.36 (m, 4H); $^{13}$C NMR ($CDCl_3$, 101 MHz): $\delta_C$ 196.9, 191.8, 163.5, 143.9, 133.5, 132.0, 130.3, 129.7, 128.1, 113.7, 55.4, 33.2, 32.2; HRMS (ESI-TOF, $m/z$): calcd for $C_{15}H_{15}O_3S$ $[M + H]^+$, 275.0737; found, 275.0737.

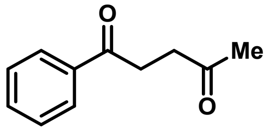

**1-Phenylpentane-1,4-dione (3k)**[4]

To a 10 mL dry Schlenk tube equipped with a magnetic stir bar was added trimethyl((1-phenylvinyl)oxy)silane **2a** (96.2 mg, 0.5 mmol), trimethyl(prop-1-en-2-yloxy)silane **2k** (195.4 mg, 1.5 mmol), $CH_3CN$ (2.0 mL), PhBr (157 mg, 1.0 mmol), and AgF (13 mg, 0.1 mmol). The reaction mixture was stirred at 25 °C for about 24 h under argon. The resulting mixture was concentrated and the residue was taken up in ethyl acetate. The organic layer was washed with brine, dried over $Na_2SO_4$

and concentrated. Purification of the crude product by column chromatography (silica gel; petroleum ether/ethyl acetate 13:1) afforded **3k** in 87% yield (76.7 mg).

Colorless oil; $^{1}$H NMR ($CDCl_3$, 400 MHz): $\delta_H$ 7.98 (d, *J* = 8.0 Hz, 2H), 7.58–7.55 (m, 1H), 7.48–7.44 (m, 2H), 3.28 (t, *J* = 6.4 Hz, 2H), 2.90 (t, *J* = 6.4 Hz, 2H), 2.26 (s, 3H); $^{13}$C NMR ($CDCl_3$, 101 MHz): $\delta_C$ 207.3, 198.5, 136.6, 133.1, 128.5, 128.0, 37.0, 32.4, 30.1; HRMS (ESI-TOF, *m/z*): calcd for $C_{11}H_{13}O_2$ $[M + H]^+$, 177.0910; found, 177.0913.

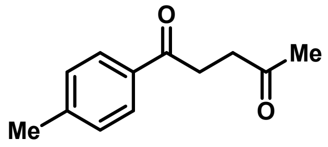

**1-(*p*-Tolyl)pentane-1,4-dione (3l)**[5]

To a 10 mL dry Schlenk tube equipped with a magnetic stir bar was added trimethyl((1-(p-tolyl)vinyl)oxy)silane **2b** (103.2 mg, 0.5 mmol), trimethyl(prop-1-en-2-yloxy)silane **2k** (195.4 mg, 1.5 mmol), $CH_3CN$ (2.0 mL), PhBr (157 mg, 1.0 mmol), and AgF (13 mg, 0.1 mmol). The reaction mixture was stirred at 25 °C for about 24 h under argon. The resulting mixture was concentrated and the residue was taken up in ethyl acetate. The organic layer was washed with brine, dried over $Na_2SO_4$ and concentrated. Purification of the crude product by column chromatography (silica gel; petroleum ether/ethyl acetate 19:1) afforded **3l** in 89% yield (85 mg).

Colorless oil; $^{1}$H NMR ($CDCl_3$, 400 MHz): $\delta_H$ 7.88 (d, *J* = 7.6 Hz, 2H), 7.25 (d, *J* = 8.0 Hz, 2H), 3.25 (t, *J* = 6.4 Hz, 2H), 2.87 (t, *J* = 6.0 Hz, 2H), 2.41 (s, 3H), 2.26 (s, 3H); $^{13}$C NMR ($CDCl_3$, 101 MHz): $\delta_C$ 207.4, 198.1, 143.9, 134.1, 129.2, 128.1, 37.0, 32.3, 30.1. 21.6; HRMS (ESI-TOF, *m/z*): calcd for $C_{12}H_{15}O_2$ $[M + H]^+$, 191.1067; found, 191.1081.

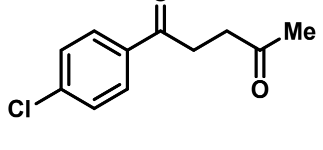

**1-(4-Chlorophenyl)pentane-1,4-dione (3m)**[6]

To a 10 mL dry Schlenk tube equipped with a magnetic stir bar was added ((1-(4-chlorophenyl)vinyl)oxy)trimethylsilane **2d** (113.4 mg, 1.5 mmol), trimethyl(prop-1-en-2-yloxy)silane **2k** (195.4 mg, 1.5 mmol), $CH_3CN$ (2.0 mL), PhBr (157 mg, 1.0 mmol), and AgF (13 mg, 0.1 mmol). The

reaction mixture was stirred at 25 °C for about 24 h under argon. The resulting mixture was concentrated and the residue was taken up in ethyl acetate. The organic layer was washed with brine, dried over $Na_2SO_4$ and concentrated. Purification of the crude product by column chromatography (silica gel; petroleum ether/ethyl acetate 10:1) afforded **3m** in 81% yield (85 mg).

Colorless oil; $^1$H NMR ($CDCl_3$, 400 MHz): $\delta_H$ 7.92 (d, $J$ = 8.4 Hz, 2H), 7.44 (d, $J$ = 8.0 Hz, 2H), 3.24 (t, $J$ = 6.4 Hz, 2H), 2.89 (t, $J$ = 6.0 Hz, 2H), 2.26 (s, 3H); $^{13}$C NMR ($CDCl_3$, 101 MHz): $\delta_C$ 207.1, 197.3, 139.6, 134.9, 129.4, 128.9, 37.0, 32.3, 30.0; HRMS (ESI-TOF, *m/z*): calcd for $C_{11}H_{12}ClO_2$ $[M + H]^+$, 211.0520; found, 211.0536.

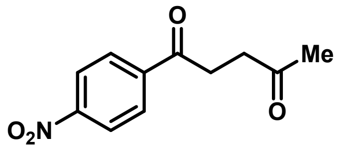


**1-(4-Nitrophenyl)pentane-1,4-dione (3n)**[6]

To a 10 mL dry Schlenk tube equipped with a magnetic stir bar was added trimethyl((1-(4-nitrophenyl)vinyl)oxy)silane **1b** (118.7 mg, 0.5 mmol), trimethyl(prop-1-en-2-yloxy)silane **2k** (193.4 mg,1.5 mmol), $CH_3CN$ (2.0 mL), PhBr (157 mg, 1.0 mmol), and AgF (13 mg, 0.1 mmol). The reaction mixture was stirred at 25 °C for about 24 h under argon. The resulting mixture was concentrated and the residue was taken up in ethyl acetate. The organic layer was washed with brine, dried over $Na_2SO_4$ and concentrated. Purification of the crude product by column chromatography (silica gel; petroleum ether/ethyl acetate 8:1) afforded **3n** in 72% yield (80 mg).

Yellow oil; $^1$H NMR ($CDCl_3$, 400 MHz): $\delta_H$ 8.32 (d, $J$ = 8.4 Hz, 2H), 8.14 (d, $J$ = 8.8 Hz, 2H), 3.29 (t, $J$ = 6.0 Hz, 2H), 2.95 (t, $J$ = 6.0 Hz, 2H), 2.28 (s, 3H); $^{13}$C NMR ($CDCl_3$, 101 MHz): $\delta_C$ 206.8, 197.1, 150.3, 141.1, 129.1, 123.8, 37.0, 32.8, 29.9; HRMS (ESI-TOF, *m/z*): calcd for $C_{11}H_{12}NO_4$ $[M + H]^+$, 222.0761; found, 222.0740.

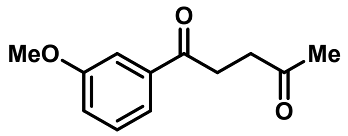

**1-(3-Methoxyphenyl)pentane-1,4-dione (3o)**[4]

To a 10 mL dry Schlenk tube equipped with a magnetic stir bar was added ((1-(3-methoxyphenyl)vinyl)oxy)trimethylsilane **1c** (111.2 mg, 0.5 mmol), trimethyl(prop-1-en-2-yloxy)silane **2k** (195.4 mg, 1.5 mmol), $CH_3CN$ (2.0 mL), PhBr (157 mg, 1.0 mmol), and AgF (13 mg, 0.1 mmol). The reaction mixture was stirred at 25 °C for about 24 h under argon. The resulting mixture was concentrated and the residue was taken up in ethyl acetate. The organic layer was washed with brine, dried over $Na_2SO_4$ and concentrated. Purification of the crude product by column chromatography (silica gel; petroleum ether/ethyl acetate 10:1) afforded **3o** in 88% yield (91 mg).

Colorless oil; $^1$H NMR ($CDCl_3$, 400 MHz): $\delta_H$ 7.58 (d, *J* = 8.0 Hz, 1H), 7.49 (s,1H), 7.39–7.35 (m, 1H), 7.12–7.01 (m, 1H), 3.85 (s,3H), 3.27 (t, *J* = 6.0 Hz, 2H), 2.88 (t, *J* = 6.4 Hz, 2H), 2.26 (s, 3H); $^{13}$C NMR ($CDCl_3$, 101 MHz): $\delta_C$ 207.3, 198.4, 159.8, 137.9, 129.6, 120.7, 119.7, 112.2, 55.4, 37.1, 32.5, 30.1; HRMS (ESI-TOF, *m/z*): calcd for $C_{12}H_{15}O_3$ $[M + H]^+$, 207.1016; found, 207.1018.

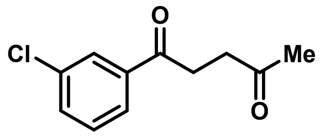

**1-(3-Chlorophenyl)pentane-1,4-dione (3p)**[7]

To a 10 mL dry Schlenk tube equipped with a magnetic stir bar was added ((1-(3-chlorophenyl)vinyl)oxy)trimethylsilane **2g** (113.4 mg, 0.5 mmol), trimethyl(prop-1-en-2-yloxy)silane **2k** (195.4 mg, 1.5 mmol), $CH_3CN$ (2.0 mL), PhBr (157 mg, 1.0 mmol), and AgF (13 mg, 0.1 mmol). The reaction mixture was stirred at 25 °C for about 24 h under argon. The resulting mixture was concentrated and the residue was taken up in ethyl acetate. The organic layer was washed with brine, dried over $Na_2SO_4$ and concentrated. Purification of the crude product by column chromatography (silica gel; petroleum ether/ethyl acetate 10:1) afforded **3p** in 83% yield (87 mg).

Colorless oil; $^1$H NMR ($CDCl_3$, 400 MHz): $\delta_H$ 7.95 (s, 1H), 7.86 (d, *J* = 7.6 Hz, 1H), 7.54 (d, *J* = 8.0 Hz, 1H), 7.43–7.40 (m, 1H), 3.24 (t, *J* = 6.0 Hz, 2H), 2.90 (t, *J* = 6.4 Hz, 2H), 2.26 (s, 3H); $^{13}$C NMR ($CDCl_3$, 101

MHz): $\delta_C$ 207.0, 197.3, 138.1, 134.9, 133.1, 129.9, 128.2, 126.1, 36.9, 32.4, 30.0; HRMS (ESI-TOF, *m/z*): calcd for $C_{11}H_{12}ClO_2$ [M + H]$^+$, 211.0520; found, 211.0536.

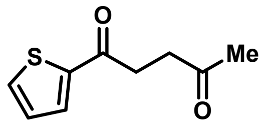

**1-(Thiophen-2-yl)pentane-1,4-dione (3q)**[4]

To a 10 mL dry Schlenk tube equipped with a magnetic stir bar was added trimethyl((1-(thiophen-2-yl)vinyl)oxy)silane **2j** (99.2 mg, 0.5 mmol), trimethyl(prop-1-en-2-yloxy)silane **2k** (195.4 mg, 1.5 mmol), $CH_3CN$ (2.0 mL), PhBr (157 mg, 1.0 mmol), and AgF (13 mg, 0.1 mmol). The reaction mixture was stirred at 25 °C for about 24 h under argon. The resulting mixture was concentrated and the residue was taken up in ethyl acetate. The organic layer was washed with brine, dried over $Na_2SO_4$ and concentrated. Purification of the crude product by column chromatography (silica gel; petroleum ether/ethyl acetate 11:1) afforded **3q** in 77% yield (70 mg).

Colorless oil; $^1$H NMR ($CDCl_3$, 400 MHz): $\delta_H$ 7.77 (d, *J* = 3.6 Hz, 1H), 7.63 (d, *J* = 4.8 Hz, 1H), 7.14–7.12 (m, 1H), 3.22 (t, *J* = 6.4 Hz, 2H), 2.88 (t, *J* = 6.4 Hz, 2H), 2.24 (s, 3H); $^{13}$C NMR ($CDCl_3$, 101 MHz): $\delta_C$ 207.1, 191.4, 143.7, 133.5, 132.0, 128.1, 37.0, 32.9, 30.0; HRMS (ESI-TOF, *m/z*): calcd for $C_9H_{11}O_2S$ [M + H]$^+$, 183.0474; found, 183.0480.

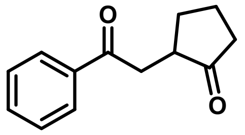

**2-(2-Oxo-2-phenylethyl)cyclopentan-1-one (3r)**[7]

To a 10 mL dry Schlenk tube equipped with a magnetic stir bar was added trimethyl((1-phenylvinyl)oxy)silane **2a** (96.2 mg, 0.5 mmol), (cyclopent-1-en-1-yloxy)trimethylsilane **2l** (276.7 mg,1.5 mmol), $CH_3CN$ (2.0 mL), PhBr (157 mg, 1.0 mmol), and AgF (13 mg, 0.1 mmol). The reaction mixture was stirred at 25 °C for about 24 h under argon. The resulting mixture was concentrated and the residue was taken up in ethyl acetate. The organic layer was washed with brine, dried over $Na_2SO_4$ and concentrated. Purification of the crude product by column chromatography (silica gel; petroleum ether/ethyl acetate 10:1) afforded **3r** in 86% yield (87 mg).

Colorless oil; $^{1}$H NMR ($CDCl_3$, 400 MHz): $\delta_H$ 7.96 (d, $J$ = 8.0 Hz, 2H), 7.59–7.55 (m, 1H), 7.48–7.45 (m, 2H), 3.56–3.51 (m, 1H), 3.06–3.02 (m, 1H), 2.69–2.61 (m, 1H), 2.44–2.24 (m, 3H), 2.13–2.06 (m, 1H), 1.91–1.38 (m, 1H), 1.66–1.62 (m, 1H); $^{13}$C NMR ($CDCl_3$, 101 MHz): $\delta_C$ 220.4, 198.0, 136.6, 133.2, 128.6, 128.0, 45.1, 38.6, 37.5, 29.7, 20.8; HRMS (ESI-TOF, *m/z*): calcd for $C_{13}H_{15}O_2$ $[M + H]^+$, 203.1067; found, 203.1082.

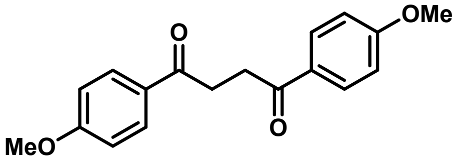


**1,4-Bis(4-methoxyphenyl)butane-1,4-dione (3s)**[8]

To a 10 mL Schlenk tube equipped with a magnetic stir bar was added ((1-(4-methoxyphenyl)vinyl)oxy)trimethylsilane **1a** (222.4 mg, 1.0 mmol), $CH_3CN$ (2.0 mL), PhBr (157 mg, 1.0 mmol), and AgF (13 mg, 0.1 mmol). The reaction mixture was stirred at 25 °C for about 6 h under argon. The resulting mixture was concentrated and the residue was taken up in ethyl acetate. The organic layer was washed with brine, dried over $Na_2SO_4$ and concentrated. Purification of the crude product by column chromatography (silica gel; petroleum ether/ethyl acetate 8:1) afforded **3s** in 93% yield (139 mg).

White solid; mp 150–152 °C; $^{1}$H NMR ($CDCl_3$, 400 MHz): $\delta_H$ 8.03 (d, $J$ = 8.8 Hz, 4H), 6.95 (d, $J$ = 8.8 Hz, 4H), 3.88 (s, 6H), 3.41 (s, 4H); $^{13}$C NMR ($CDCl_3$, 101 MHz): $\delta_C$ 197.4, 163.5, 130.4, 129.9, 113.7, 55.5, 32.3; HRMS (ESI-TOF, *m/z*): calcd for $C_{18}H_{19}O_4$ $[M + H]^+$, 299.1278; found, 299.1284.

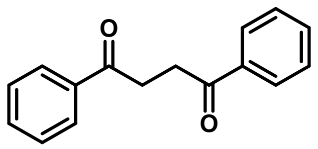

**1,4-Diphenylbutane-1,4-dione (3t)**[3]

To a 10 mL Schlenk tube equipped with a magnetic stir bar was added trimethyl((1-phenylvinyl)oxy)silane **2a** (192.3 mg, 1.0 mmol), $CH_3CN$ (2.0 mL), PhBr (157 mg, 1.0 mmol), and AgF (13 mg, 0.1 mmol). The reaction mixture was stirred at 25 °C for about 6 h under argon. The resulting mixture was concentrated and the residue was taken up in ethyl acetate. The organic layer was washed with brine, dried over $Na_2SO_4$ and concentrated. Purification of the crude product by column chromatography (silica gel; petroleum ether/ethyl acetate 13:1) afforded **3t** in 87% yield (104 mg). White solid; mp 142–146 °C; $^1$H NMR ($CDCl_3$, 400 MHz): $\delta_H$ 8.05 (d, $J$ = 8.0 Hz, 4H), 7.60–7.57 (m, 2H), 7.51–7.47 (m, 4H), 3.74 (s, 4H); $^{13}$C NMR ($CDCl_3$, 101 MHz): $\delta_C$ 198.7, 136.7, 133.2, 128.6, 128.1, 32.6; HRMS (ESI-TOF, *m/z*): calcd for $C_{16}H_{15}O_2$ $[M + H]^+$, 239.1067; found, 239.1085.

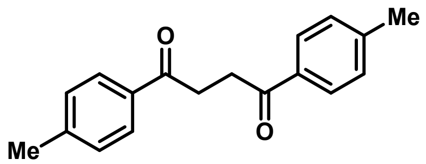

**1,4-Di-*p*-tolylbutane-1,4-dione (3u)**[9]

To a 10 mL Schlenk tube equipped with a magnetic stir bar was added trimethyl((1-(p-tolyl)vinyl)oxy)silane **2b** (206.4 mg, 1.0 mmol), $CH_3CN$ (2.0 mL), PhBr (157 mg, 1.0 mmol), and AgF (13 mg, 0.1 mmol). The reaction mixture was stirred at 25 °C for about 6 h under argon. The resulting mixture was concentrated and the residue was taken up in ethyl acetate. The organic layer was washed with brine, dried over $Na_2SO_4$ and concentrated. Purification of the crude product by column chromatography (silica gel; petroleum ether/ethyl acetate 14:1) afforded **3u** in 82% yield (112 mg). White solid; mp 159–160 °C; $^1$H NMR ($CDCl_3$, 400 MHz): $\delta_H$ 7.94 (d, $J$ = 8.0 Hz, 4H), 7.27 (d, $J$ = 8.0 Hz, 4H), 3.42 (s, 4H), 2.41 (s, 6H); $^{13}$C NMR ($CDCl_3$, 101 MHz): $\delta_C$ 198.4, 143.9, 134.3, 129.2, 128.2, 32.5, 21.6; HRMS (ESI-TOF, *m/z*): calcd for $C_{18}H_{19}O_2$ $[M + H]^+$, 267.1380; found, 267.1387.

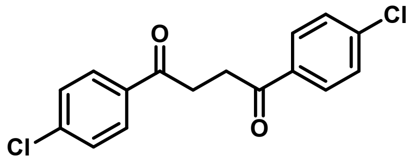

**1,4-Bis(4-chlorophenyl)butane-1,4-dione (3v)**[8]

To a 10 mL Schlenk tube equipped with a magnetic stir bar was added ((1-(4-chlorophenyl)vinyl)oxy)trimethylsilane **2d** (226.8 mg, 1.0 mmol), $CH_3CN$ (2.0 mL), PhBr (157 mg, 1.0 mmol), and AgF (13 mg, 0.1 mmol). The reaction mixture was stirred at 25 °C for about 6 h under argon. The resulting mixture was concentrated and the residue was taken up in ethyl acetate. The organic layer was washed with brine, dried over $Na_2SO_4$ and concentrated. Purification of the crude product by column chromatography (silica gel; petroleum ether/ethyl acetate 10:1) afforded **3v** in 86% yield (132 mg).

White solid; mp 147–149 °C; $^1$H NMR ($CDCl_3$, 400 MHz): $\delta_H$ 7.94 (d, $J$ = 8.0 Hz, 4H), 7.46 (d, $J$ = 8.0 Hz, 4H), 3.42 (s, 4H); $^{13}$C NMR ($CDCl_3$, 101 MHz): $\delta_C$ 197.3, 139.7, 135.0, 129.5, 128.9, 32.5; HRMS (ESI-TOF, *m/z*): calcd for $C_{16}H_{13}Cl_2O_2$ $[M + H]^+$, 307.0287; found, 307.0289.

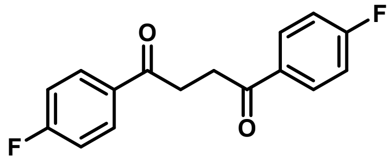

**1,4-Bis(4-fluorophenyl)butane-1,4-dione (3w)**[8]

To a 10 mL Schlenk tube equipped with a magnetic stir bar was added ((1-(4-fluorophenyl)vinyl)oxy)trimethylsilane **2e** (210.3 mg, 1.0 mmol), $CH_3CN$ (2.0 mL), PhBr (157 mg, 1.0 mmol), and AgF (13 mg, 0.1 mmol). The reaction mixture was stirred at 25 °C for about 6 h under argon. The resulting mixture was concentrated and the residue was taken up in ethyl acetate. The organic layer was washed with brine, dried over $Na_2SO_4$ and concentrated. Purification of the crude product by column chromatography (silica gel; petroleum ether/ethyl acetate 12:1) afforded **3w** in 86% yield (118 mg).

White solid; mp 141–143 °C; $^1$H NMR ($CDCl_3$, 400 MHz): $\delta_H$ 8.06 (dd, $J$ = 8.4, 5.6 Hz, 4H), 7.17–7.13 (m, 4H), 3.43 (s, 4H); $^{13}$C NMR ($CDCl_3$, 101 MHz): $\delta_C$ 197.0, 165.8 (d, $J_{C\text{–}F}$ = 255.9 Hz), 133.1 (d, $J_{C\text{–}F}$ = 2.7 Hz), 130.7 (d, $J_{C\text{–}F}$ = 9.5 Hz), 115.7 (d, $J_{C\text{–}F}$ = 21.9 Hz), 32.4; $^{19}$F NMR ($CDCl_3$, 376 MHz): $\delta_F$ –105.1; HRMS (ESI-TOF, *m/z*): calcd for $C_{16}H_{13}F_2O_2$ $[M + H]^+$, 275.0878; found, 275.0875.

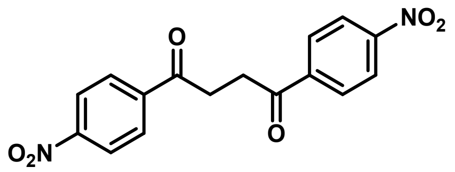

**1,4-Bis(4-nitrophenyl)butane-1,4-dione (3x)**[10]

To a 10 mL Schlenk tube equipped with a magnetic stir bar was added trimethyl((1-(4-nitrophenyl)vinyl)oxy)silane **1b** (237.3 mg, 1.0 mmol), $CH_3CN$ (2.0 mL), PhBr (157 mg, 1.0 mmol), and AgF (13 mg, 0.1 mmol). The reaction mixture was stirred at 25 °C for about 6 h under argon. The resulting mixture was concentrated and the residue was taken up in ethyl acetate. The organic layer was washed with brine, dried over $Na_2SO_4$ and concentrated. Purification of the crude product by column chromatography (silica gel; petroleum ether/ethyl acetate 5:1) afforded **3x** in 73% yield (120 mg).

Yellow solid; mp 195–196 °C; $^1$H NMR ($CDCl_3$, 400 MHz): $\delta_H$ 8.36 (d, $J$ = 8.4 Hz, 4H), 8.20 (d, $J$ = 8.4 Hz, 4H), 3.53 (s, 4H); $^{13}$C NMR ($CDCl_3$, 101 MHz): $\delta_C$ 196.8, 150.5, 140.9, 129.2, 123.9, 33.0; HRMS (ESI-TOF, *m/z*): calcd for $C_{16}H_{13}N_2O_6$ [M + H]$^+$, 329.0768; found, 329.0762.

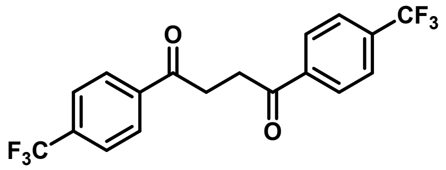

**1,4-Bis(4-(trifluoromethyl)phenyl)butane-1,4-dione (3y)**

To a 10 mL Schlenk tube equipped with a magnetic stir bar was added trimethyl((1-(4-(trifluoromethyl)phenyl)vinyl)oxy)silane **1d** (260.3 mg, 1.0 mmol), $CH_3CN$ (2.0 mL), PhBr (157 mg, 1.0 mmol), and AgF (13 mg, 0.1 mmol). The reaction mixture was stirred at 25 °C for about 6 h under argon. The resulting mixture was concentrated and the residue was taken up in ethyl acetate. The organic layer was washed with brine, dried over $Na_2SO_4$ and concentrated. Purification of the crude product by column chromatography (silica gel; petroleum ether/ethyl acetate 14:1) afforded **3y** in 71% yield (133mg).

White solid; mp 136–137 °C; $^1$H NMR ($CDCl_3$, 400 MHz): $\delta_H$ 8.14 (d, $J$ = 8.0 Hz, 4H), 7.76 (d, $J$ = 8.0 Hz, 4H), 3.50 (s, 4H); $^{13}$C NMR ($CDCl_3$, 101 MHz): $\delta_C$ 197.5, 139.2, 134.6 (q, $J_{C-F}$ = 32.3 Hz), 128.4, 125.7 (d,

$J_{C–F}$ = 3.6 Hz), 123.6 (q, $J_{C–F}$ = 274.0 Hz), 32.7; $^{19}$F NMR ($CDCl_3$, 376 MHz): $\delta_F$ –63.1; HRMS (ESI-TOF, *m/z*): calcd for $C_{18}H_{13}F_6O_2$ [M + H]$^+$, 375.0814; found, 375.0809.

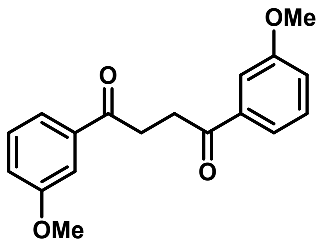

**1,4-Bis(3-methoxyphenyl)butane-1,4-dione (3z)**[11]

To a 10 mL Schlenk tube equipped with a magnetic stir bar was added ((1-(3-methoxyphenyl)vinyl)oxy)trimethylsilane **1c** (260.3 mg, 1.0 mmol), $CH_3CN$ (2.0 mL), PhBr (157 mg, 1.0 mmol), and AgF (13 mg, 0.1 mmol). The reaction mixture was stirred at 25 °C for about 6 h under argon. The resulting mixture was concentrated and the residue was taken up in ethyl acetate. The organic layer was washed with brine, dried over $Na_2SO_4$ and concentrated. Purification of the crude product by column chromatography (silica gel; petroleum ether/ethyl acetate 10:1) afforded **3z** in 84% yield (125 mg).

White solid; mp 128–129 °C; $^1$H NMR ($CDCl_3$, 400 MHz): $\delta_H$ 7.64 (d, $J$ = 7.6 Hz, 2H), 7.54 (s, 2H), 7.41–7.37 (m, 2H), 7.13 (dd, $J$= 8.0 Hz and 2.4 Hz, 2H), 3.86 (s, 6H), 3.44 (s, 4H); $^{13}$C NMR ($CDCl_3$, 101 MHz): $\delta_C$ 198.5, 159.8, 138.1, 129.6, 120.8, 119.7, 112.2, 55.4, 32.7; HRMS (ESI-TOF, *m/z*): calcd for $C_{18}H_{19}O_4$ [M + H]$^+$, 299.1278; found, 299.1284.

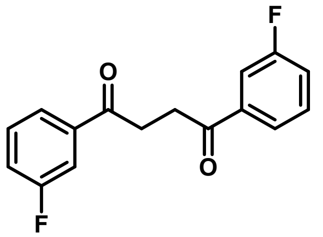

**1,4-Bis(3-fluorophenyl)butane-1,4-dione (3aa)**

To a 10 mL Schlenk tube equipped with a magnetic stir bar was added ((1-(3-fluorophenyl)vinyl)oxy)trimethylsilane **2h** (210.3 mg, 1.0 mmol), $CH_3CN$ (2.0 mL), PhBr (157 mg, 1.0 mmol), and AgF (13 mg, 0.1 mmol). The reaction mixture was stirred at 25 °C for about 6 h under argon. The resulting mixture was concentrated and the residue was taken up in ethyl acetate. The organic layer was washed with brine, dried over $Na_2SO_4$ and concentrated. Purification of the crude

product by column chromatography (silica gel; petroleum ether/ethyl acetate 10:1) afforded **3aa** in 88% yield (121mg).

White solid; mp 118–119 °C; $^1$H NMR ($CDCl_3$, 400 MHz): $\delta_H$ 7.82 (d, $J$ =8.0 Hz, 2H), 7.80 (d, $J$ = 9.2 Hz, 2H), 7.49–7.44(m, 2H), 7.30–7.26 (m, 2H), 3.43 (s, 4H); $^{13}$C NMR ($CDCl_3$, 101 MHz): $\delta_C$ 197.2), 162.8 (d, $J_{C–F}$ = 249.0 Hz), 138.7 (d, $J_{C–F}$ = 6.3 Hz), 130.3 (d, $J_{C–F}$ = 7.6 Hz), 123.9 (d, $J_{C–F}$ = 2.7 Hz), 120.2 (d, $J_{C–F}$ = 21.6 Hz), 114.8 (d, $J_{C–F}$ = 22.4 Hz), 32.6; $^{19}$F NMR ($CDCl_3$, 376 MHz): $\delta_F$ –111.8; HRMS (ESI-TOF, $m/z$): calcd for $C_{16}H_{13}F_2O_2$ $[M + H]^+$, 275.0878; found, 275.0875.

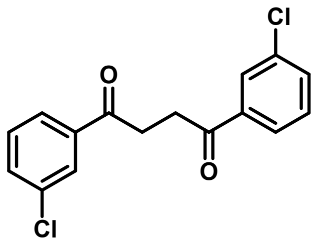


**1,4-Bis(3-chlorophenyl)butane-1,4-dione (3ab)**[11]

To a 10 mL Schlenk tube equipped with a magnetic stir bar was added ((1-(3-chlorophenyl)vinyl)oxy)trimethylsilane **2g** (226.8 mg, 1.0 mmol), $CH_3CN$ (2.0 mL), PhBr (157 mg, 1.0 mmol), and AgF (13 mg, 0.1 mmol). The reaction mixture was stirred at 25 °C for about 6 h under argon. The resulting mixture was concentrated and the residue was taken up in ethyl acetate. The organic layer was washed with brine, dried over $Na_2SO_4$ and concentrated. Purification of the crude product by column chromatography (silica gel; petroleum ether/ethyl acetate 12:1) afforded **3ab** in 88% yield (135 mg).

White solid; mp 142–143 °C; $^1$H NMR ($CDCl_3$, 400 MHz): $\delta_H$ 8.00 (s, 2H), 7.91 (d, $J$ = 8.0 Hz, 2H), 7.56 (d, $J$ = 8.0 Hz, 2H), 7.45–7.41 (m, 2H), 3.43 (s, 4H); $^{13}$C NMR ($CDCl_3$, 101 MHz): $\delta_C$ 197.2, 138.1, 135.0, 133.1, 130.0, 128.2, 126.2, 32.6; HRMS (ESI-TOF, $m/z$): calcd for $C_{16}H_{13}Cl_2O_2$ $[M + H]^+$, 307.0287; found, 307.0289.

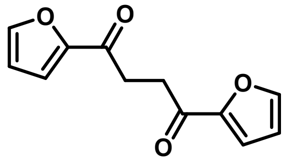

**1,4-Di(furan-2-yl)butane-1,4-dione (3ac)**[12]

To a 10 mL Schlenk tube equipped with a magnetic stir bar was added ((1-(furan-2-yl)vinyl)oxy)trimethylsilane **2i** (182.3 mg, 1 mmol), $CH_3CN$ (2.0 mL), PhBr (157 mg, 1 mmol), and AgF (13 mg, 0.1 mmol). The reaction mixture was stirred at 25 °C for about 6 h under argon. The resulting mixture was concentrated and the residue was taken up in ethyl acetate. The organic layer was washed with brine, dried over $Na_2SO_4$ and concentrated. Purification of the crude product by column chromatography (silica gel; petroleum ether/ethyl acetate 10:1) afforded **3ac** in 83% yield (91 mg).

White solid; mp 134–136 °C; $^1$H NMR ($CDCl_3$, 400 MHz): $\delta_H$ 7.82 (d, *J* = 3.6 Hz, 2H), 7.65 (d, *J* = 4.8 Hz, 2H), 7.16–7.14 (m, 2H), 3.40 (s, 4H); $^{13}$C NMR ($CDCl_3$, 101 MHz): $\delta_C$ 191.4, 143.7, 133.6, 132.1, 128.1, 33.1; HRMS (ESI-TOF, *m/z*): calcd for $C_{12}H_{11}O_4$ $[M + H]^+$, 219.0652; found, 219.0673.

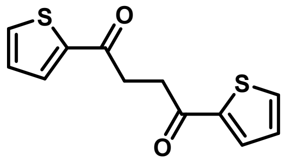

**1,4-Di(thiophen-2-yl)butane-1,4-dione (3ad)**[12]

To a 10 mL Schlenk tube equipped with a magnetic stir bar was added trimethyl((1-(thiophen-2-yl)vinyl)oxy)silane **2j** (198.4 mg, 1 mmol), $CH_3CN$ (2.0 mL), PhBr (157 mg, 1 mmol), and AgF (13 mg, 0.1 mmol). The reaction mixture was stirred at 25 °C for about 6 h under argon. The resulting mixture was concentrated and the residue was taken up in ethyl acetate. The organic layer was washed with brine, dried over $Na_2SO_4$ and concentrated. Purification of the crude product by column chromatography (silica gel; petroleum ether/ethyl acetate 10:1) afforded **3ad** in 87% yield (109 mg).

White solid; mp 136–138 °C; $^1$H NMR ($CDCl_3$, 400 MHz): $\delta_H$ 7.82 (d, *J* = 3.6 Hz, 2H), 7.65 (d, *J* = 4.8 Hz, 2H), 7.16–7.14 (m, 2H), 3.40 (s, 4H); $^{13}$C NMR ($CDCl_3$, 101 MHz): $\delta_C$ 191.4, 143.7, 133.6, 132.1, 128.1, 33.2; HRMS (ESI-TOF, *m/z*): calcd for $C_{12}H_{11}O_2S_2$ $[M + H]^+$, 251.0195; found, 251.0209.

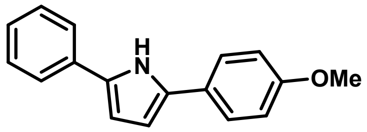

**2-(4-Methoxyphenyl)-5-phenyl-1*H*-pyrrole (4)**[13]

$NH_4OAc$ (34 mg, 0.44 mmol) was added to a solution of **3a** (0.2 mmol) in MeOH (5 mL) at rt. The reaction mixture was refluxed for 12 h. Then, the reaction mixture was cooled down to rt. The resulting mixture was concentrated and the residue was taken up in ethyl acetate. The organic layer was washed with brine, dried over $Na_2SO_4$ and concentrated. Purification of the crude product by column chromatography (silica gel; petroleum ether/ethyl acetate 16:1) afforded **4** in 86% yield (43 mg).

White solid; mp 162–163 °C; $^1$H NMR ($CDCl_3$, 400 MHz): $\delta_H$ 8.47 (s, 1H), 7.51 (d, *J* = 8.0 Hz, 2H), 7.45 (d, *J* = 8.4 Hz, 2H), 7.37 (t, *J* = 7.8 Hz 2H), 7.24–7.18 (m, 1H), 6.93 (d, *J* = 8.4 Hz, 2H), 6.56 (m, 1H), 6.46 (s, 1H), 3.82 (s, 3H); $^{13}$C NMR ($CDCl_3$, 101 MHz): $\delta_C$ 158.4, 133.2, 132.6, 132.4, 128.9, 126.1, 125.5, 125.2, 123.6, 114.4, 107.7, 106.8, 55.3; HRMS (ESI-TOF, *m/z*): calcd for $C_{17}H_{15}NO$ $[M + H]^+$, 250.1226; found, 250.1236.

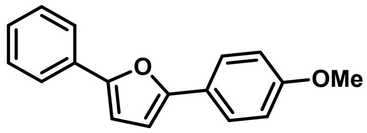

**2-(4-Methoxyphenyl)-5-phenylfuran (5)**[14]

To a dry Schlenk tube equipped with a magnetic stir bar was added 1,4-diketone **3a** (0.2 mmol, 53 mg). Then, freshly distilled $CH_3CN$ (2.0 mL) was added with a syringe. Subsequently, TfOH (27 µL, 0.3 mmol, 45 mg) was added to the mixture, and the resulting solution was stirred at 85 $^o$C for 1 h under argon. Then, the resulting solution was cooled to room temperature, and solvent was concentrated. The residue was purified by column chromatography (silica gel; petroleum ether/ethyl acetate 20:1) to give furan **5** in 90% yield (45 mg).

White solid; mp 121–122 °C; $^1$H NMR ($CDCl_3$, 400 MHz): $\delta_H$ 7.73 (d, *J* = 7.6 Hz, 2H), 7.68 (d, *J* = 8.8 Hz, 2H), 7.42–7.38 (m, 2H), 7.27–7.24 (m, 1H), 6.95 (d, *J* = 8.8 Hz, 2H), 6.72 (d, *J* = 3.6 Hz, 1H), 6.61 (d, *J* = 3.6 Hz, 1H), 3.85 (s, 3H); $^{13}$C NMR ($CDCl_3$, 101 MHz): $\delta_C$ 159.0, 153.4, 152.6, 130.9, 128.7, 127.1, 125.2, 123.9, 123.5, 114.1, 107.2, 105.6, 55.3; HRMS (ESI-TOF, *m/z*): calcd for $C_{17}H_{15}O_2$ $[M + H]^+$, 251.1067; found, 251.1078.

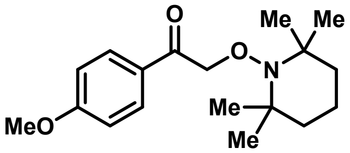

**1-(4-Methoxyphenyl)-2-((2,2,6,6-tetramethylpiperidin-1-yl)oxy)ethan-1-one (6)**[15]

To a 10 mL Schlenk tube equipped with a magnetic stir bar was added ((1-(4-methoxyphenyl)vinyl)oxy)trimethylsilane **1a** (144.5 mg, 0.65 mmol), trimethyl((1-phenylvinyl)oxy)silane **2a** (96 mg, 0.5 mmol), $CH_3CN$ (2.0 mL), PhBr (157 mg, 1.0 mmol), TEMPO (165.25 mg,1 mmol) and AgF (13 mg, 0.1 mmol). The reaction mixture was stirred at 25 °C for about 8 h. The resulting mixture was concentrated and the residue was taken up in ethyl acetate. The organic layer was washed with brine, dried over $Na_2SO_4$ and concentrated. Purification of the crude product by column chromatography (silica gel; petroleum ether/ethyl acetate 20:1) afforded **6** in 50% yield (76 mg).

White solid; mp 115–116 °C; $^1$H NMR ($CDCl_3$, 400 MHz): $\delta_H$ 7.94 (d, $J$ = 8.8 Hz, 2H), 6.94 (d, $J$ = 8.4 Hz, 2H), 5.05 (s, 2H), 3.87 (s, 3H), 1.68–1.45 (m, 6H), 1.17 (s, 12H); $^{13}$C NMR ($CDCl_3$, 101 MHz): $\delta_C$ 194.4, 163.5, 130.3, 128.5, 113.7, 81.2, 60.1, 55.4, 39.7, 32.8, 20.2, 17.0; HRMS (ESI-TOF, $m/z$): calcd for $C_{18}H_{28}NO_3$ $[M + H]^+$, 306.2064; found, 306.2071.

## V. NMR spectra

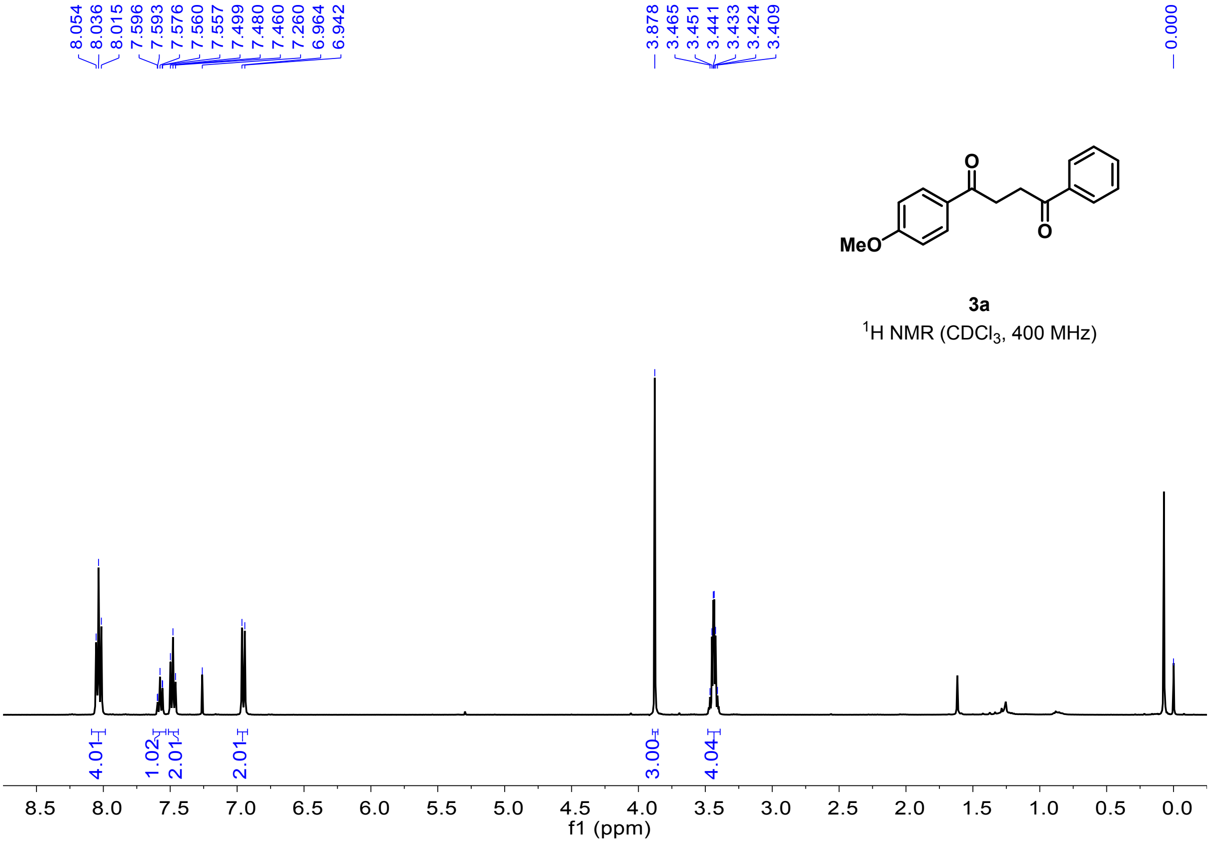


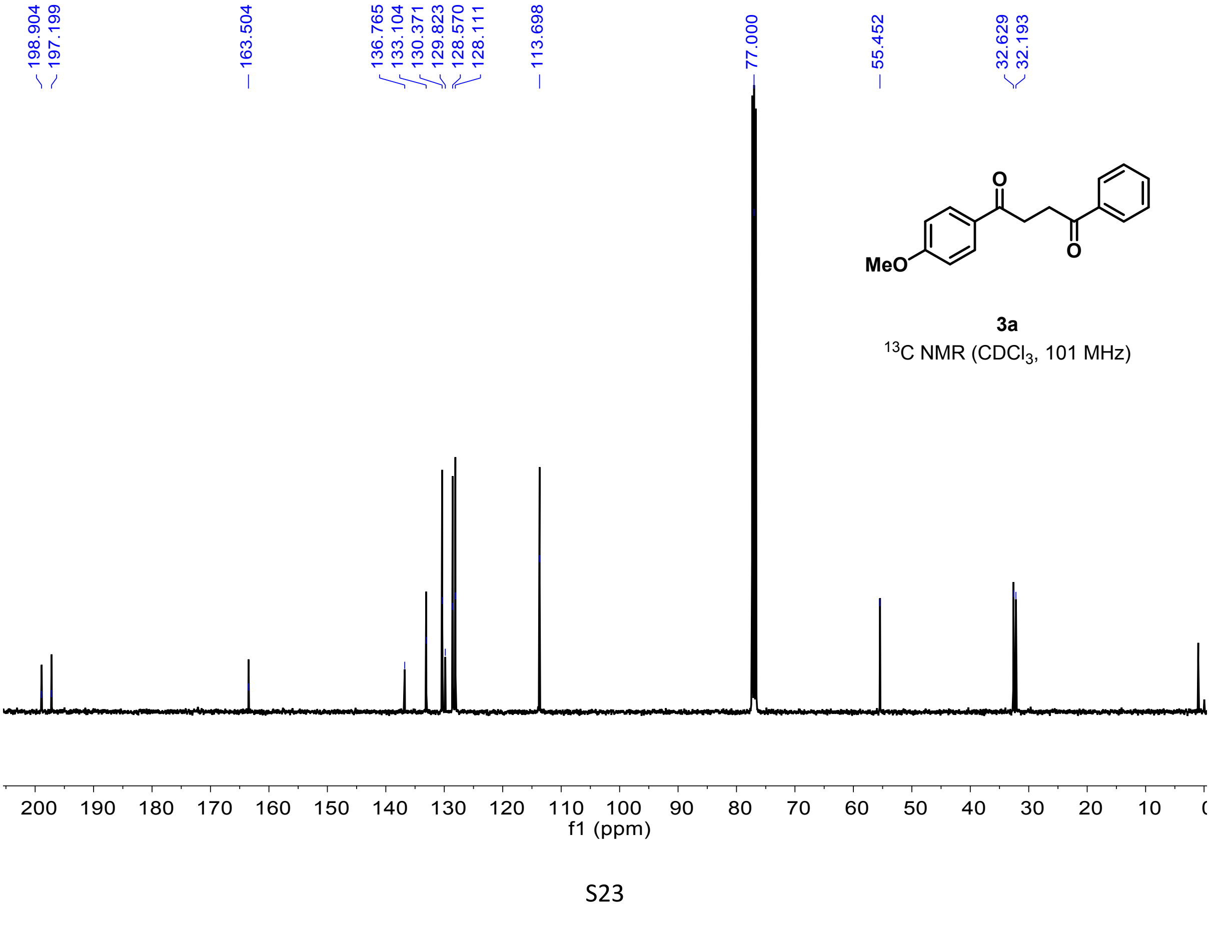

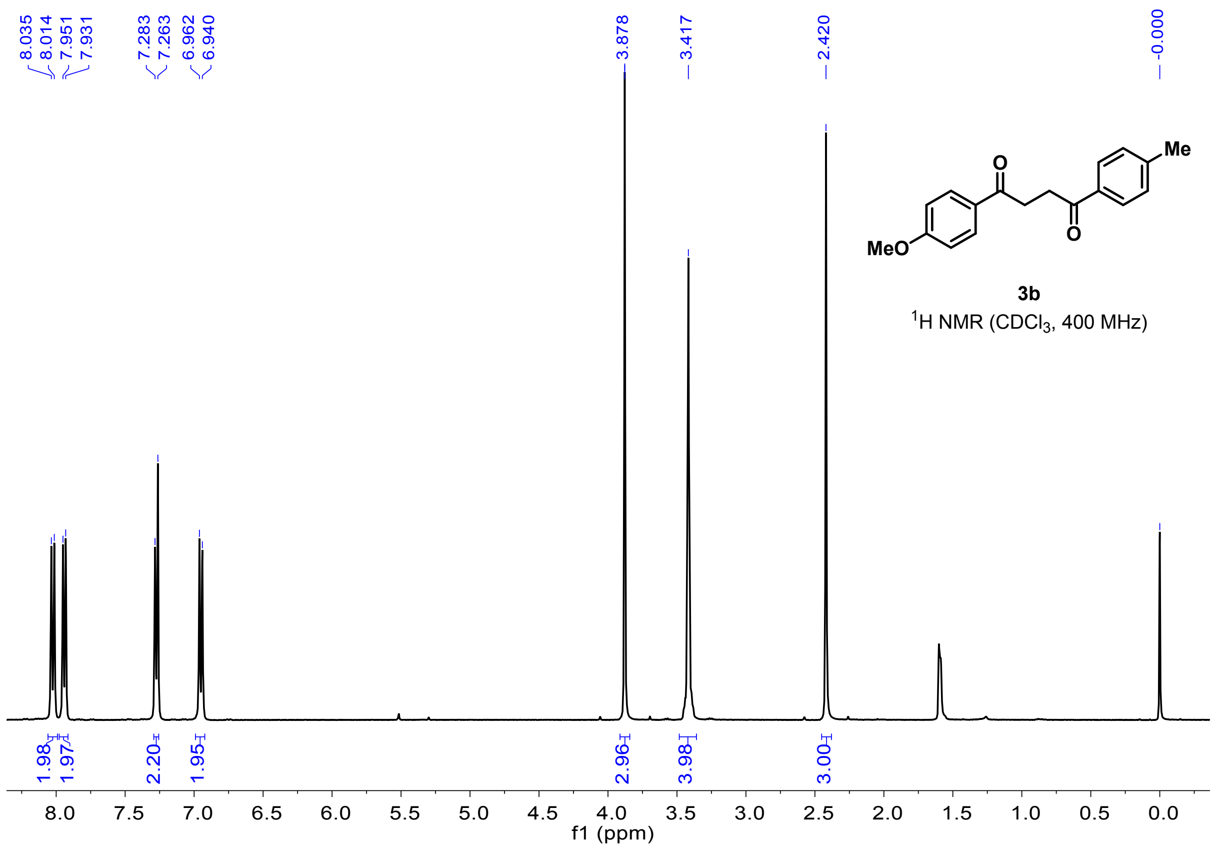
8.035
8.014
7.951
7.931
7.283
7.263
6.962
6.940
3.878
3.417
2.420
-0.000
O
Me
MeO
O
3b
1H NMR (CDCl3, 400 MHz)
1.98
1.97
2.20
1.95
2.96
3.98
3.00
8.0 7.5 7.0 6.5 6.0 5.5 5.0 4.5 4.0 3.5 3.0 2.5 2.0 1.5 1.0 0.5 0.0
f1 (ppm)

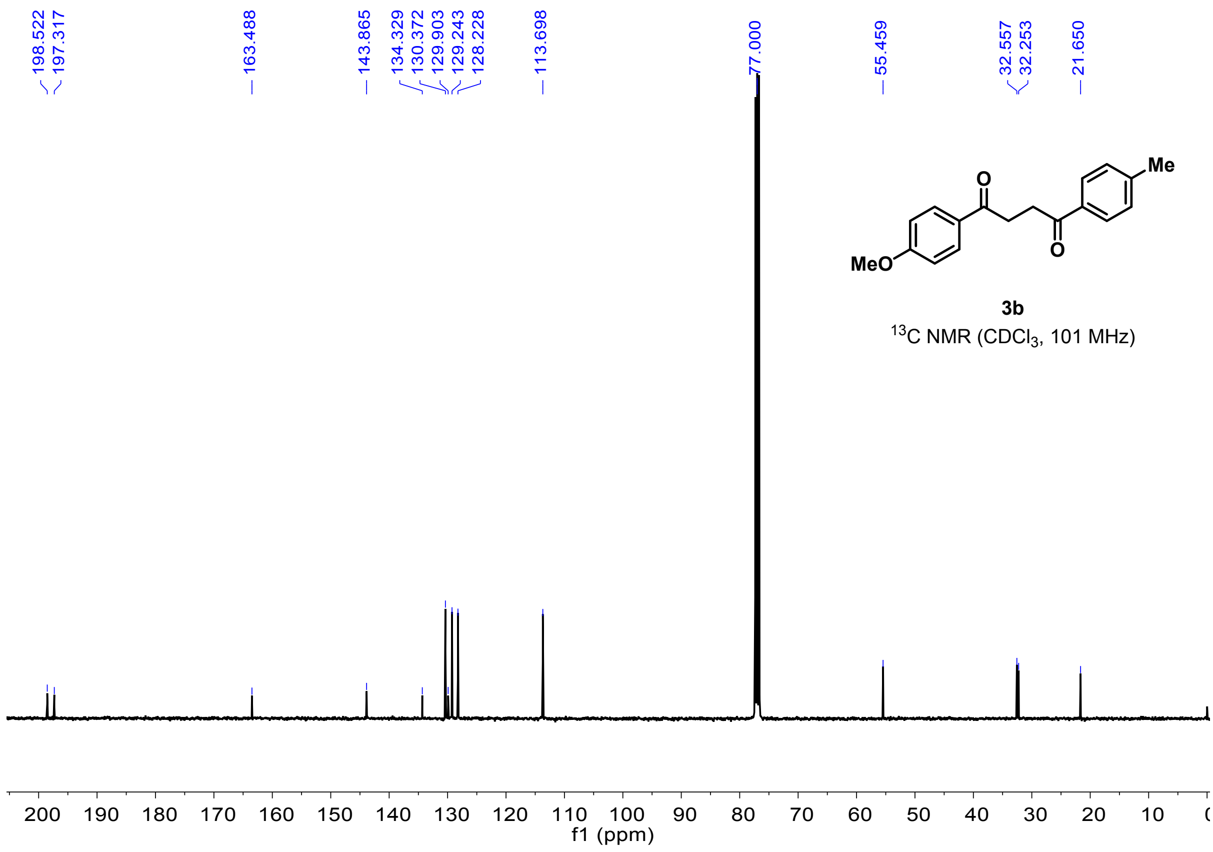
198.522
197.317
163.488
143.865
134.329
130.372
129.903
129.243
128.228
113.698
77.000
55.459
32.557
32.253
21.650
O
Me
MeO
O
3b
13C NMR (CDCl3, 101 MHz)
200 190 180 170 160 150 140 130 120 110 100 90 80 70 60 50 40 30 20 10
f1 (ppm)

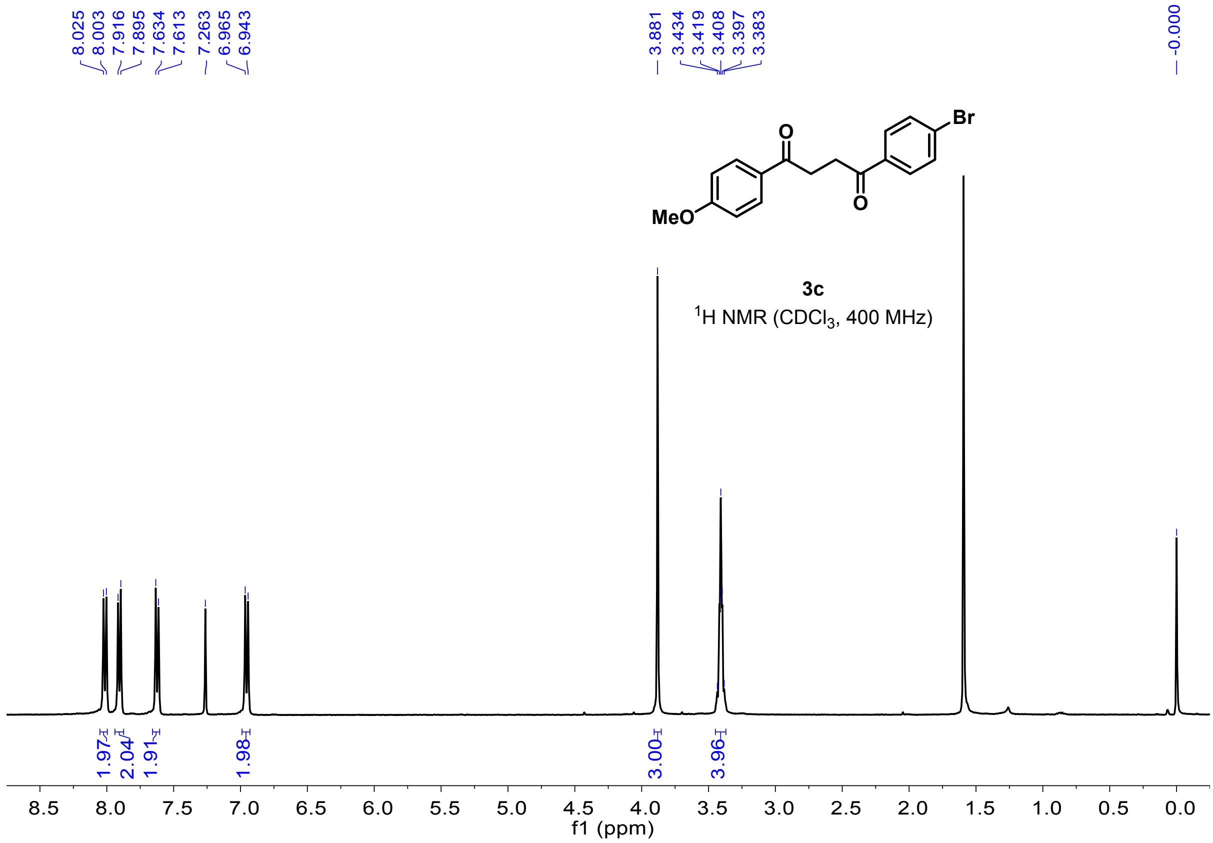
8.025
8.003
7.916
7.895
7.634
7.613
7.263
6.965
6.943
3.881
3.434
3.419
3.408
3.397
3.383
-0.000
Br
O
O
MeO
3c
1H NMR (CDCl3, 400 MHz)
1.97
2.04
1.91
1.98
3.00
3.96
f1 (ppm)

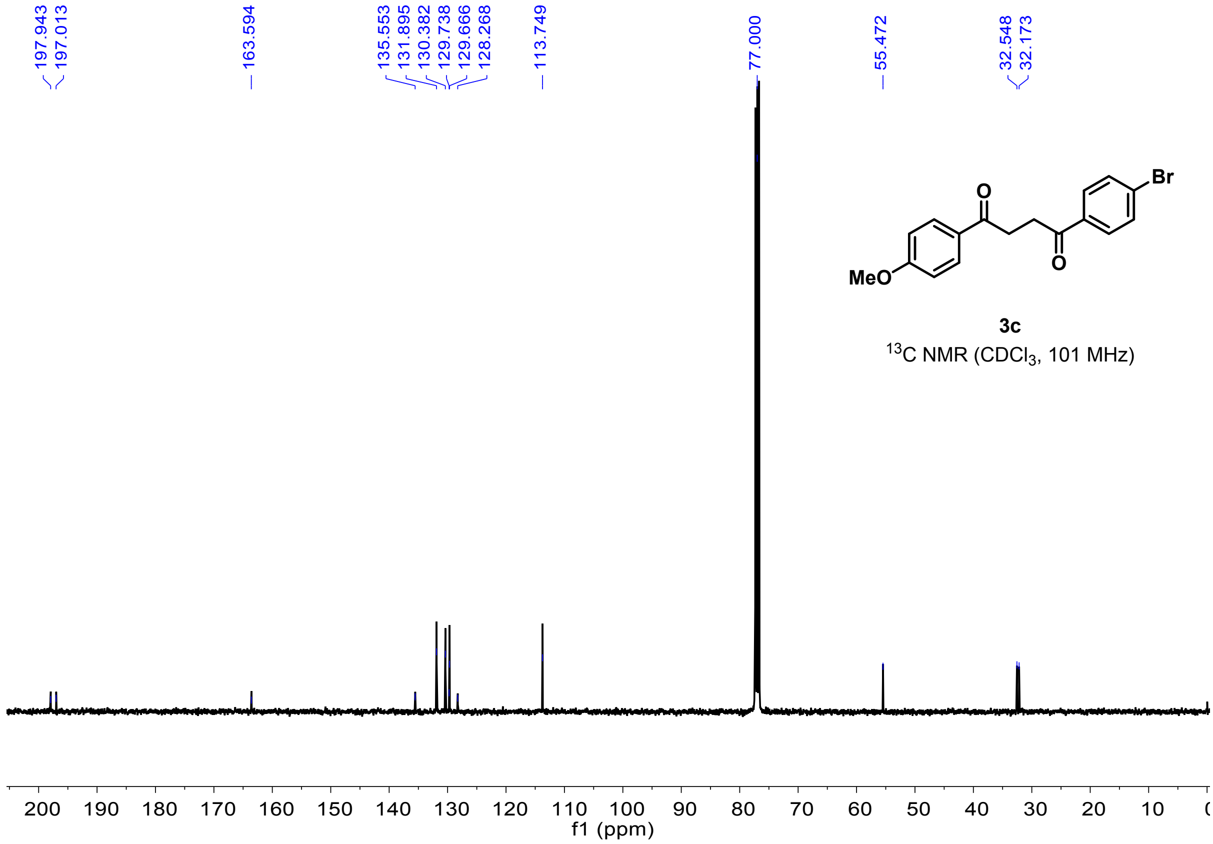
197.943
197.013
163.594
135.553
131.895
130.382
129.738
129.666
128.268
113.749
77.000
55.472
32.548
32.173
Br
O
O
MeO
3c
13C NMR (CDCl3, 101 MHz)
f1 (ppm)

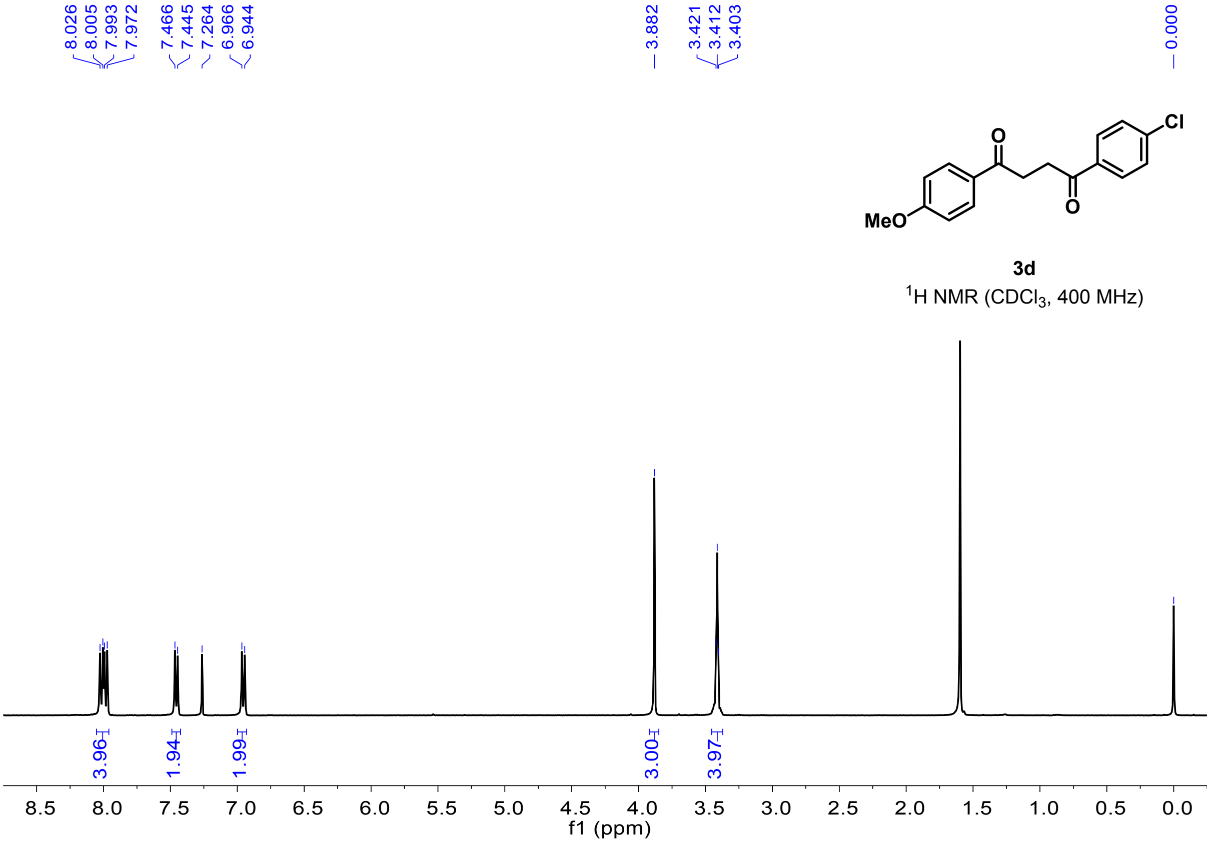
8.026
8.005
7.993
7.972
7.466
7.445
7.264
6.966
6.944
3.882
3.421
3.412
3.403
0.000
O
Cl
MeO
O
3d
$^{1}$H NMR ($CDCl_3$, 400 MHz)
3.96
1.94
1.99
3.00
3.97
8.5 8.0 7.5 7.0 6.5 6.0 5.5 5.0 4.5 4.0 3.5 3.0 2.5 2.0 1.5 1.0 0.5 0.0
f1 (ppm)

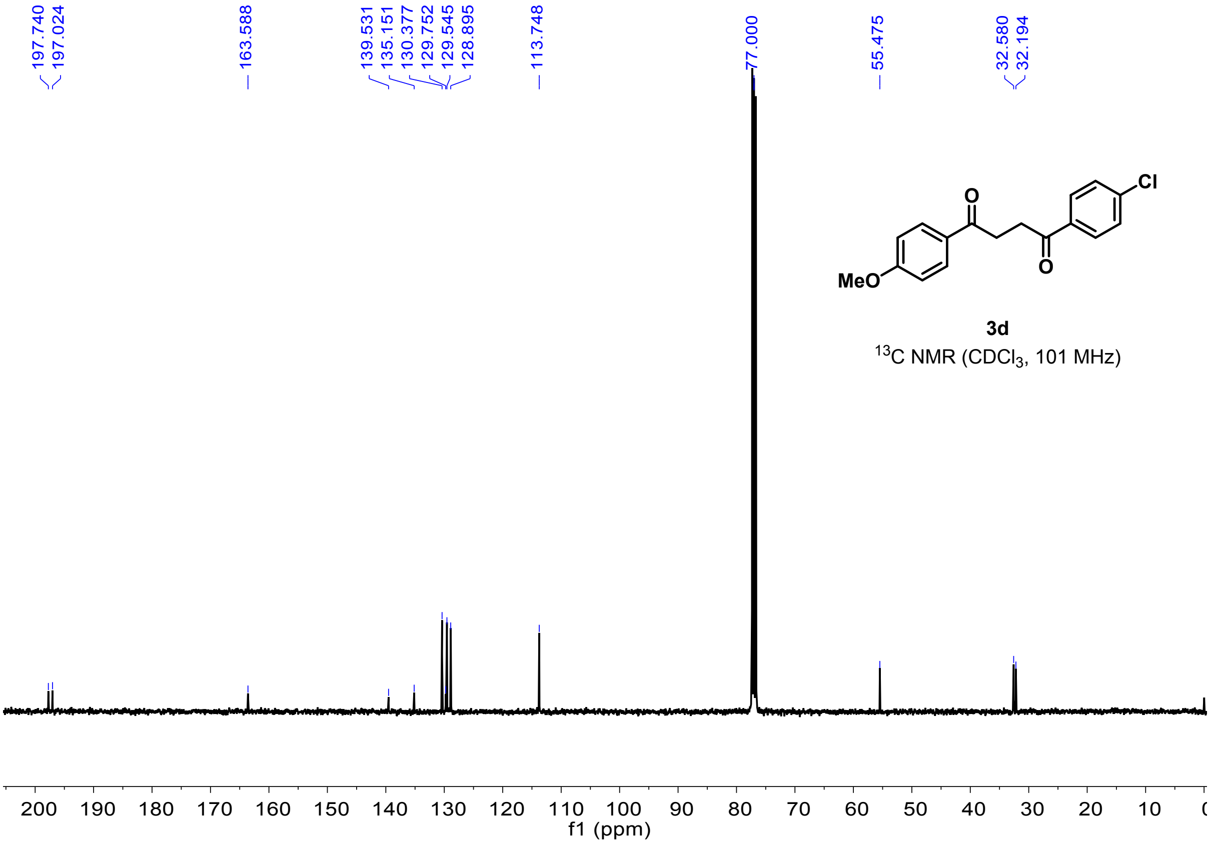
197.740
197.024
163.588
139.531
135.151
130.377
129.752
129.545
128.895
113.748
77.000
55.475
32.580
32.194
O
Cl
MeO
O
3d
$^{13}$C NMR ($CDCl_3$, 101 MHz)
200 190 180 170 160 150 140 130 120 110 100 90 80 70 60 50 40 30 20 10
f1 (ppm)

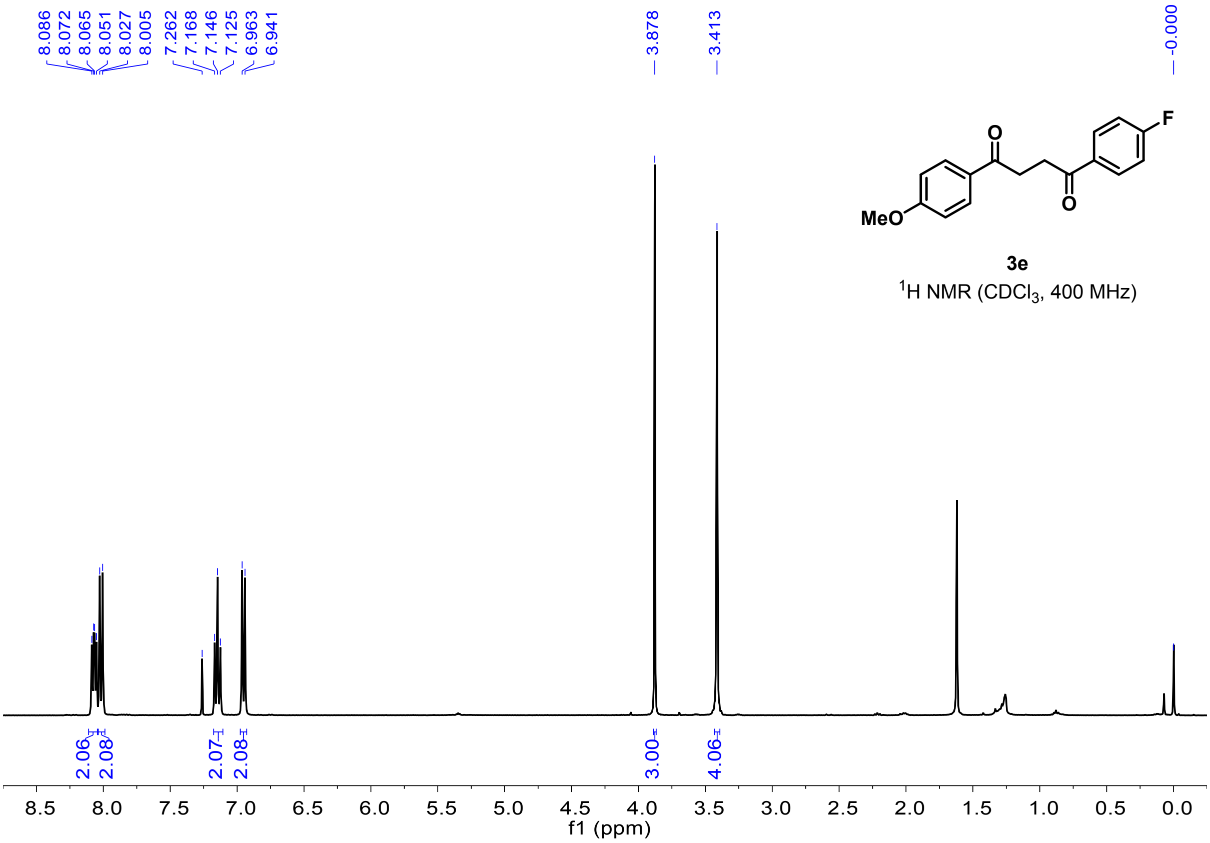
8.086
8.072
8.065
8.051
8.027
8.005
7.262
7.168
7.146
7.125
6.963
6.941
3.878
3.413
-0.000
MeO
O
O
F
3e
$^1$H NMR ($CDCl_3$, 400 MHz)
2.06
2.08
2.07
2.08
3.00
4.06
8.5 8.0 7.5 7.0 6.5 6.0 5.5 5.0 4.5 4.0 3.5 3.0 2.5 2.0 1.5 1.0 0.5 0.0
f1 (ppm)

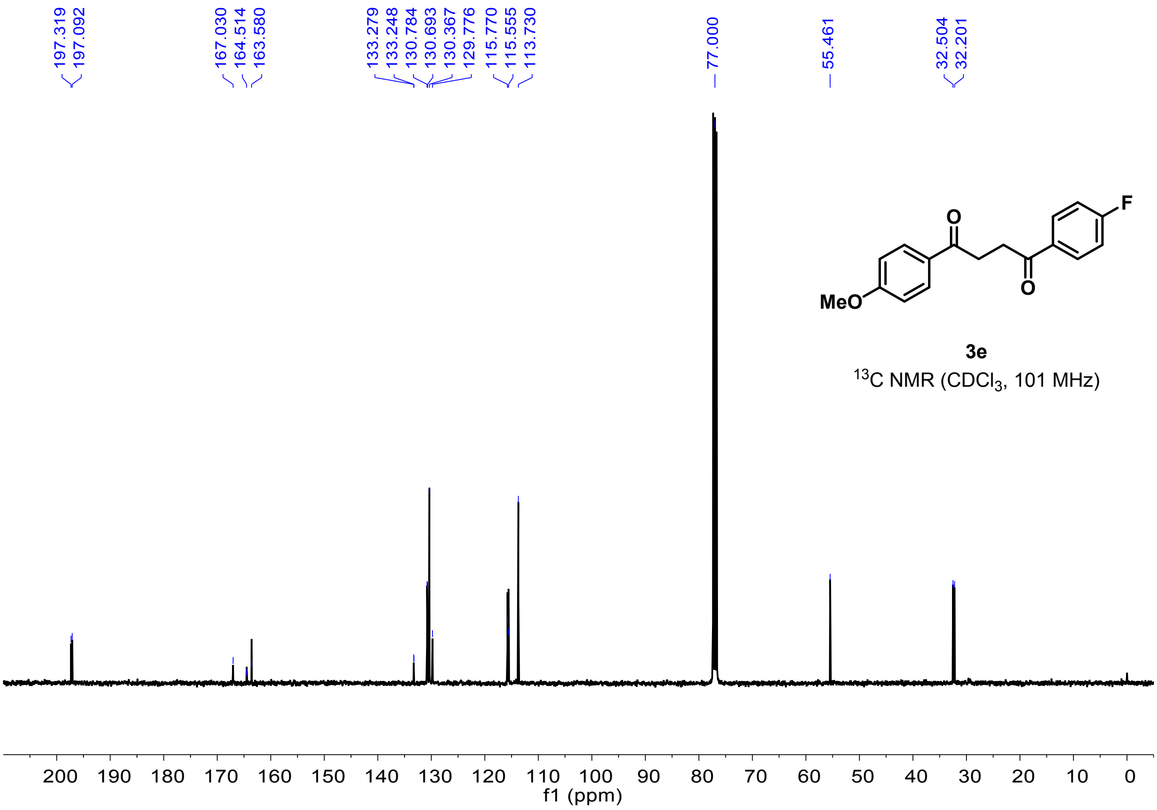
197.319
197.092
167.030
164.514
163.580
133.279
133.248
130.784
130.693
130.367
129.776
115.770
115.555
113.730
77.000
55.461
32.504
32.201
MeO
O
O
F
3e
$^{13}$C NMR ($CDCl_3$, 101 MHz)
200 190 180 170 160 150 140 130 120 110 100 90 80 70 60 50 40 30 20 10 0
f1 (ppm)

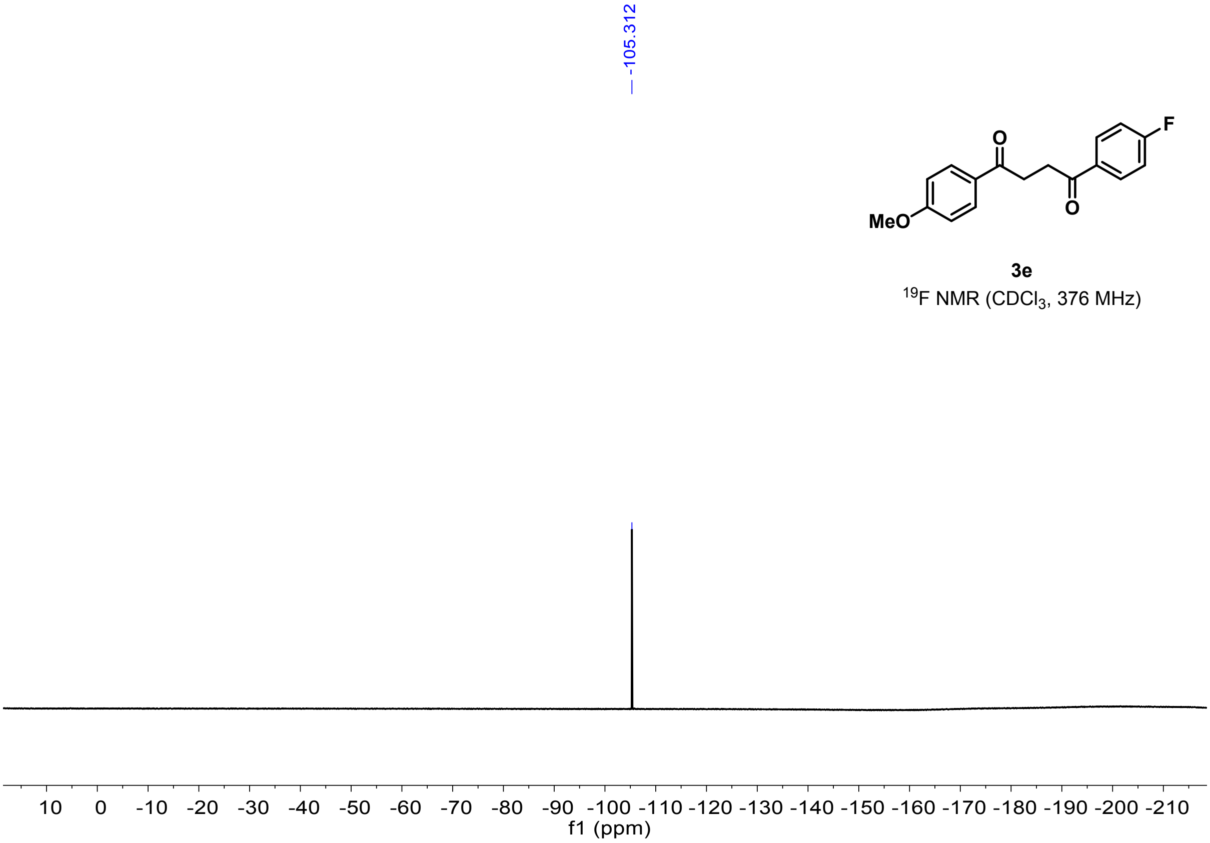
-105.312
F
O
O
MeO
3e
19F NMR (CDCl3, 376 MHz)
10 0 -10 -20 -30 -40 -50 -60 -70 -80 -90 -100 -110 -120 -130 -140 -150 -160 -170 -180 -190 -200 -210
f1 (ppm)

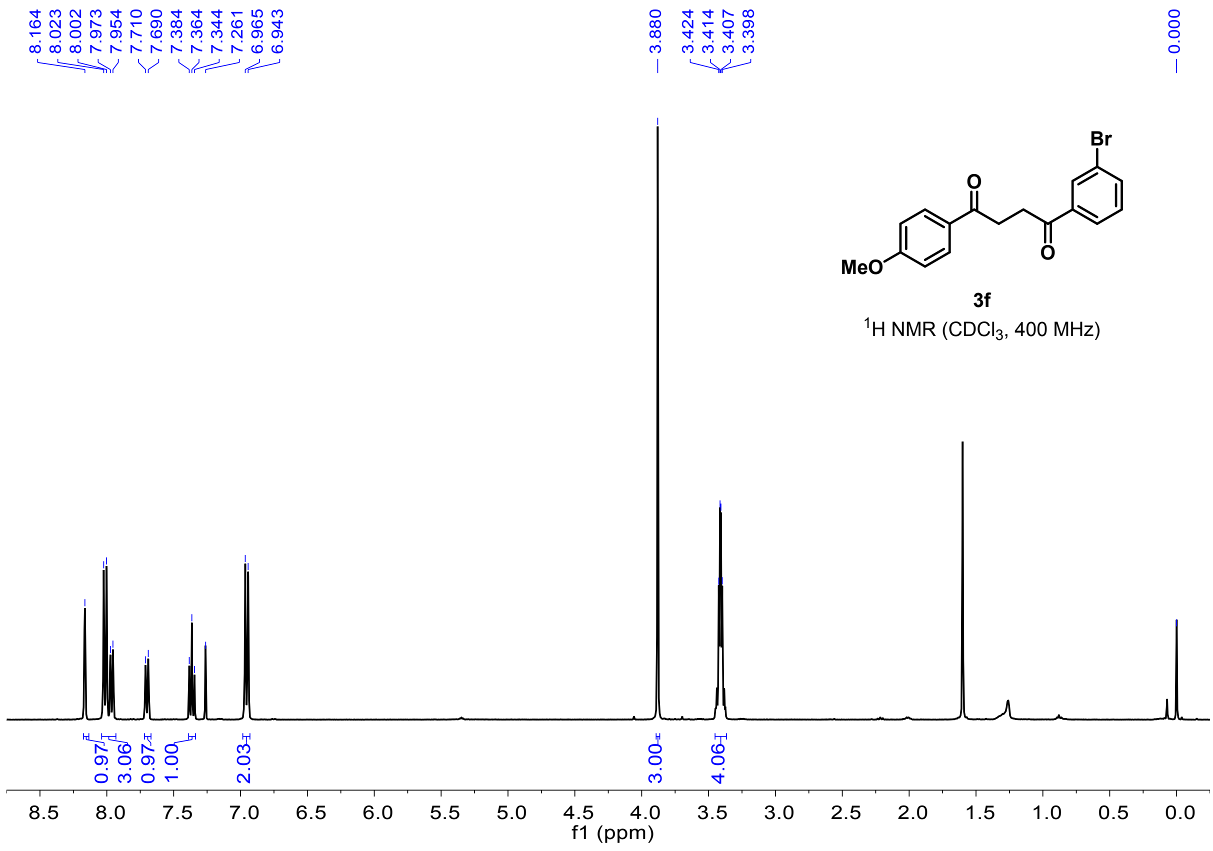

8.164
8.023
8.002
7.973
7.954
7.710
7.690
7.384
7.364
7.344
7.261
6.965
6.943
3.880
3.424
3.414
3.407
3.398
0.000
Br
O
O
MeO
3f
$^{1}$H NMR (CDCl$_3$, 400 MHz)
0.97
3.06
0.97
1.00
2.03
3.00
4.06
8.5 8.0 7.5 7.0 6.5 6.0 5.5 5.0 4.5 4.0 3.5 3.0 2.5 2.0 1.5 1.0 0.5 0.0
f1 (ppm)


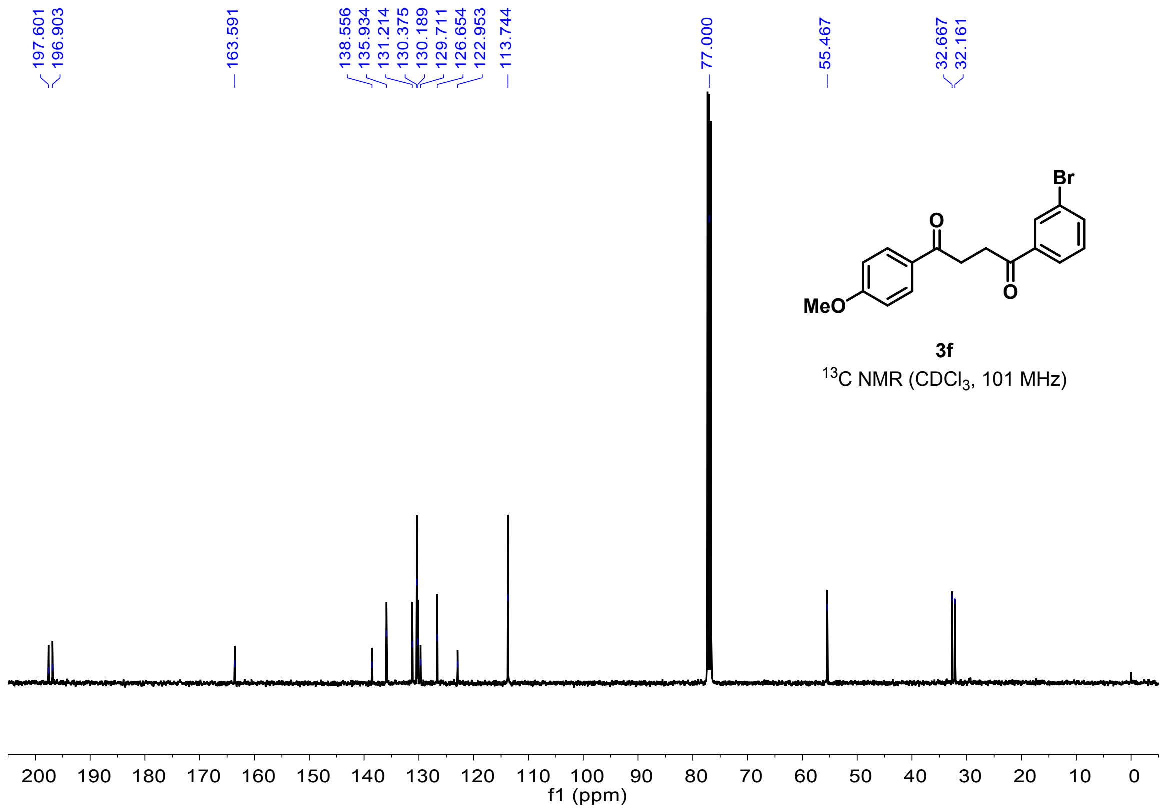

197.601
196.903
163.591
138.556
135.934
131.214
130.375
130.189
129.711
126.654
122.953
113.744
77.000
55.467
32.667
32.161
Br
O
O
MeO
3f
$^{13}$C NMR (CDCl$_3$, 101 MHz)
200 190 180 170 160 150 140 130 120 110 100 90 80 70 60 50 40 30 20 10 0
f1 (ppm)

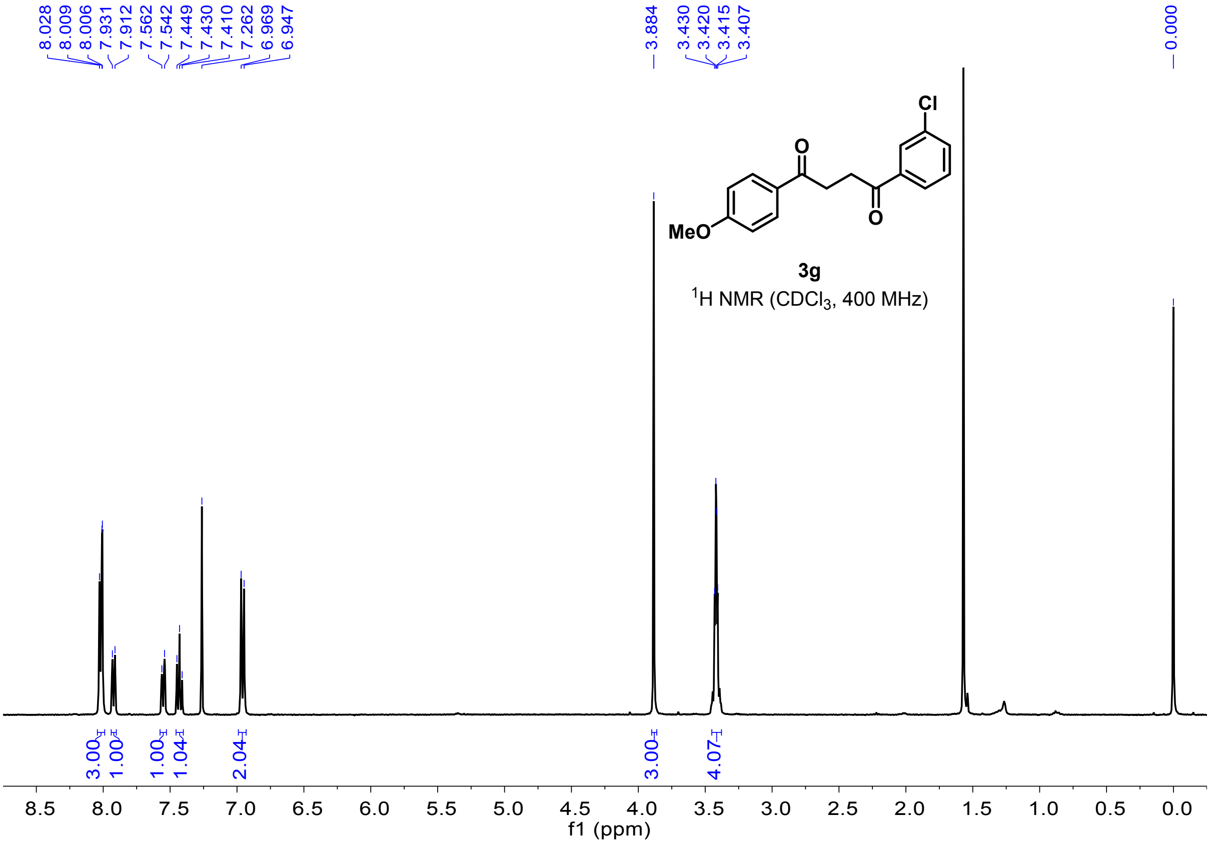
8.028
8.009
8.006
7.931
7.912
7.562
7.542
7.449
7.430
7.410
7.262
6.969
6.947
3.884
3.430
3.420
3.415
3.407
0.000
Cl
O
O
MeO
3g
$^{1}$H NMR (CDCl$_3$, 400 MHz)
3.00
1.00
1.00
1.04
2.04
3.00
4.07
8.5 8.0 7.5 7.0 6.5 6.0 5.5 5.0 4.5 4.0 3.5 3.0 2.5 2.0 1.5 1.0 0.5 0.0
f1 (ppm)

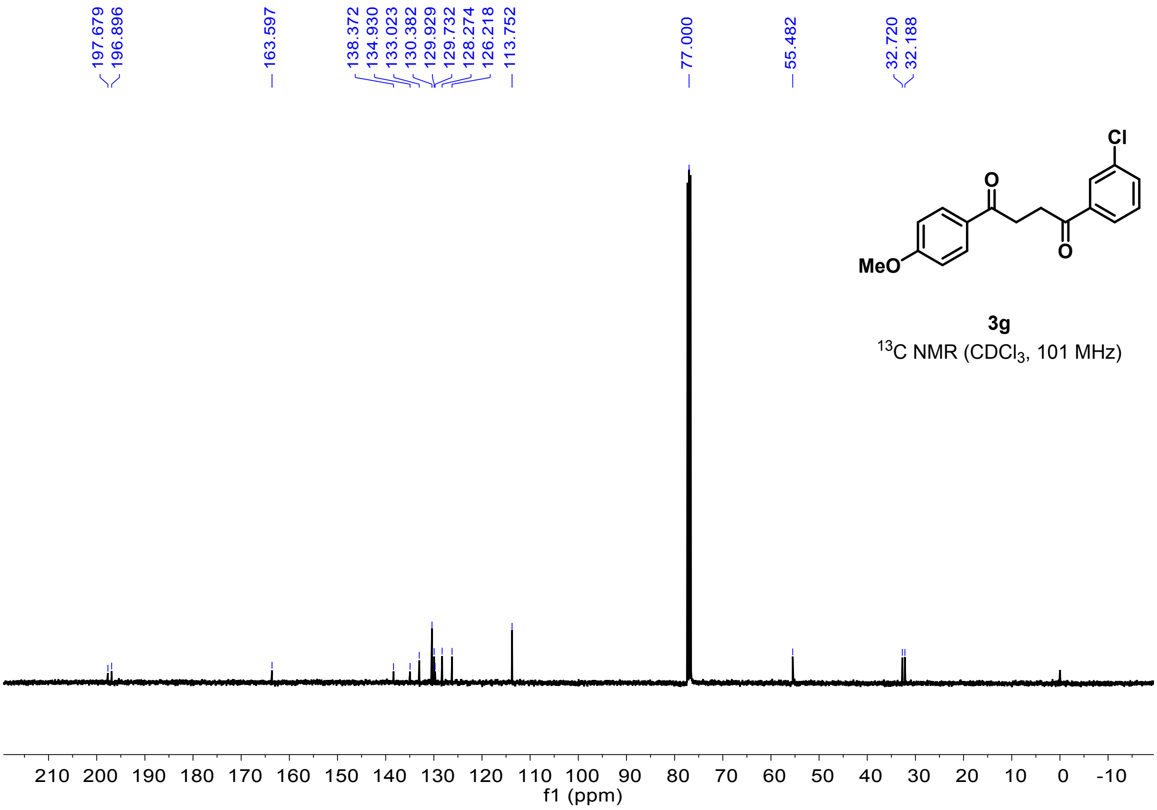
197.679
196.896
163.597
138.372
134.930
133.023
130.382
129.929
129.732
128.274
126.218
113.752
77.000
55.482
32.720
32.188
Cl
O
O
MeO
3g
$^{13}$C NMR (CDCl$_3$, 101 MHz)
210 200 190 180 170 160 150 140 130 120 110 100 90 80 70 60 50 40 30 20 10 0 -10
f1 (ppm)

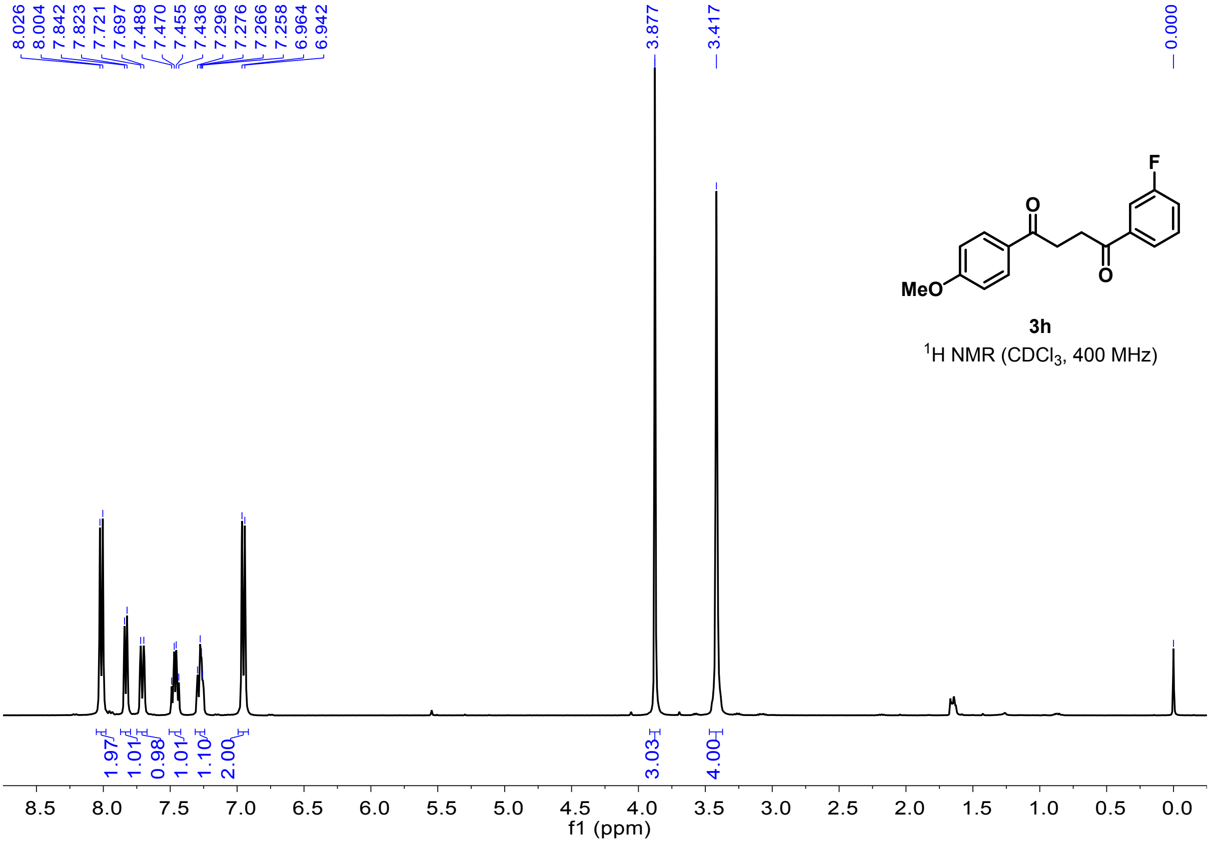
8.026
8.004
7.842
7.823
7.721
7.697
7.489
7.470
7.455
7.436
7.296
7.276
7.266
7.258
6.964
6.942
3.877
3.417
0.000
F
O
O
MeO
3h
1H NMR (CDCl3, 400 MHz)
1.97
1.01
0.98
1.01
1.10
2.00
3.03
4.00
8.5 8.0 7.5 7.0 6.5 6.0 5.5 5.0 4.5 4.0 3.5 3.0 2.5 2.0 1.5 1.0 0.5 0.0
f1 (ppm)

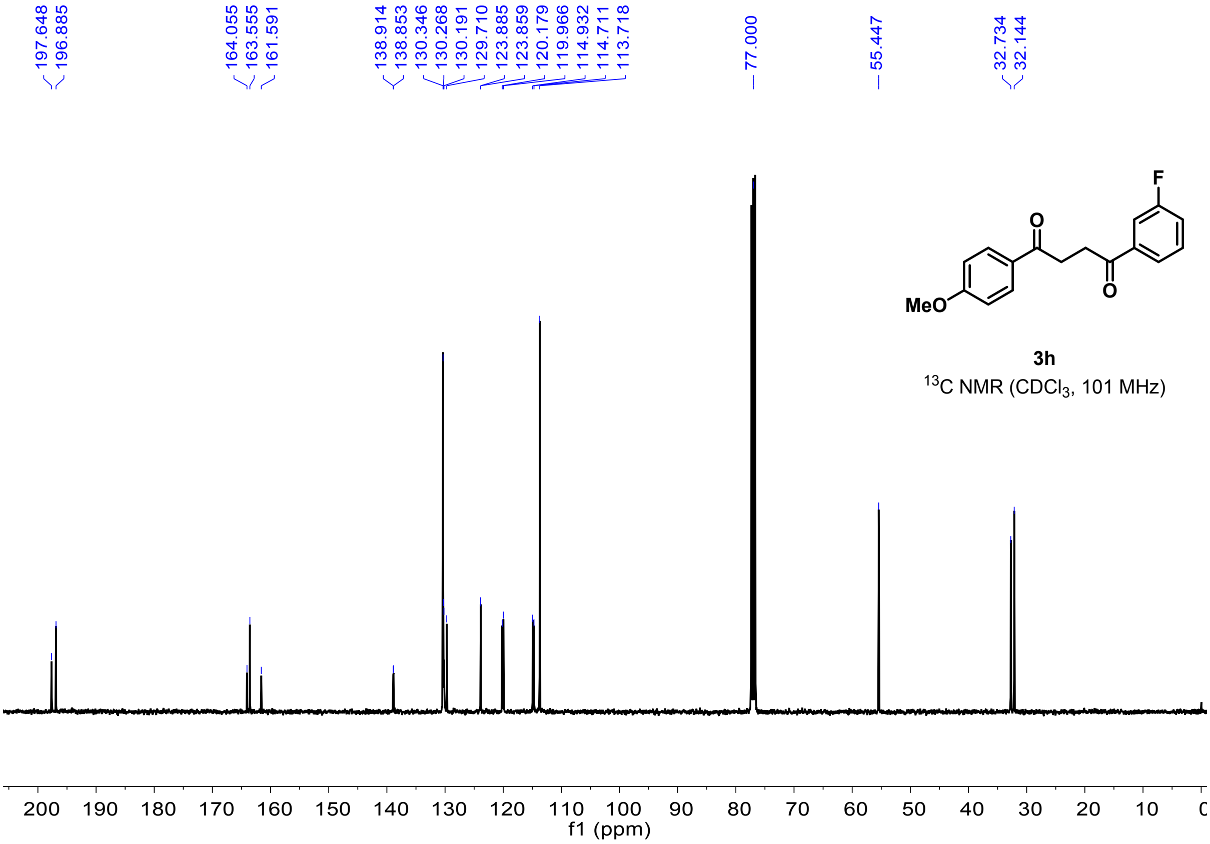
197.648
196.885
164.055
163.555
161.591
138.914
138.853
130.346
130.268
130.191
129.710
123.885
123.859
120.179
119.966
114.932
114.711
113.718
77.000
55.447
32.734
32.144
F
O
O
MeO
3h
13C NMR (CDCl3, 101 MHz)
200 190 180 170 160 150 140 130 120 110 100 90 80 70 60 50 40 30 20 10 0
f1 (ppm)

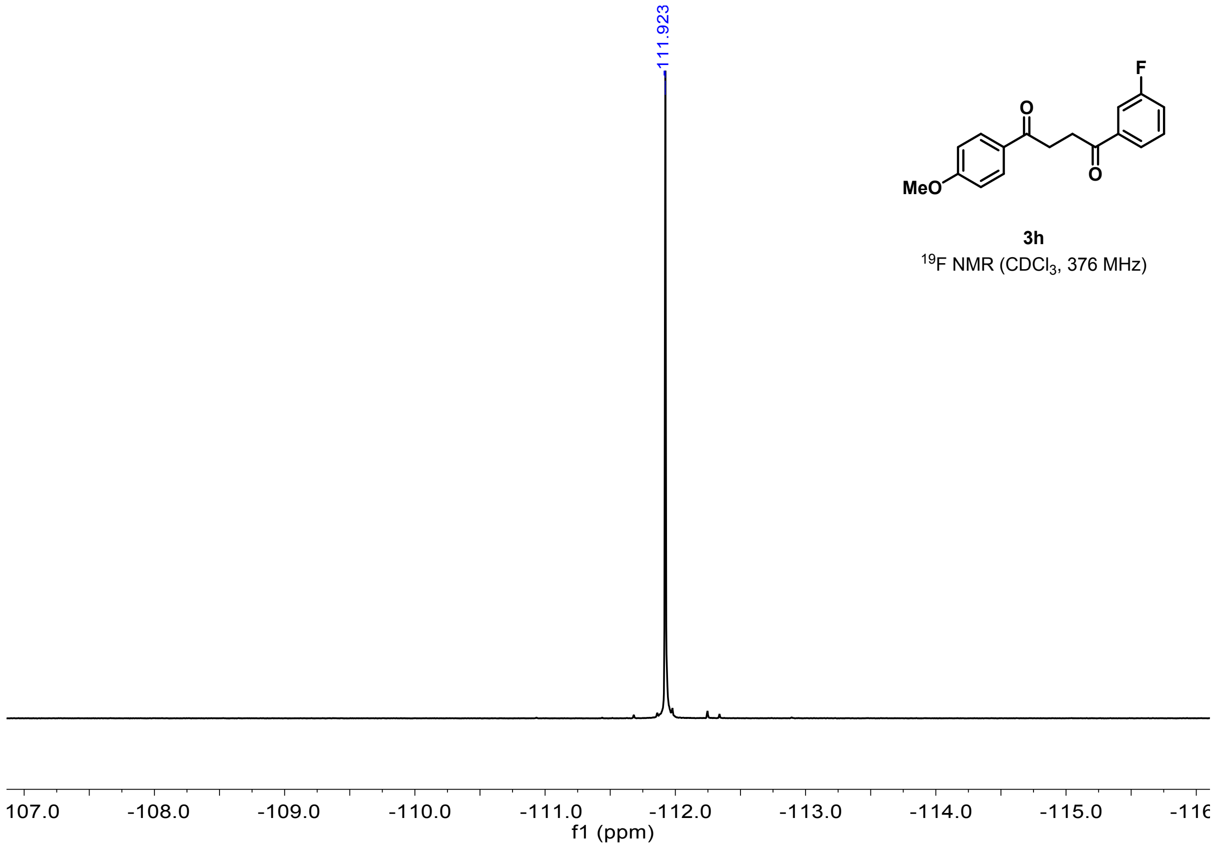
-111.923
F
O
O
MeO
3h
19F NMR (CDCl3, 376 MHz)
107.0
-108.0
-109.0
-110.0
-111.0
-112.0
-113.0
-114.0
-115.0
f1 (ppm)

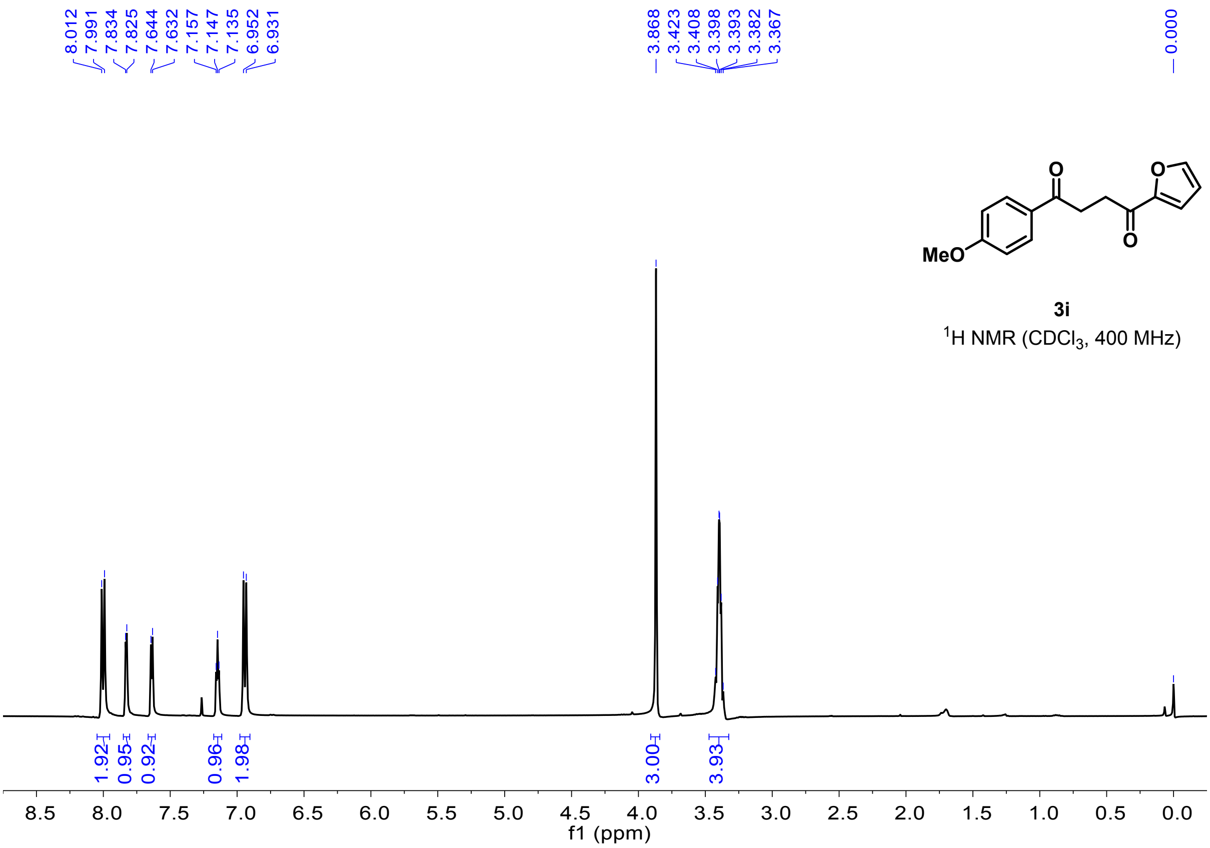
8.012
7.991
7.834
7.825
7.644
7.632
7.157
7.147
7.135
6.952
6.931
3.868
3.423
3.408
3.398
3.393
3.382
3.367
0.000
MeO
O
O
O
3i
$^{1}$H NMR (CDCl$_3$, 400 MHz)
1.92
0.95
0.92
0.96
1.98
3.00
3.93
8.5 8.0 7.5 7.0 6.5 6.0 5.5 5.0 4.5 4.0 3.5 3.0 2.5 2.0 1.5 1.0 0.5 0.0
f1 (ppm)

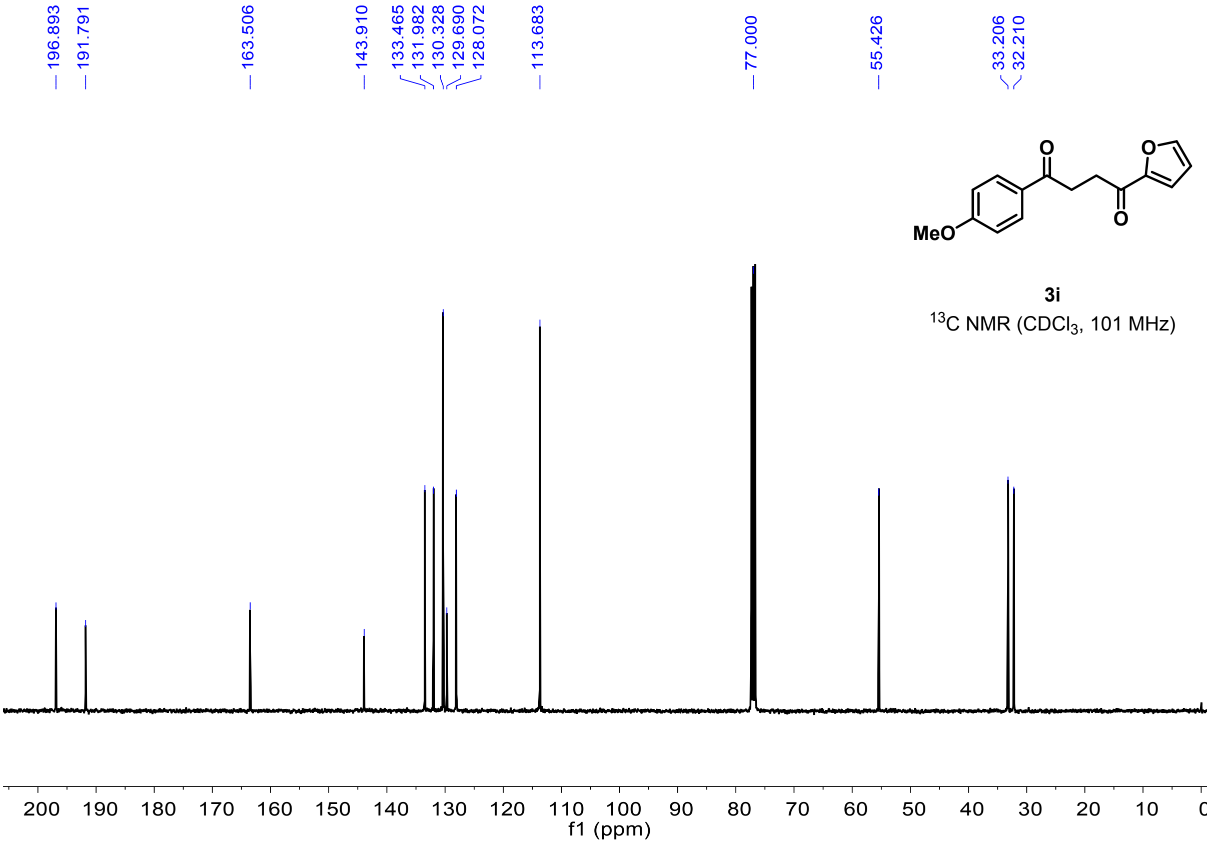
196.893
191.791
163.506
143.910
133.465
131.982
130.328
129.690
128.072
113.683
77.000
55.426
33.206
32.210
MeO
O
O
O
3i
$^{13}$C NMR (CDCl$_3$, 101 MHz)
200 190 180 170 160 150 140 130 120 110 100 90 80 70 60 50 40 30 20 10 0
f1 (ppm)

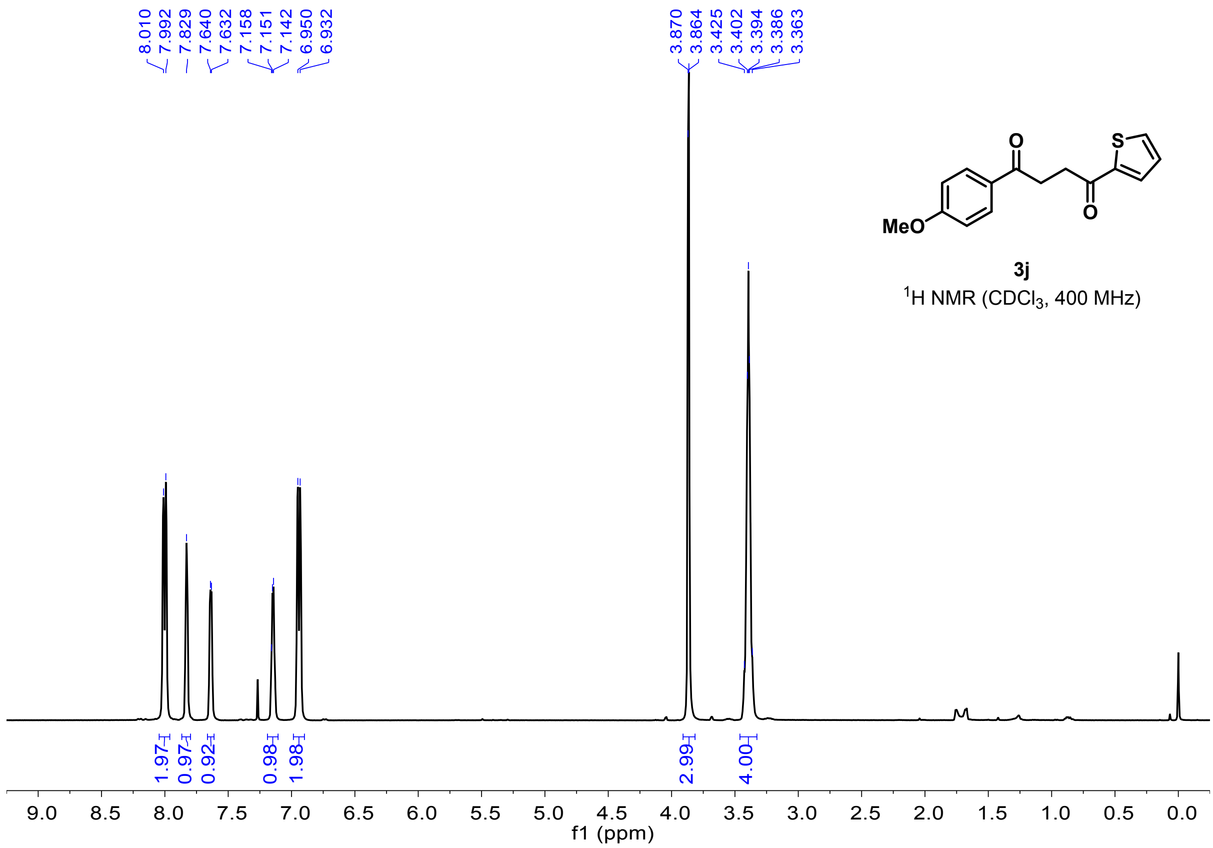
8.010
7.992
7.829
7.640
7.632
7.158
7.151
7.142
6.950
6.932
3.870
3.864
3.425
3.402
3.394
3.386
3.363
MeO
O
O
S
3j
$^1$H NMR ($CDCl_3$, 400 MHz)
1.97
0.97
0.92
0.98
1.98
2.99
4.00
9.0 8.5 8.0 7.5 7.0 6.5 6.0 5.5 5.0 4.5 4.0 3.5 3.0 2.5 2.0 1.5 1.0 0.5 0.0
f1 (ppm)

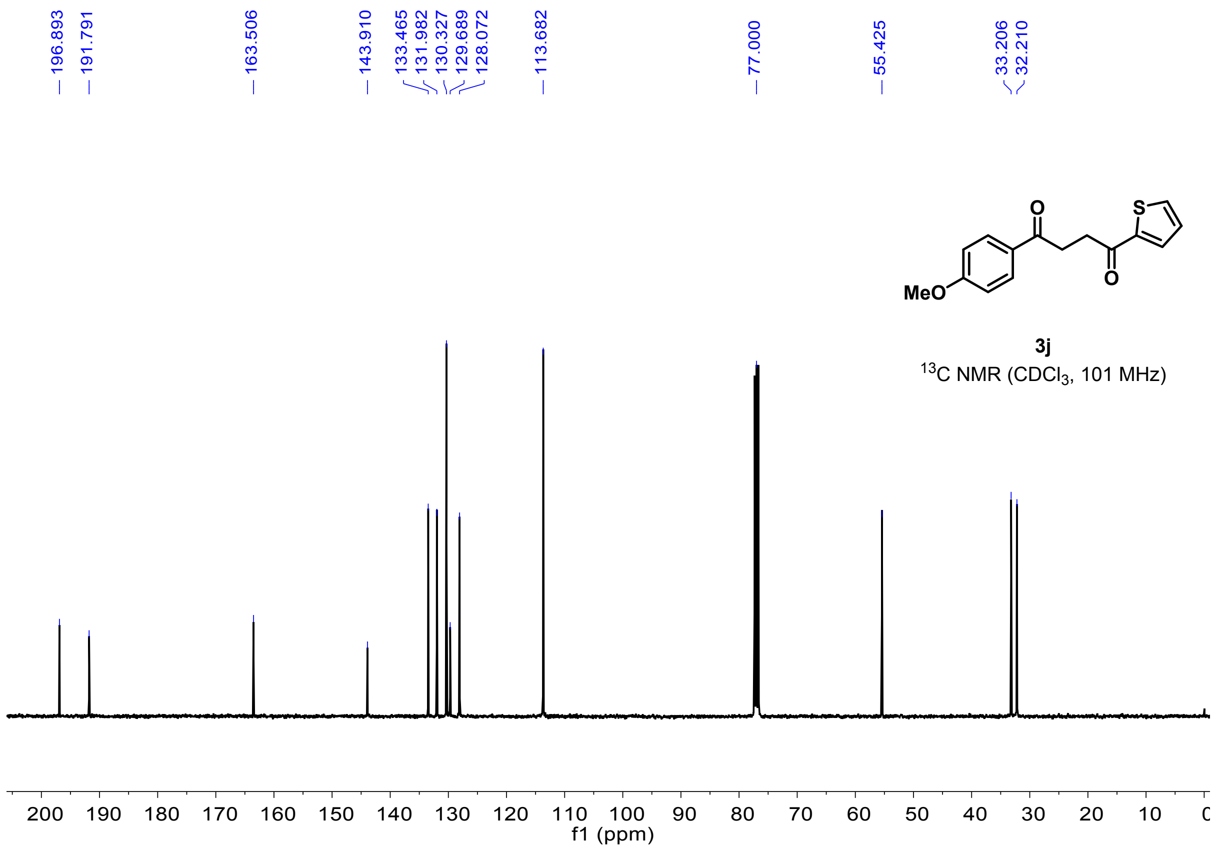
196.893
191.791
163.506
143.910
133.465
131.982
130.327
129.689
128.072
113.682
77.000
55.425
33.206
32.210
MeO
O
O
S
3j
$^{13}$C NMR ($CDCl_3$, 101 MHz)
200 190 180 170 160 150 140 130 120 110 100 90 80 70 60 50 40 30 20 10 0
f1 (ppm)

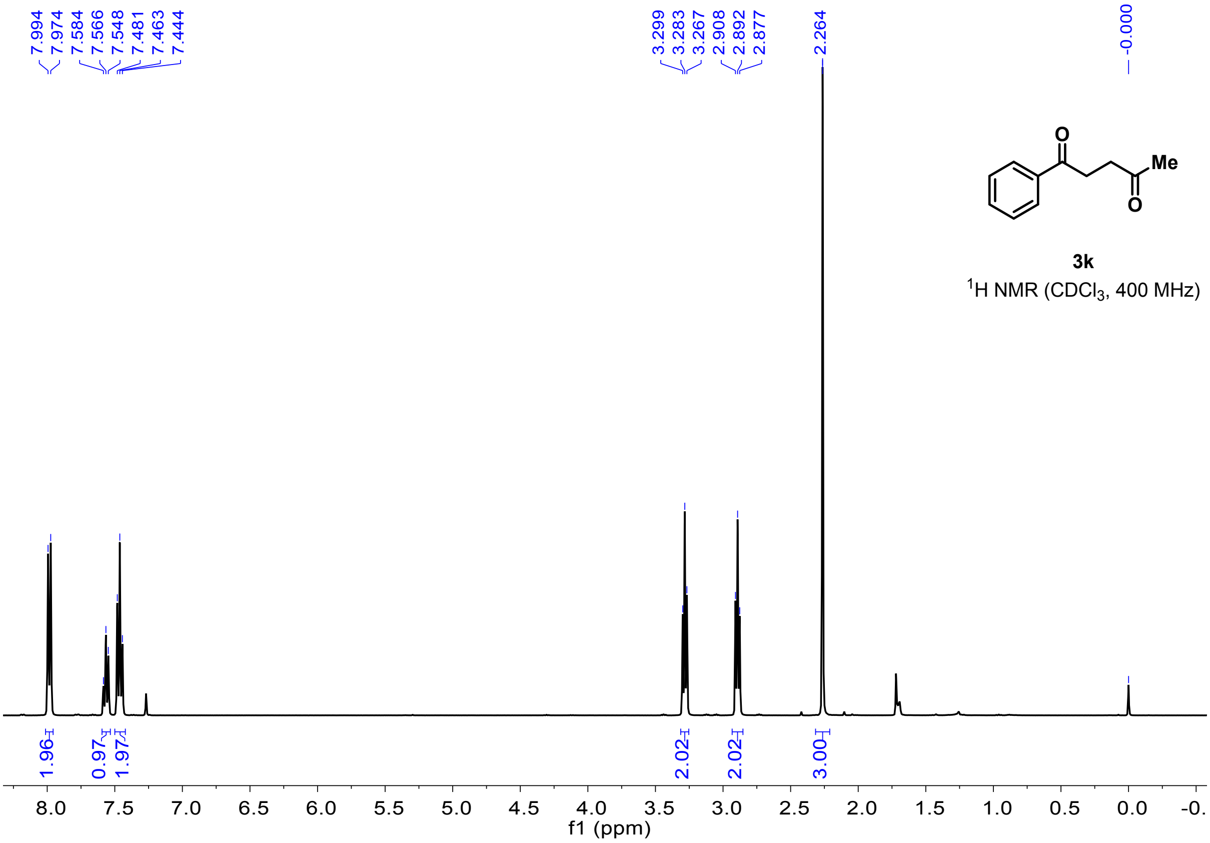

7.994
7.974
7.584
7.566
7.548
7.481
7.463
7.444
3.299
3.283
3.267
2.908
2.892
2.877
2.264
-0.000
O
Me
O
3k
1H NMR (CDCl3, 400 MHz)
1.96
0.97
1.97
2.02
2.02
3.00
f1 (ppm)


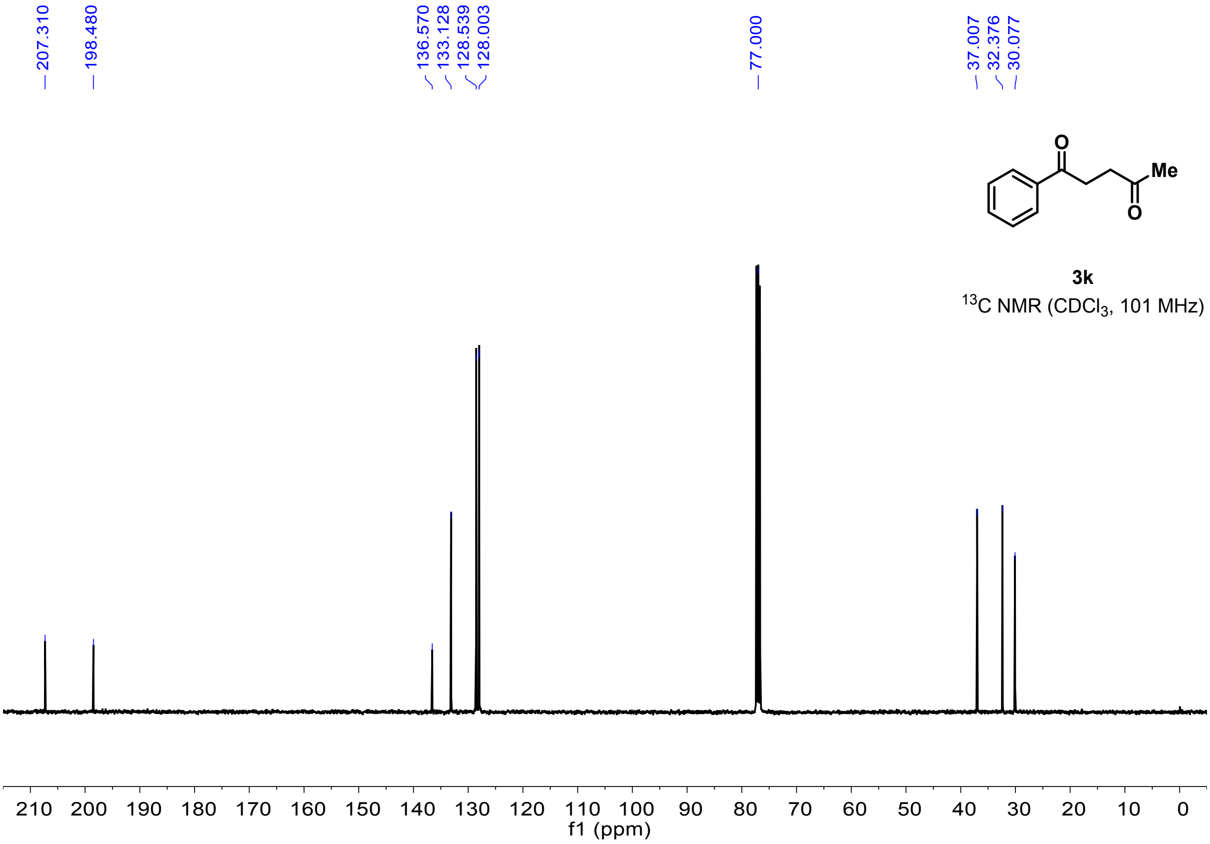

207.310
198.480
136.570
133.128
128.539
128.003
77.000
37.007
32.376
30.077
O
Me
O
3k
13C NMR (CDCl3, 101 MHz)
f1 (ppm)

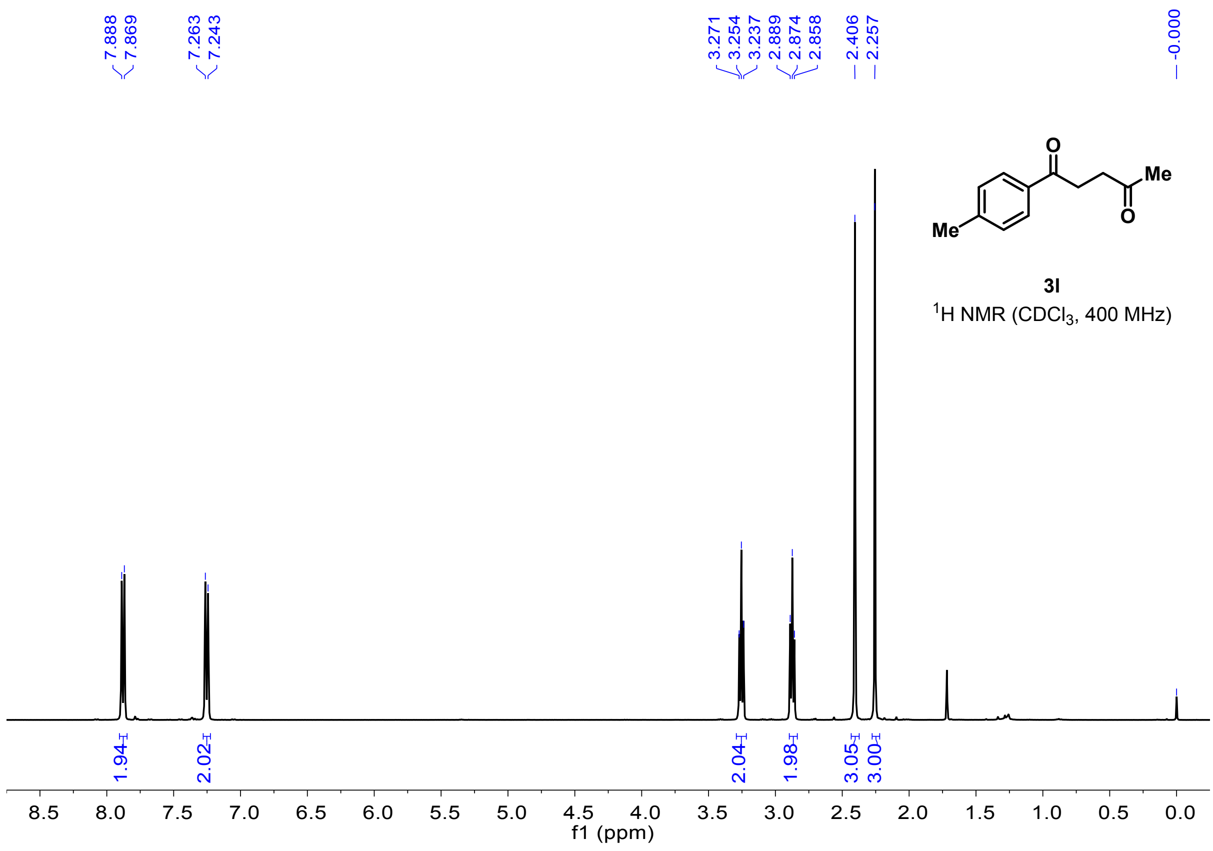
7.888
7.869
7.263
7.243
3.271
3.254
3.237
2.889
2.874
2.858
2.406
2.257
-0.000
O
Me
Me
O
3l
1H NMR (CDCl3, 400 MHz)
1.94
2.02
2.04
1.98
3.05
3.00
8.5 8.0 7.5 7.0 6.5 6.0 5.5 5.0 4.5 4.0 3.5 3.0 2.5 2.0 1.5 1.0 0.5 0.0
f1 (ppm)

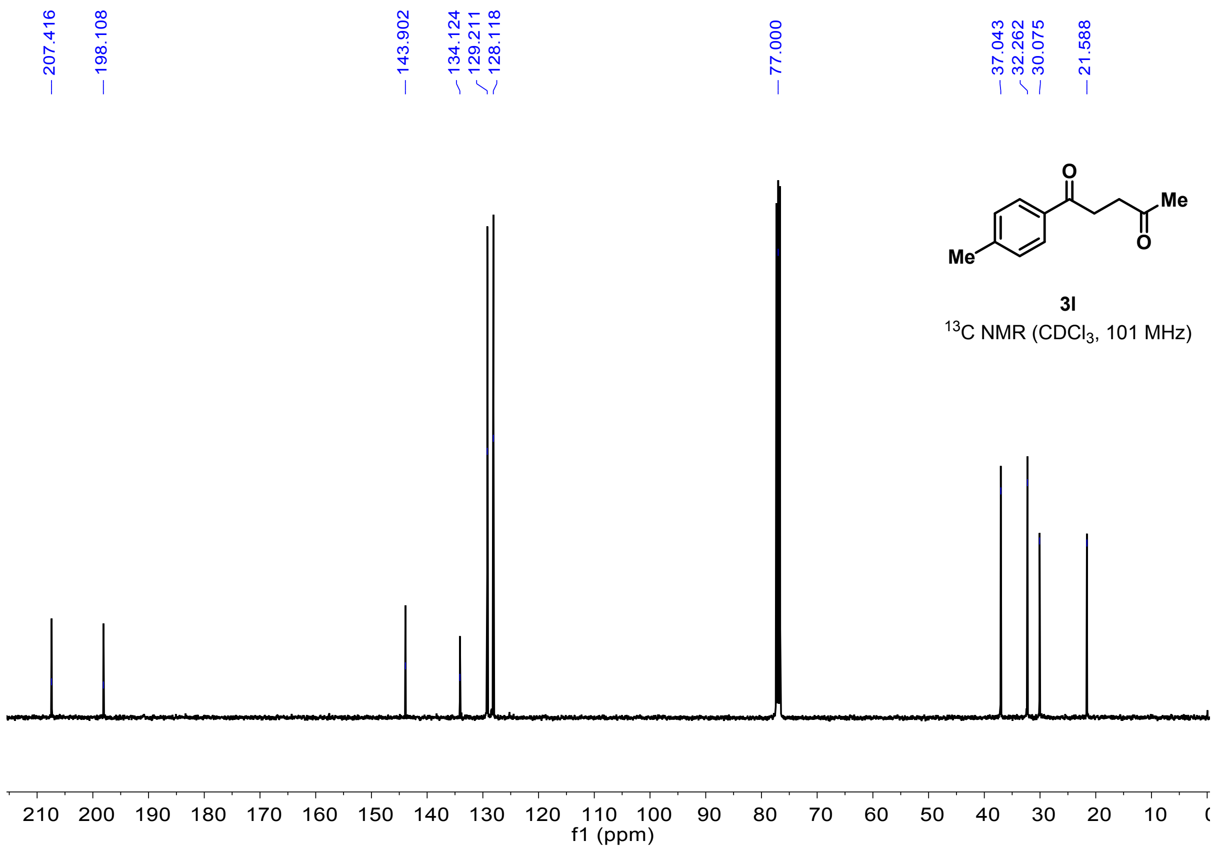
207.416
198.108
143.902
134.124
129.211
128.118
77.000
37.043
32.262
30.075
21.588
O
Me
Me
O
3l
13C NMR (CDCl3, 101 MHz)
210 200 190 180 170 160 150 140 130 120 110 100 90 80 70 60 50 40 30 20 10
f1 (ppm)

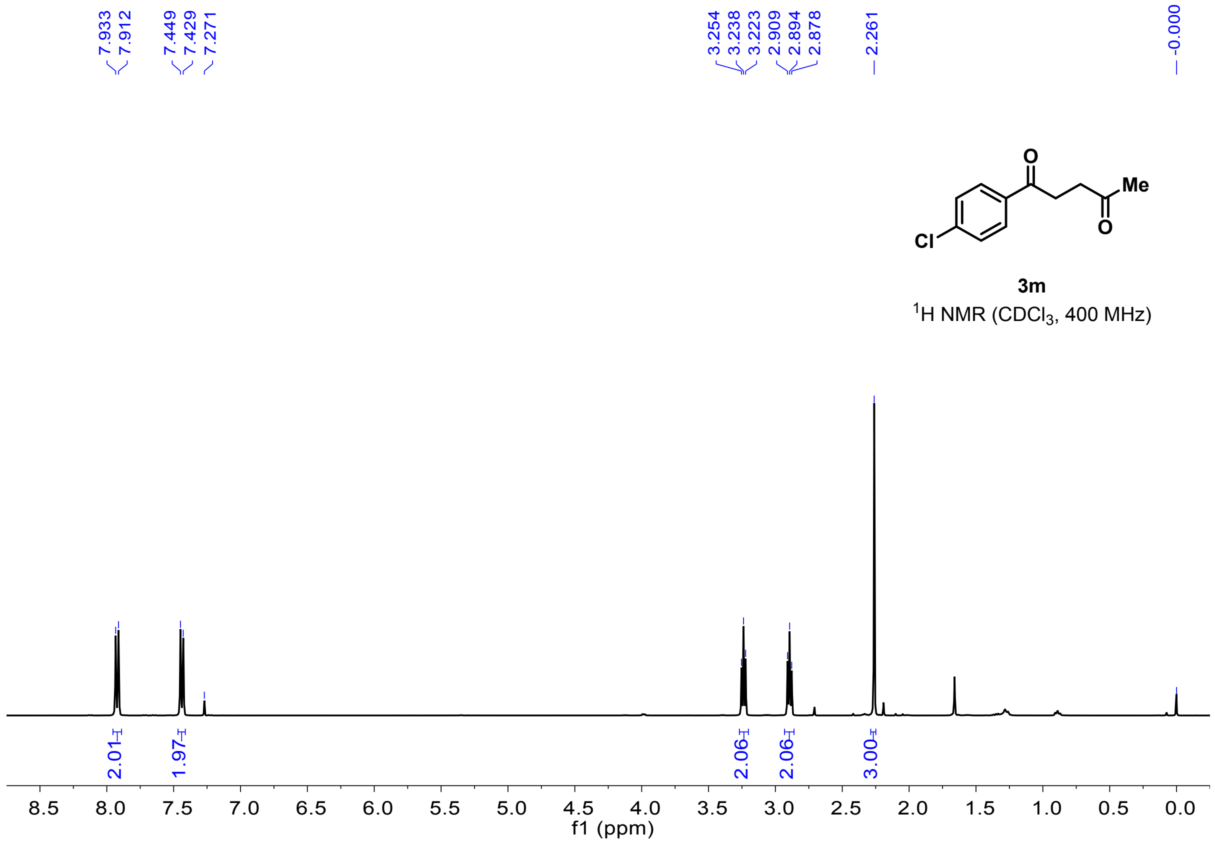
7.933
7.912
7.449
7.429
7.271
3.254
3.238
3.223
2.909
2.894
2.878
2.261
-0.000
O
Me
O
Cl
3m
1H NMR (CDCl3, 400 MHz)
2.01
1.97
2.06
2.06
3.00
8.5 8.0 7.5 7.0 6.5 6.0 5.5 5.0 4.5 4.0 3.5 3.0 2.5 2.0 1.5 1.0 0.5 0.0
f1 (ppm)

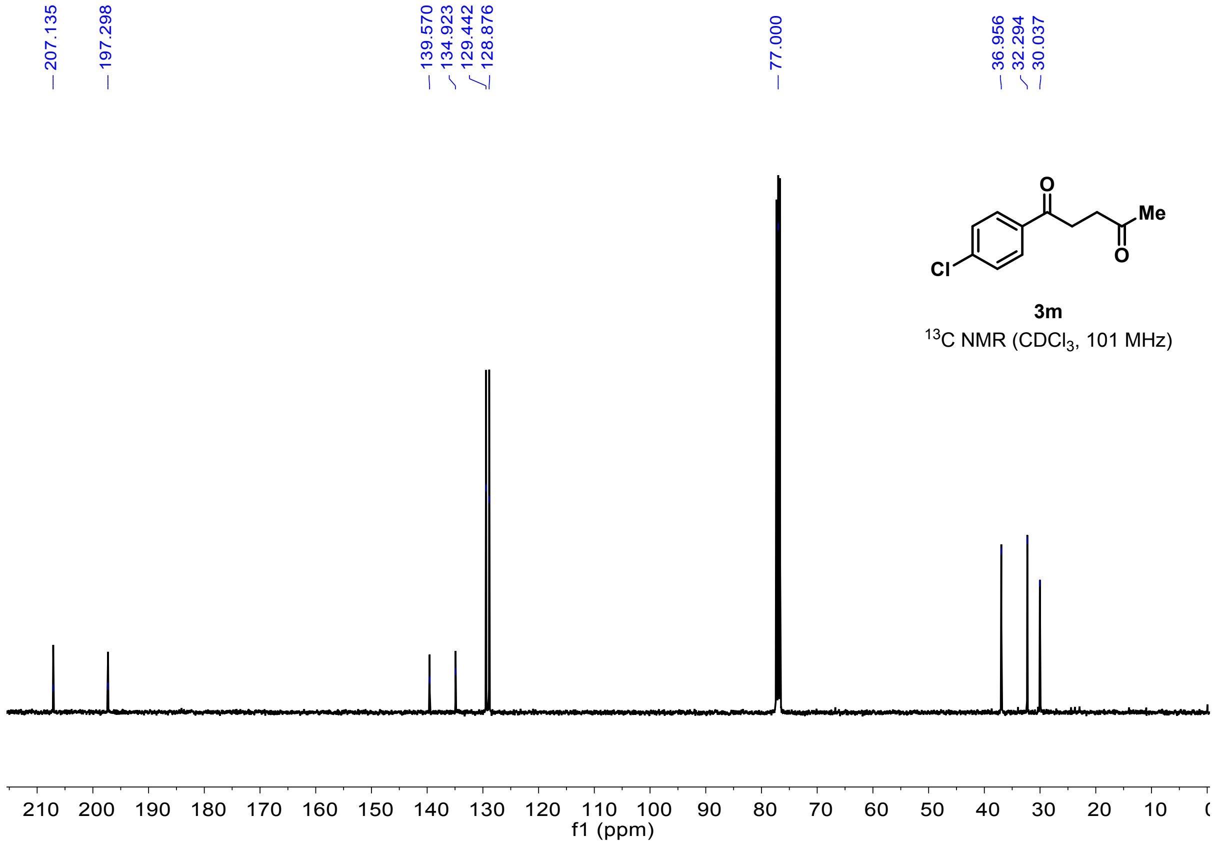
207.135
197.298
139.570
134.923
129.442
128.876
77.000
36.956
32.294
30.037
O
Me
O
Cl
3m
13C NMR (CDCl3, 101 MHz)
210 200 190 180 170 160 150 140 130 120 110 100 90 80 70 60 50 40 30 20 10
f1 (ppm)

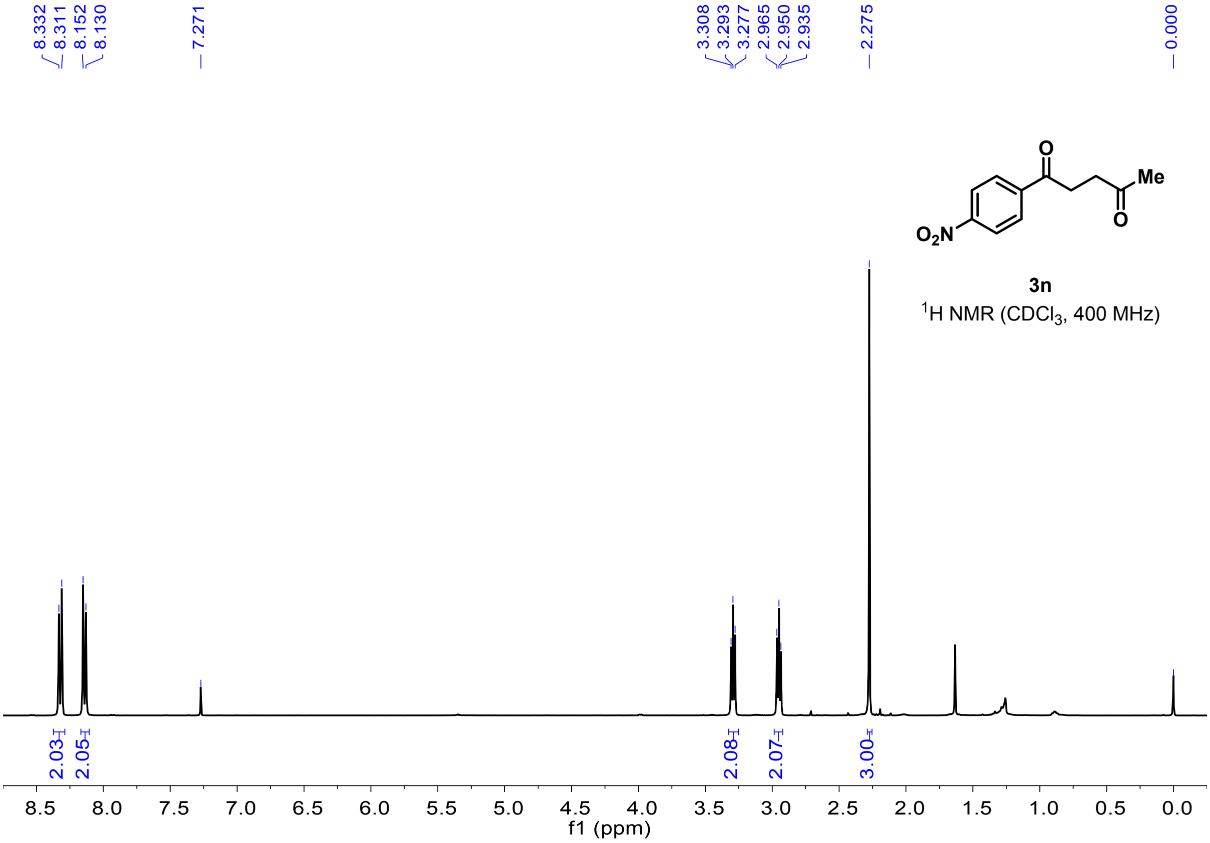
8.332
8.311
8.152
8.130
7.271
3.308
3.293
3.277
2.965
2.950
2.935
2.275
0.000
O
Me
O
O2N
3n
1H NMR (CDCl3, 400 MHz)
2.03
2.05
2.08
2.07
3.00
8.5 8.0 7.5 7.0 6.5 6.0 5.5 5.0 4.5 4.0 3.5 3.0 2.5 2.0 1.5 1.0 0.5 0.0
f1 (ppm)

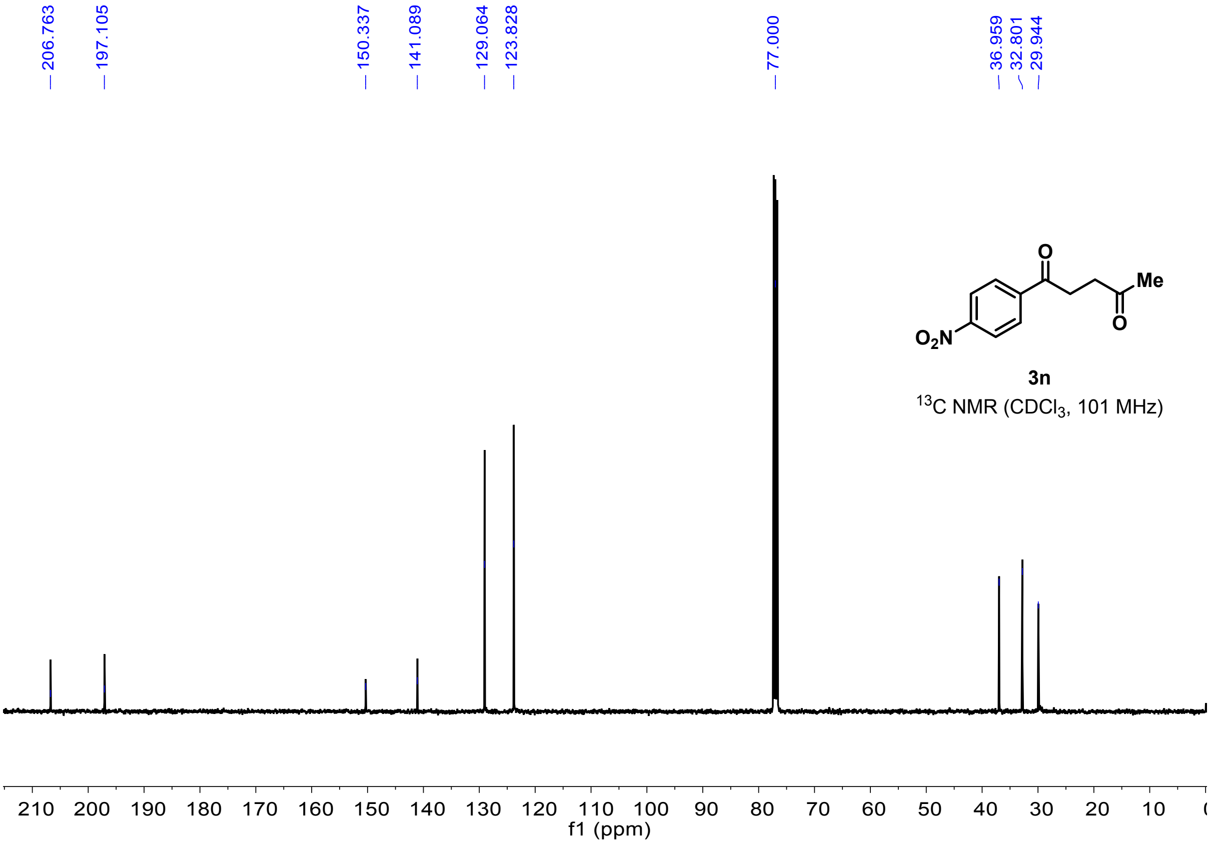
206.763
197.105
150.337
141.089
129.064
123.828
77.000
36.959
32.801
29.944
O
Me
O
O2N
3n
13C NMR (CDCl3, 101 MHz)
210 200 190 180 170 160 150 140 130 120 110 100 90 80 70 60 50 40 30 20 10
f1 (ppm)

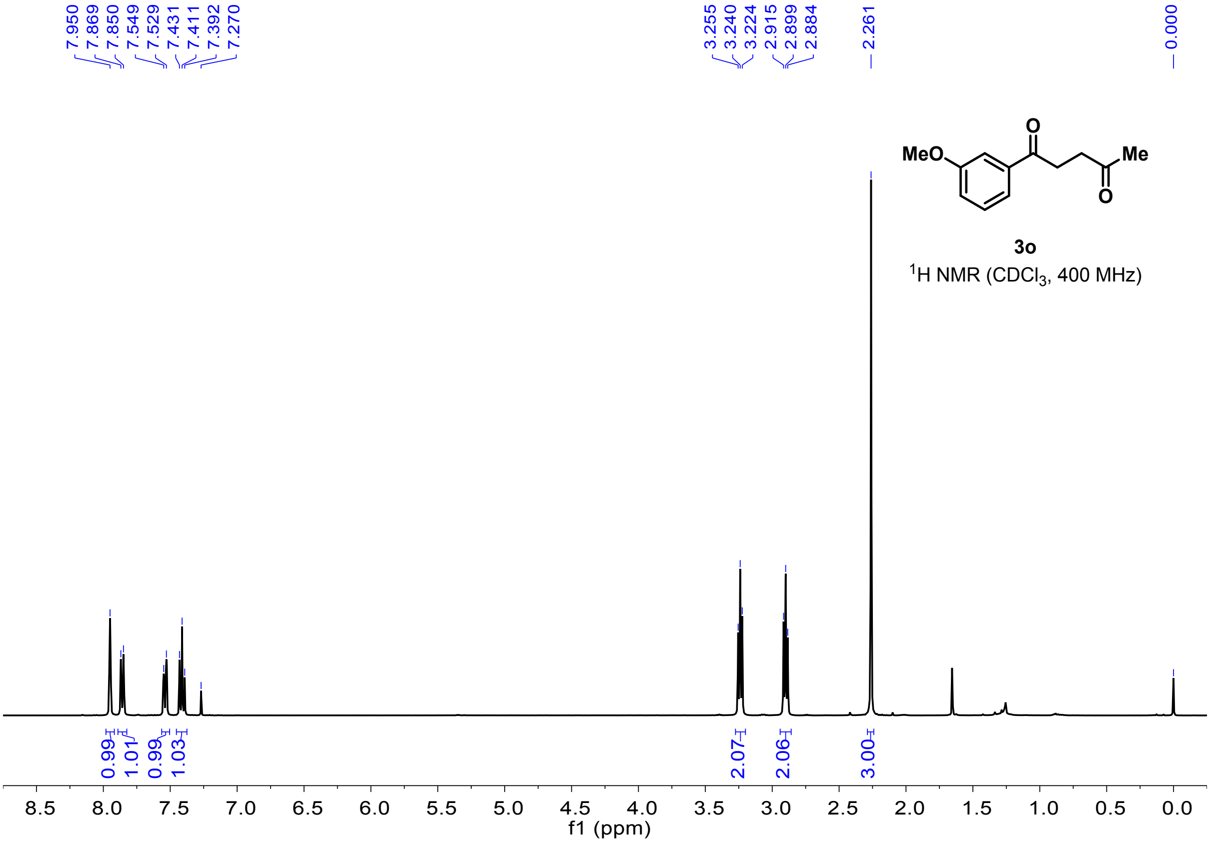
7.950
7.869
7.850
7.549
7.529
7.431
7.411
7.392
7.270
3.255
3.240
3.224
2.915
2.899
2.884
2.261
0.000
MeO
O
Me
O
3o
$^{1}$H NMR ($CDCl_3$, 400 MHz)
0.99
1.01
0.99
1.03
2.07
2.06
3.00
8.5 8.0 7.5 7.0 6.5 6.0 5.5 5.0 4.5 4.0 3.5 3.0 2.5 2.0 1.5 1.0 0.5 0.0
f1 (ppm)

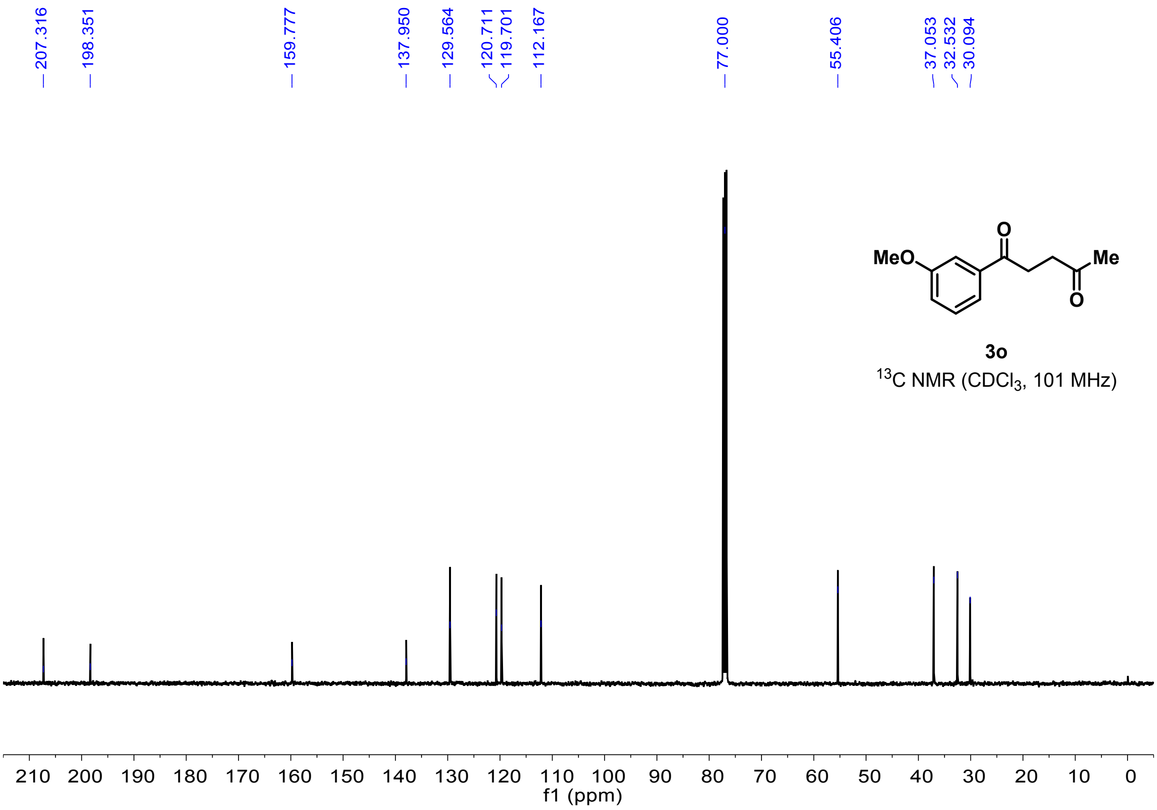
207.316
198.351
159.777
137.950
129.564
120.711
119.701
112.167
77.000
55.406
37.053
32.532
30.094
MeO
O
Me
O
3o
$^{13}$C NMR ($CDCl_3$, 101 MHz)
210 200 190 180 170 160 150 140 130 120 110 100 90 80 70 60 50 40 30 20 10 0
f1 (ppm)

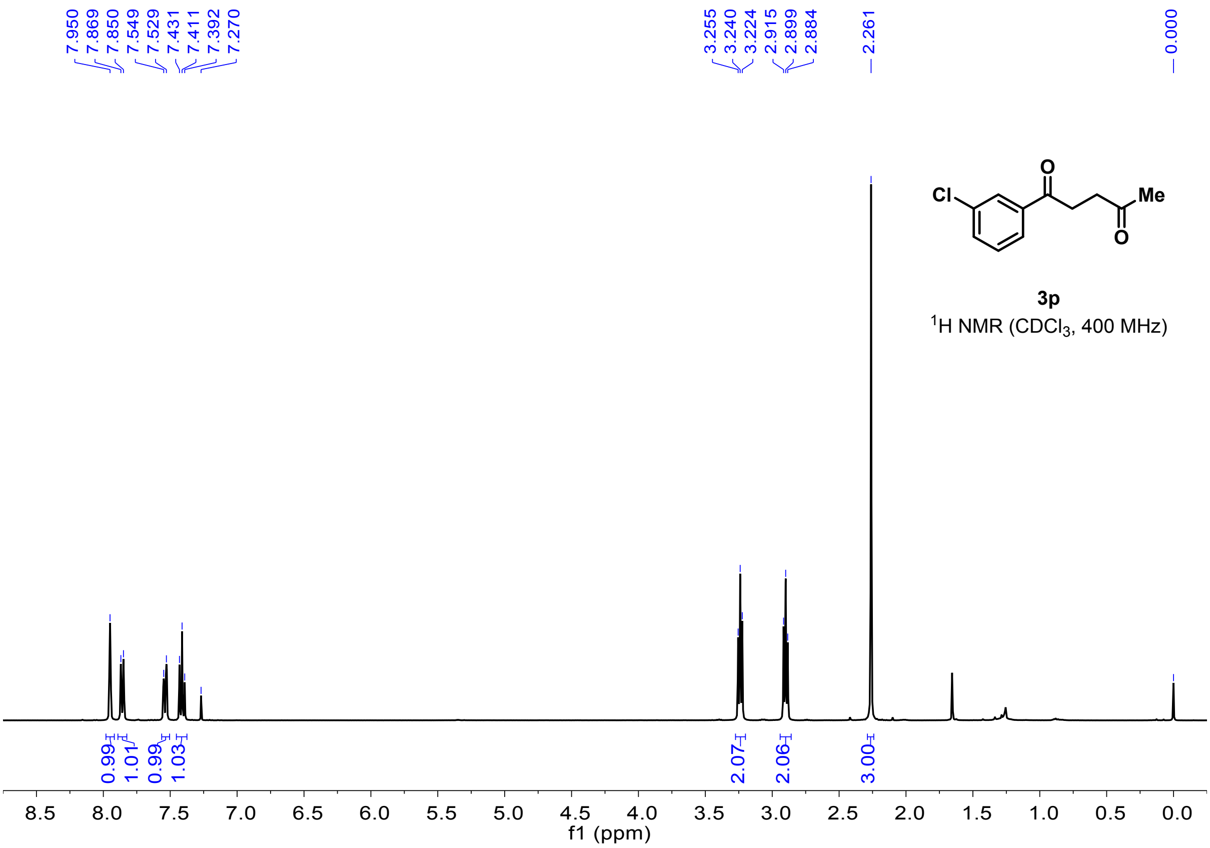

7.950
7.869
7.850
7.549
7.529
7.431
7.411
7.392
7.270
3.255
3.240
3.224
2.915
2.899
2.884
2.261
0.000
Cl
O
Me
O
3p
¹H NMR (CDCl₃, 400 MHz)
0.99
1.01
0.99
1.03
2.07
2.06
3.00
8.5 8.0 7.5 7.0 6.5 6.0 5.5 5.0 4.5 4.0 3.5 3.0 2.5 2.0 1.5 1.0 0.5 0.0
f1 (ppm)


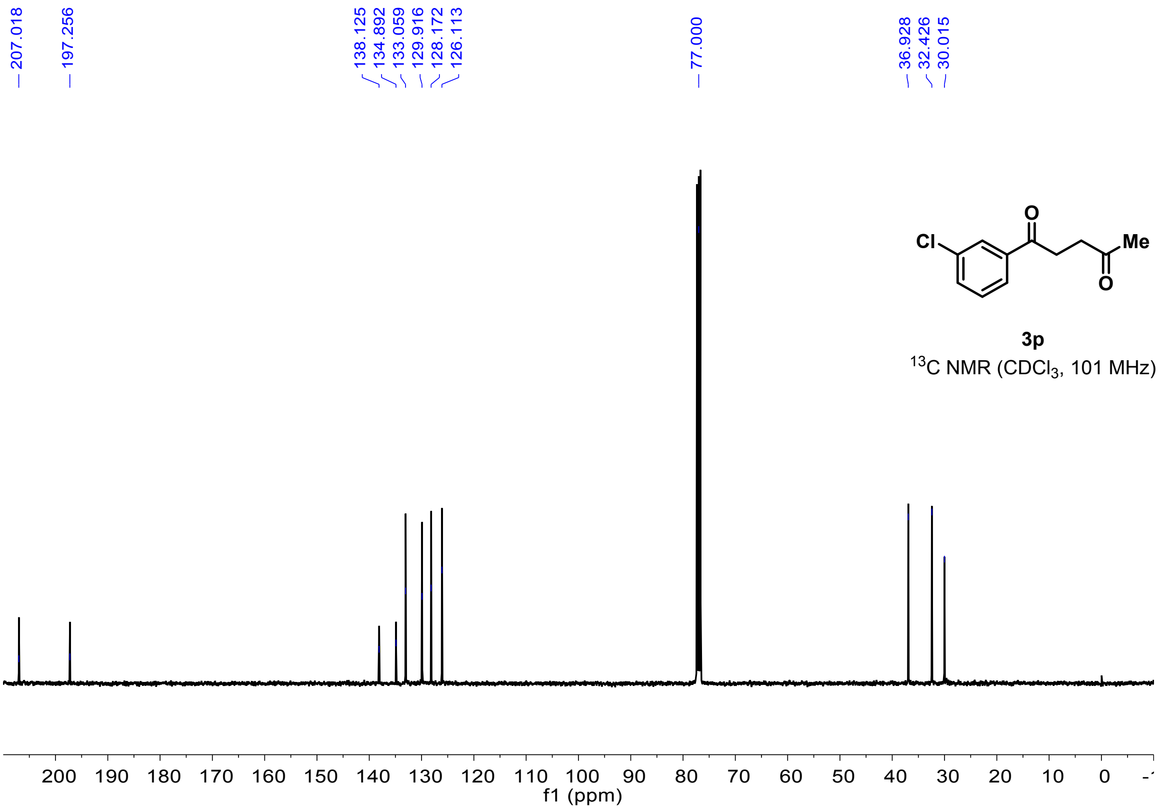

207.018
197.256
138.125
134.892
133.059
129.916
128.172
126.113
77.000
36.928
32.426
30.015
Cl
O
Me
O
3p
¹³C NMR (CDCl₃, 101 MHz)
200 190 180 170 160 150 140 130 120 110 100 90 80 70 60 50 40 30 20 10 0
f1 (ppm)

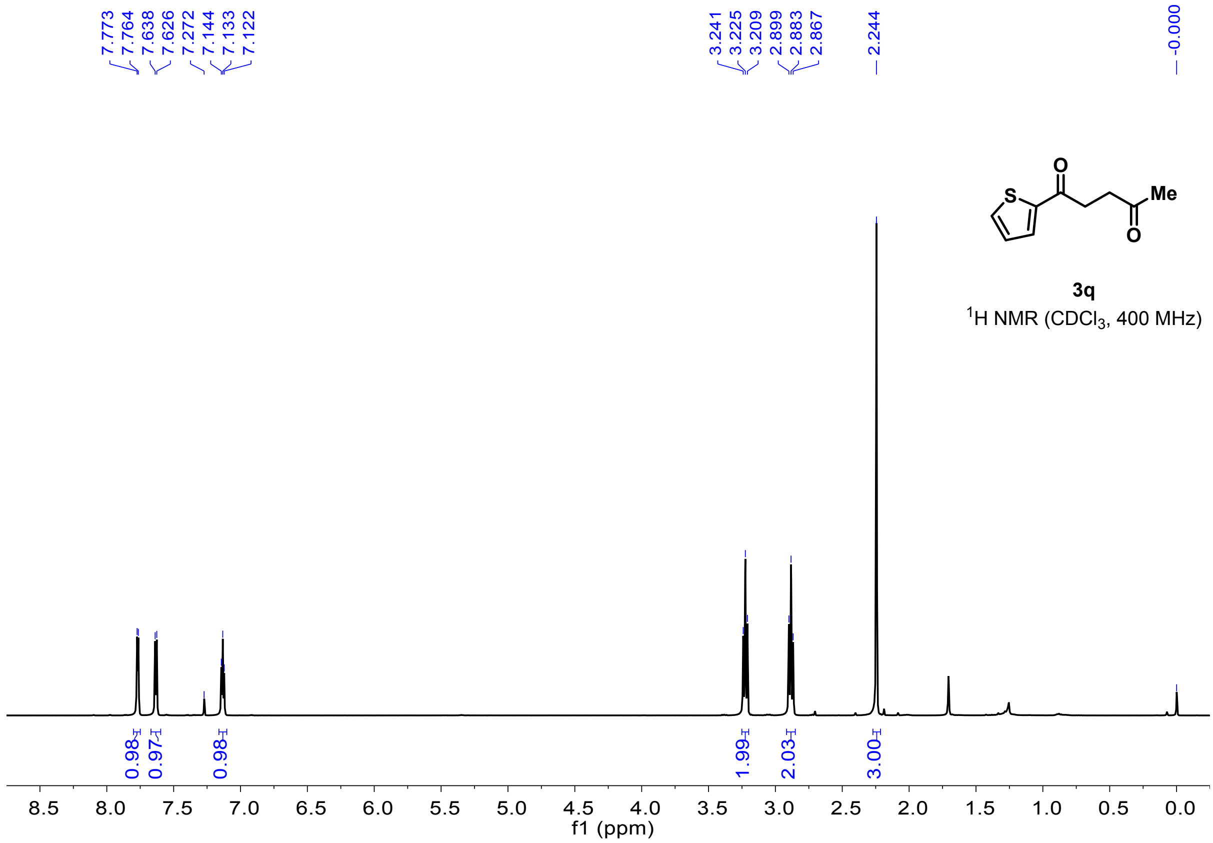
7.773
7.764
7.638
7.626
7.272
7.144
7.133
7.122
3.241
3.225
3.209
2.899
2.883
2.867
2.244
-0.000
S
O
Me
O
3q
1H NMR (CDCl3, 400 MHz)
0.98
0.97
0.98
1.99
2.03
3.00
8.5 8.0 7.5 7.0 6.5 6.0 5.5 5.0 4.5 4.0 3.5 3.0 2.5 2.0 1.5 1.0 0.5 0.0
f1 (ppm)

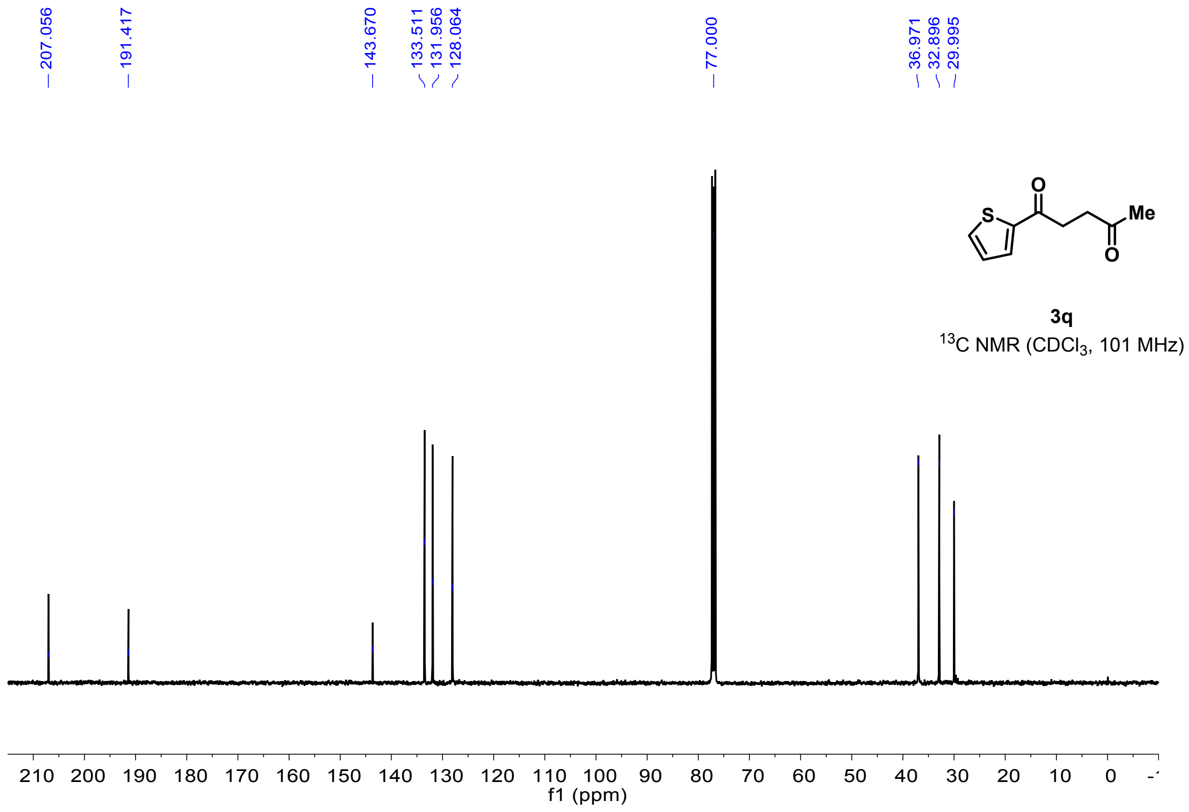
207.056
191.417
143.670
133.511
131.956
128.064
77.000
36.971
32.896
29.995
S
O
Me
O
3q
13C NMR (CDCl3, 101 MHz)
210 200 190 180 170 160 150 140 130 120 110 100 90 80 70 60 50 40 30 20 10 0
f1 (ppm)

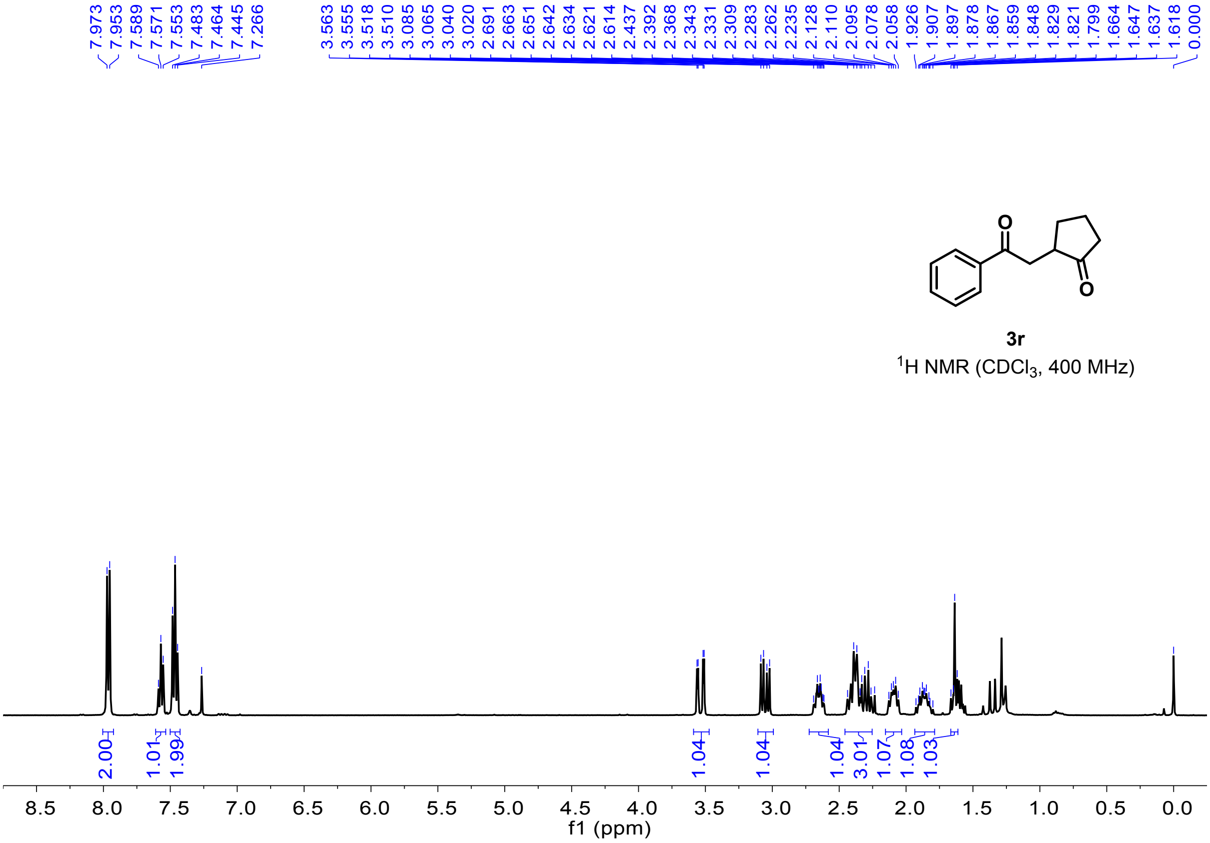


**3r**

$^{1}H$ NMR ($CDCl_3$, 400 MHz)

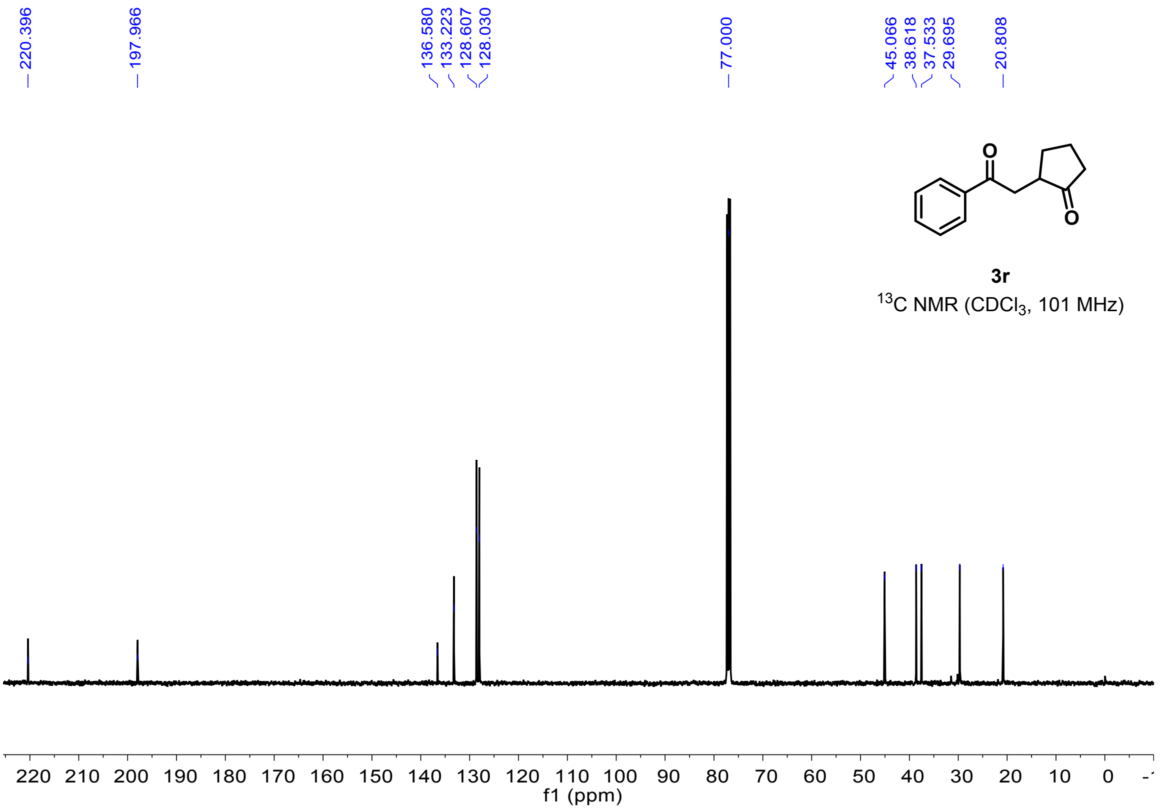


**3r**

$^{13}C$ NMR ($CDCl_3$, 101 MHz)

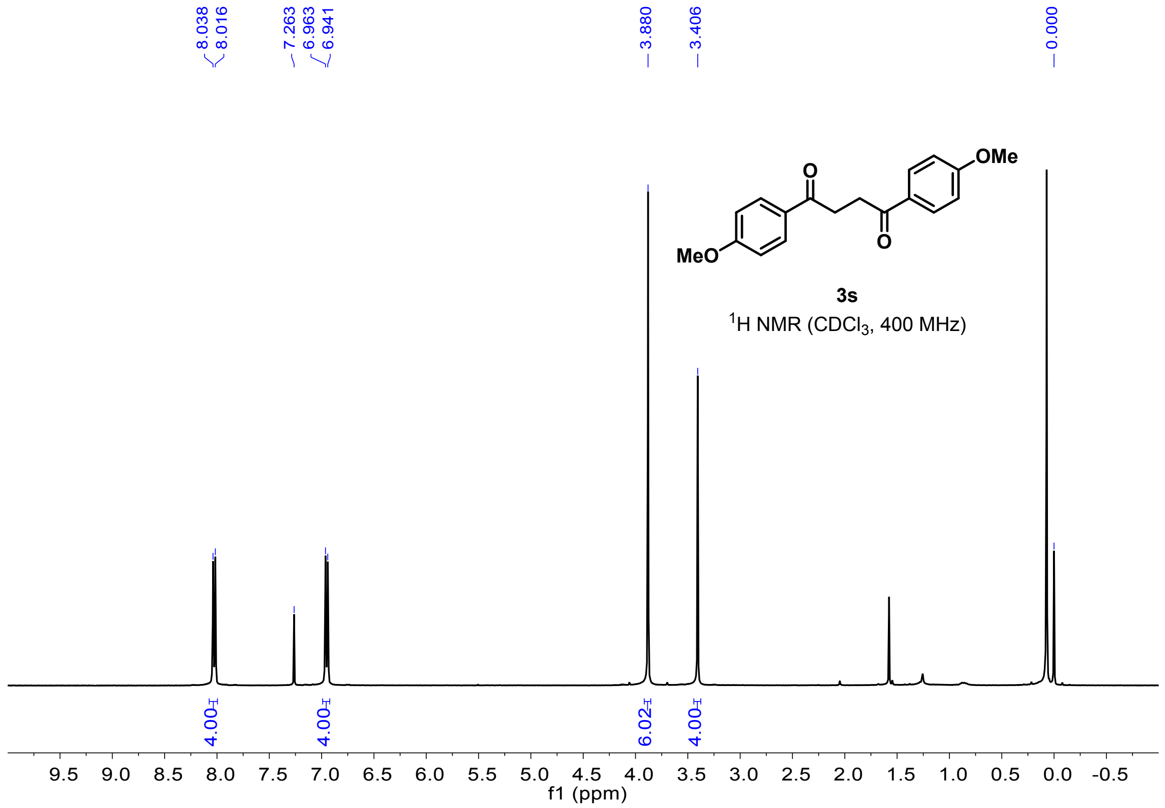
8.038
8.016
7.263
6.963
6.941
3.880
3.406
0.000
OMe
MeO
O
O
3s
¹H NMR (CDCl₃, 400 MHz)
4.00
4.00
6.02
4.00
9.5 9.0 8.5 8.0 7.5 7.0 6.5 6.0 5.5 5.0 4.5 4.0 3.5 3.0 2.5 2.0 1.5 1.0 0.5 0.0 -0.5
f1 (ppm)

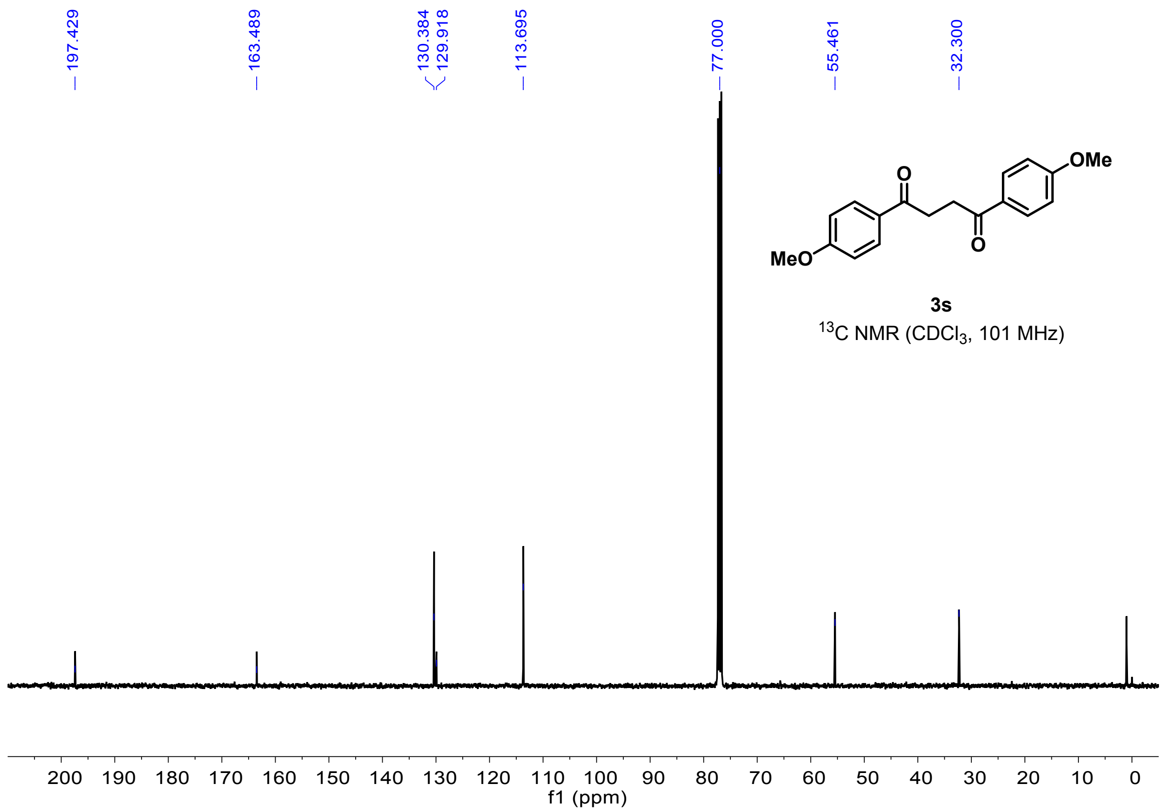
197.429
163.489
130.384
129.918
113.695
77.000
55.461
32.300
OMe
MeO
O
O
3s
¹³C NMR (CDCl₃, 101 MHz)
200 190 180 170 160 150 140 130 120 110 100 90 80 70 60 50 40 30 20 10 0
f1 (ppm)

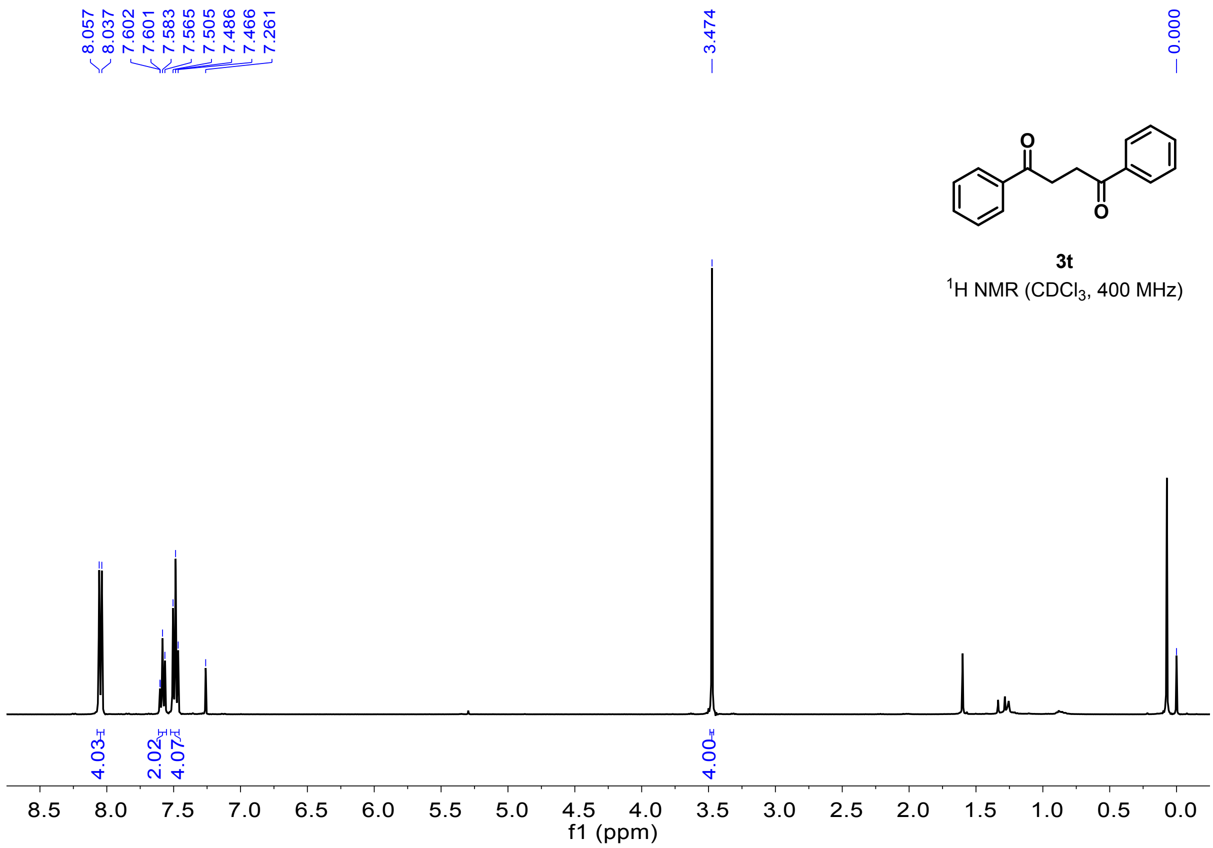
8.057
8.037
7.602
7.601
7.583
7.565
7.505
7.486
7.466
7.261
3.474
0.000
O
O
3t
$^1$H NMR (CDCl$_3$, 400 MHz)
4.03
2.02
4.07
4.00
8.5 8.0 7.5 7.0 6.5 6.0 5.5 5.0 4.5 4.0 3.5 3.0 2.5 2.0 1.5 1.0 0.5 0.0
f1 (ppm)

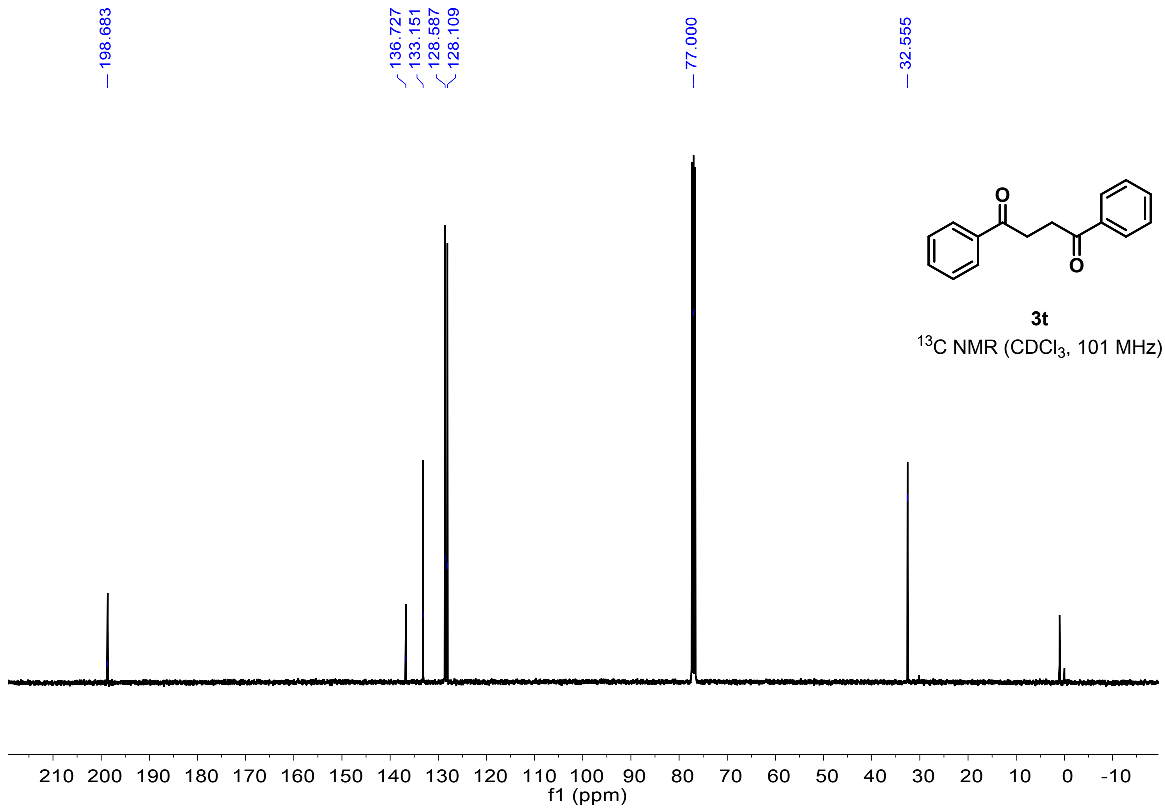
198.683
136.727
133.151
128.587
128.109
77.000
32.555
O
O
3t
$^{13}$C NMR (CDCl$_3$, 101 MHz)
210 200 190 180 170 160 150 140 130 120 110 100 90 80 70 60 50 40 30 20 10 0 -10
f1 (ppm)

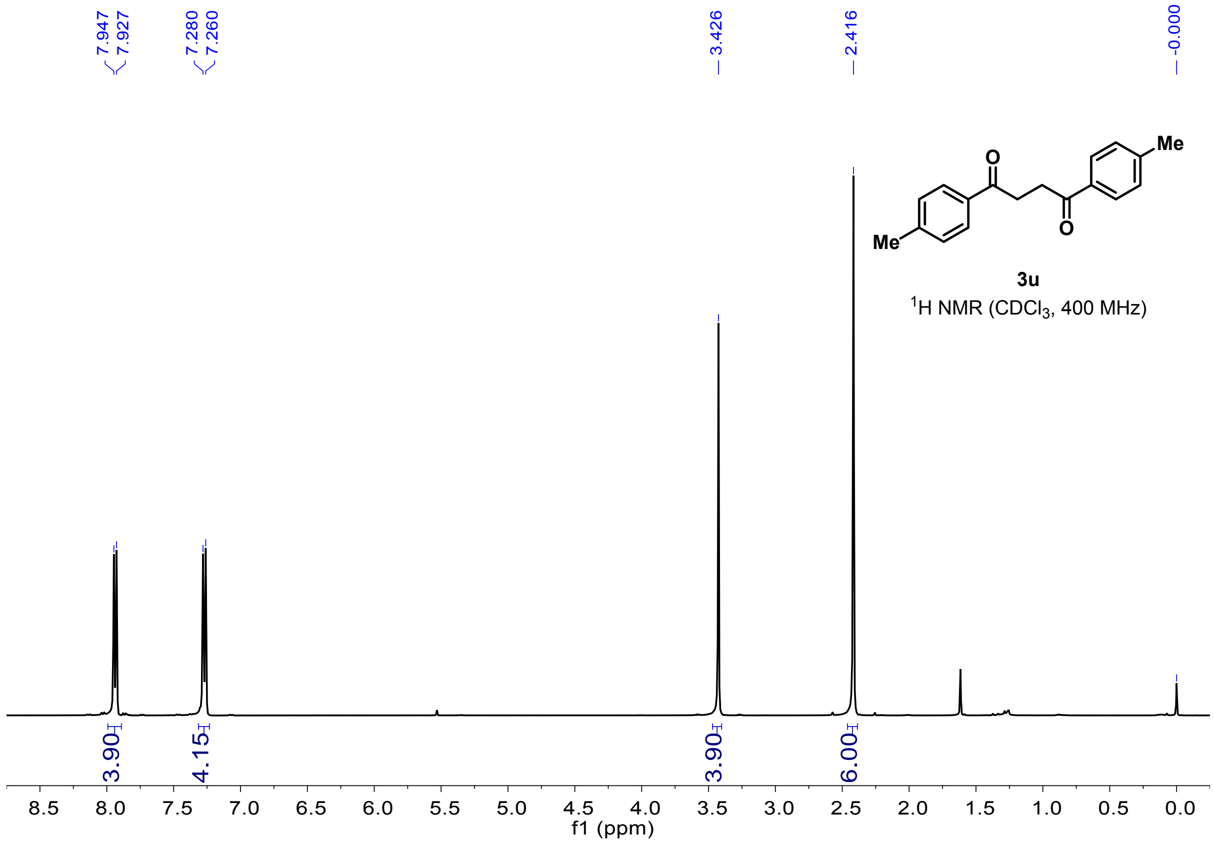
7.947
7.927
7.280
7.260
3.426
2.416
-0.000
Me
O
O
Me
3u
¹H NMR (CDCl3, 400 MHz)
3.90
4.15
3.90
6.00
8.5 8.0 7.5 7.0 6.5 6.0 5.5 5.0 4.5 4.0 3.5 3.0 2.5 2.0 1.5 1.0 0.5 0.0
f1 (ppm)

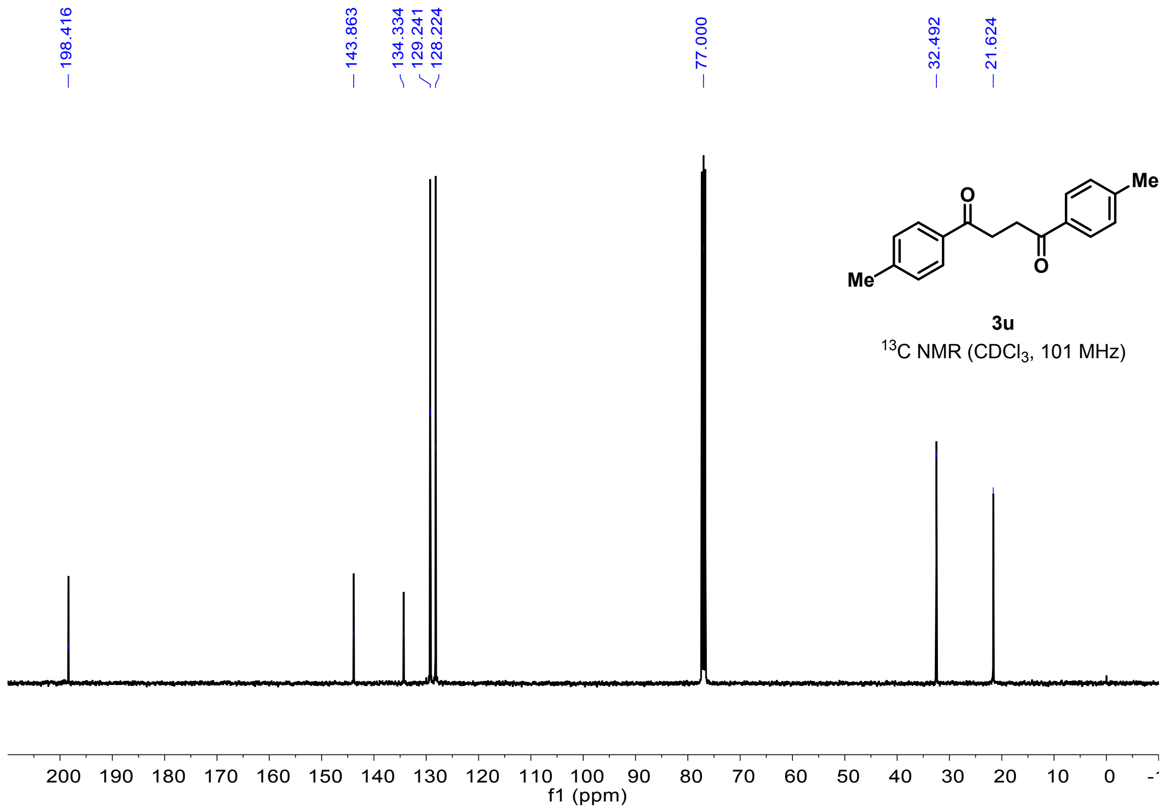
198.416
143.863
134.334
129.241
128.224
77.000
32.492
21.624
Me
O
O
Me
3u
¹³C NMR (CDCl3, 101 MHz)
200 190 180 170 160 150 140 130 120 110 100 90 80 70 60 50 40 30 20 10 0
f1 (ppm)

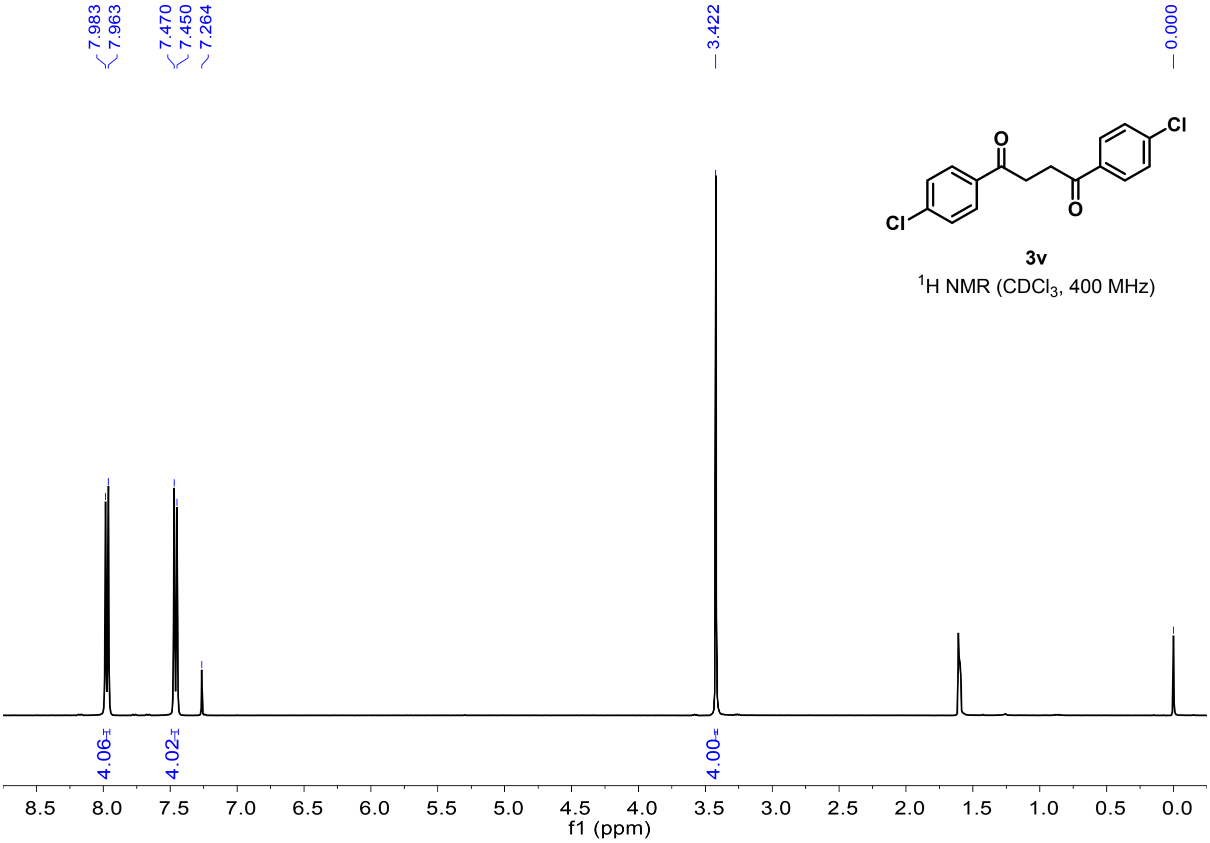
7.983
7.963
7.470
7.450
7.264
3.422
0.000
Cl
O
O
Cl
3v
$^{1}$H NMR ($CDCl_3$, 400 MHz)
4.06
4.02
4.00
8.5 8.0 7.5 7.0 6.5 6.0 5.5 5.0 4.5 4.0 3.5 3.0 2.5 2.0 1.5 1.0 0.5 0.0
f1 (ppm)

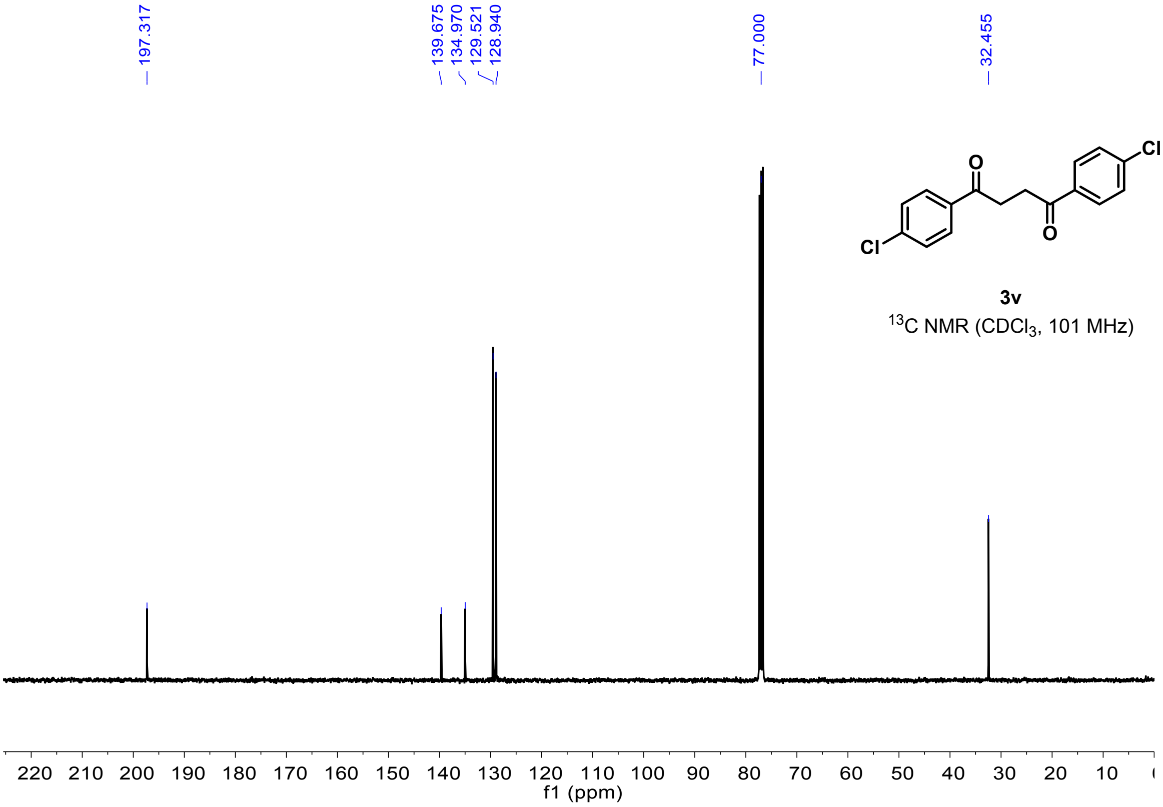
197.317
139.675
134.970
129.521
128.940
77.000
32.455
Cl
O
O
Cl
3v
$^{13}$C NMR ($CDCl_3$, 101 MHz)
220 210 200 190 180 170 160 150 140 130 120 110 100 90 80 70 60 50 40 30 20 10
f1 (ppm)

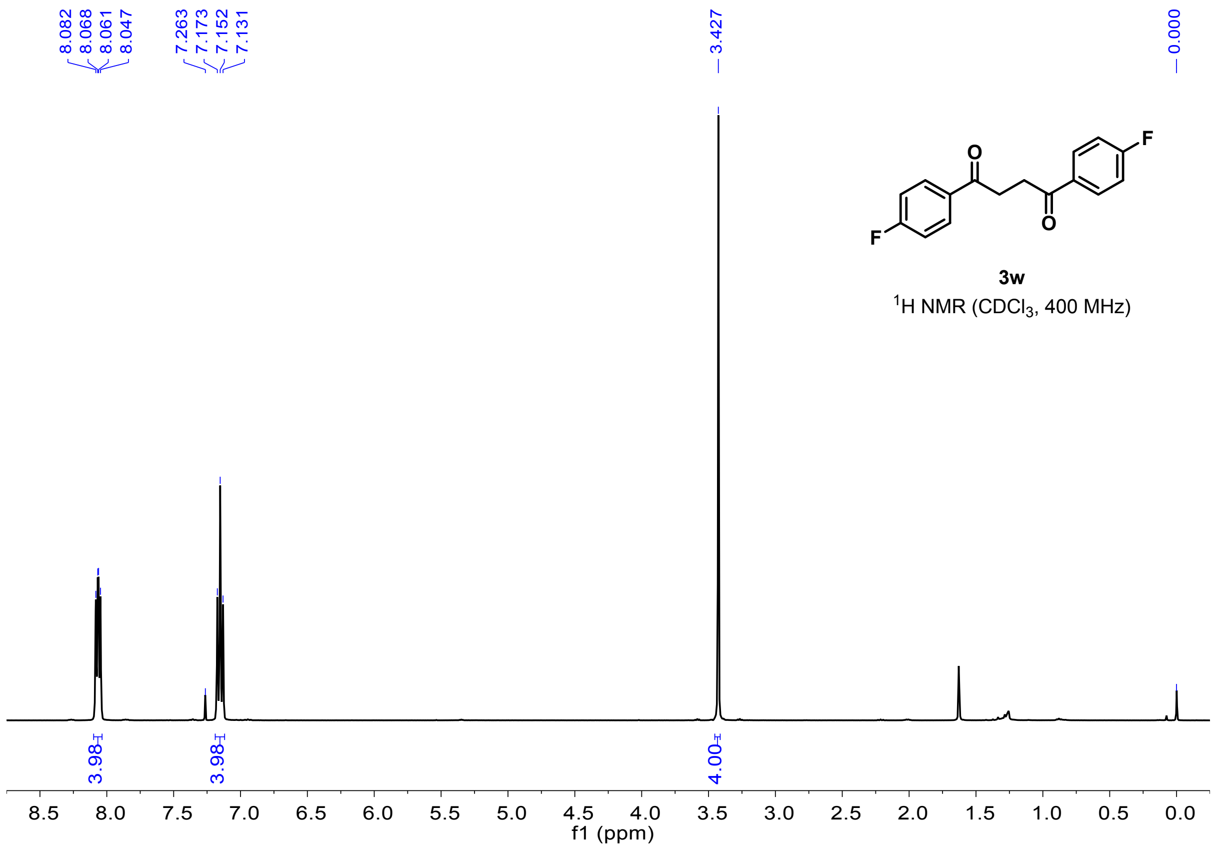
8.082
8.068
8.061
8.047
7.263
7.173
7.152
7.131
3.427
0.000
3.98
3.98
4.00
3w
$^{1}H$ NMR ($CDCl_3$, 400 MHz)
f1 (ppm)

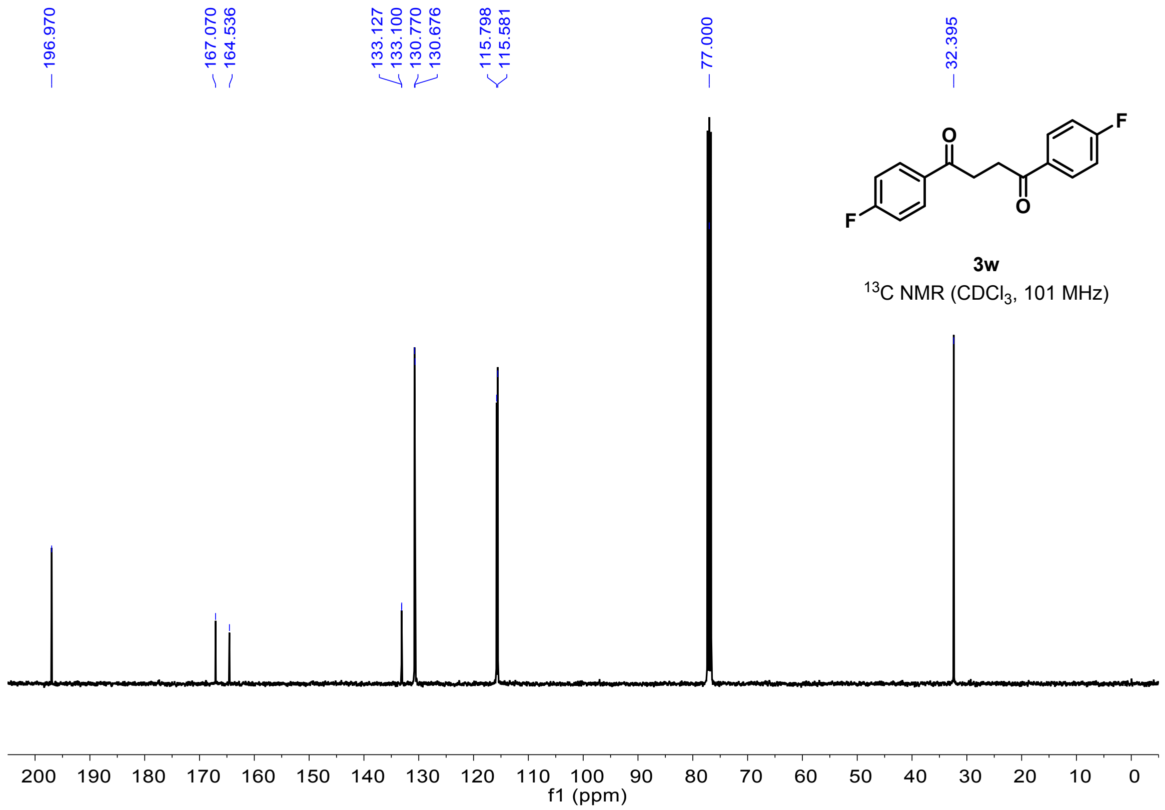
196.970
167.070
164.536
133.127
133.100
130.770
130.676
115.798
115.581
77.000
32.395
3w
$^{13}C$ NMR ($CDCl_3$, 101 MHz)
f1 (ppm)

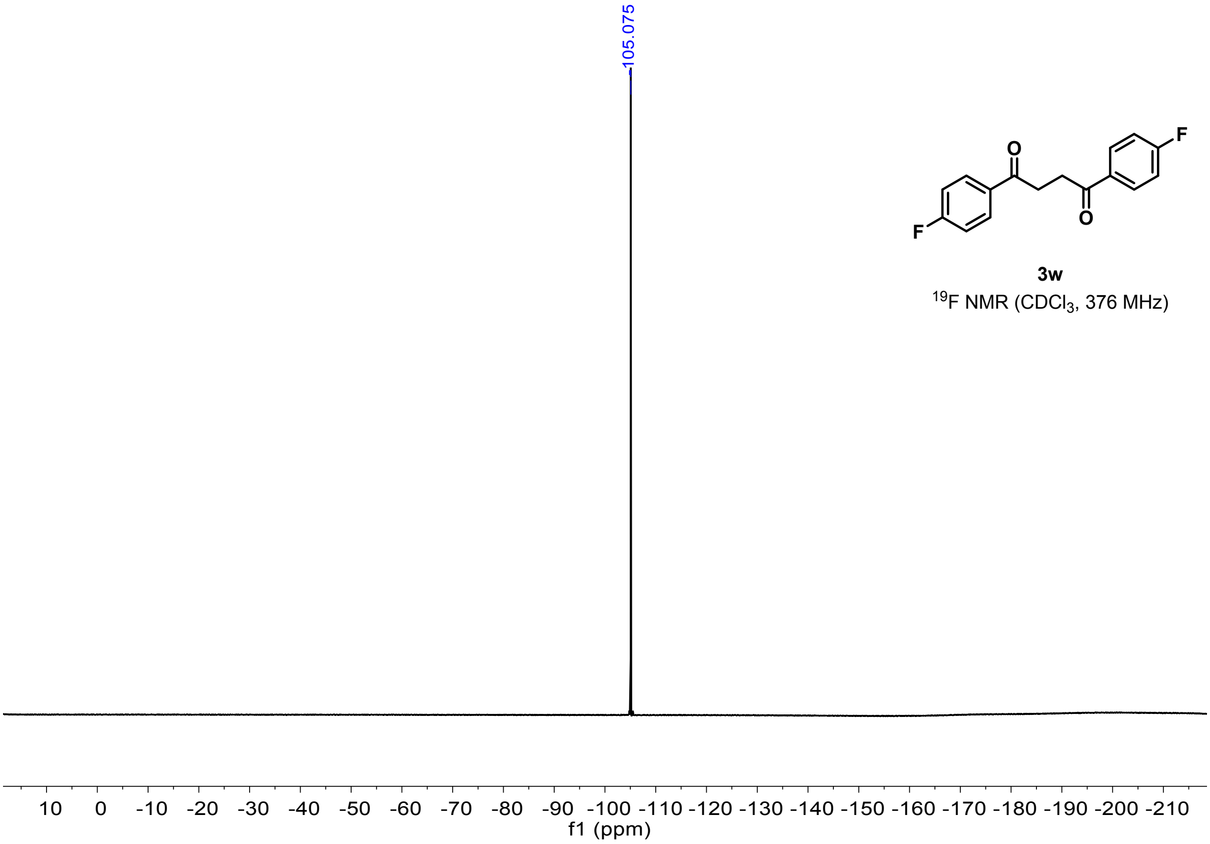


**3w**

$^{19}F$ NMR ($CDCl_3$, 376 MHz)

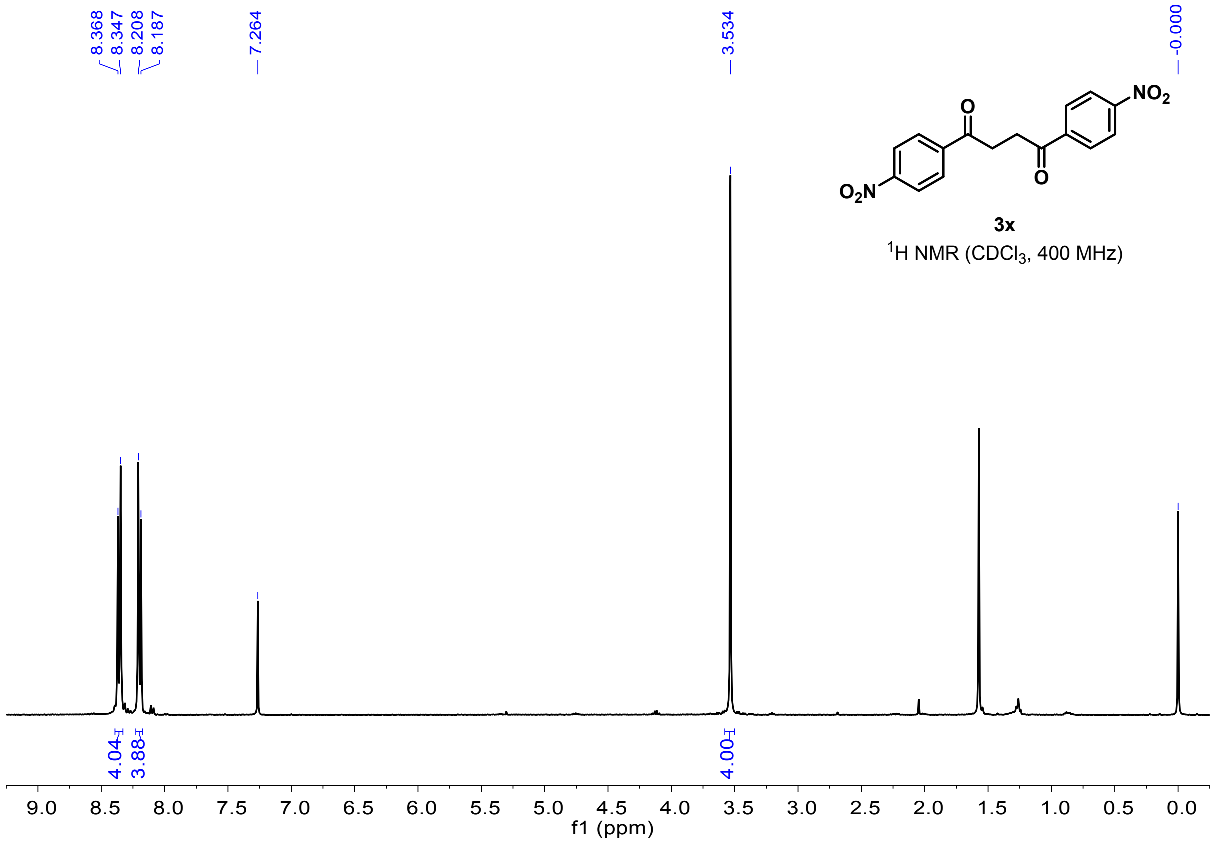
8.368
8.347
8.208
8.187
7.264
3.534
-0.000
NO2
O
O
O2N
3x
1H NMR (CDCl3, 400 MHz)
4.04
3.88
4.00
9.0 8.5 8.0 7.5 7.0 6.5 6.0 5.5 5.0 4.5 4.0 3.5 3.0 2.5 2.0 1.5 1.0 0.5 0.0
f1 (ppm)

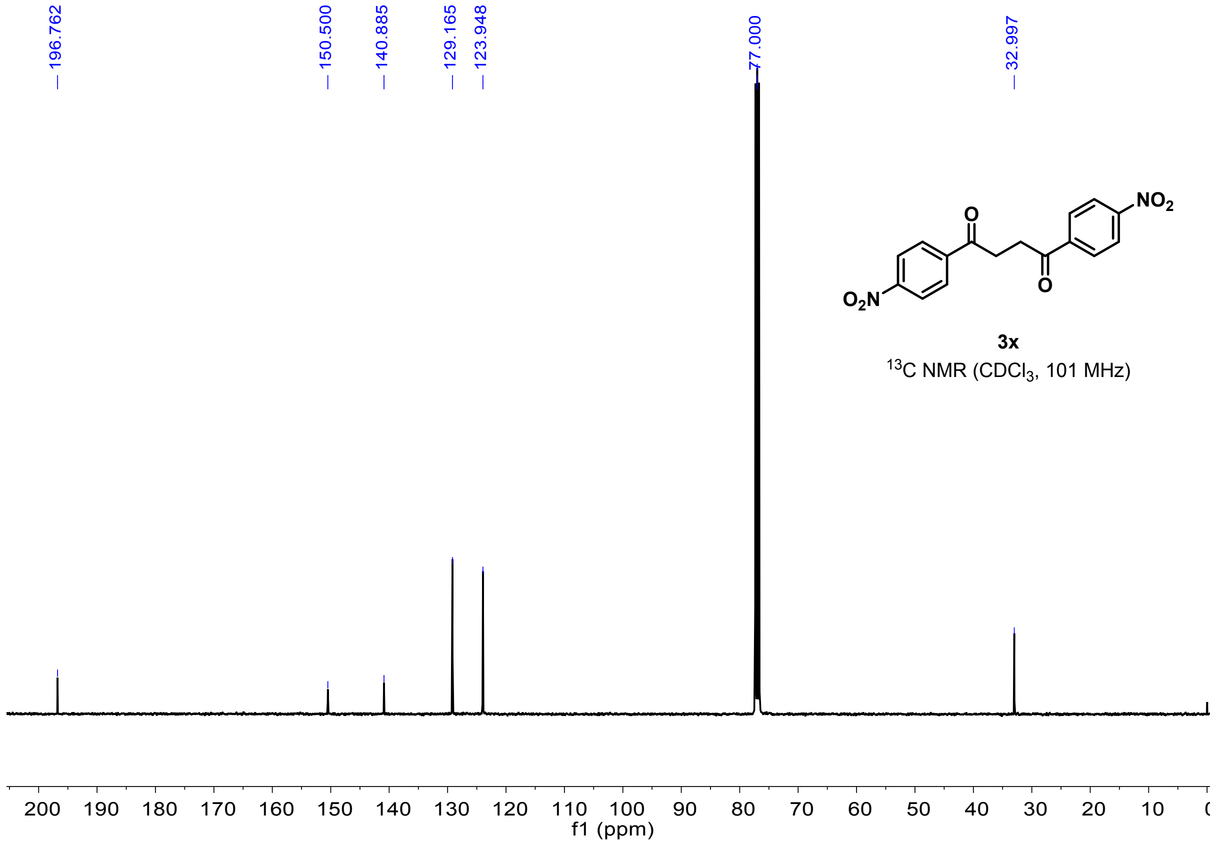
196.762
150.500
140.885
129.165
123.948
77.000
32.997
NO2
O
O
O2N
3x
13C NMR (CDCl3, 101 MHz)
200 190 180 170 160 150 140 130 120 110 100 90 80 70 60 50 40 30 20 10
f1 (ppm)

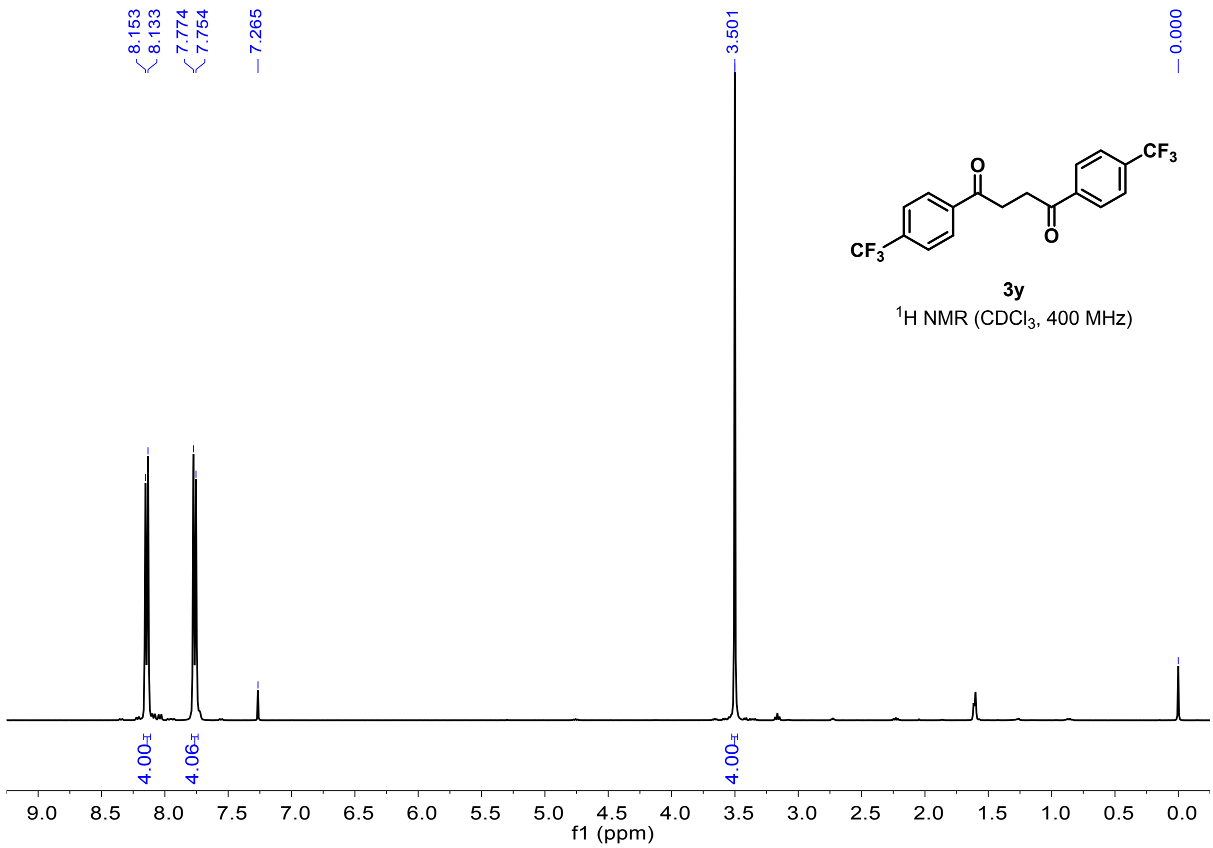
8.153
8.133
7.774
7.754
7.265
3.501
0.000
CF3
O
O
CF3
3y
1H NMR (CDCl3, 400 MHz)
4.00
4.06
4.00
9.0 8.5 8.0 7.5 7.0 6.5 6.0 5.5 5.0 4.5 4.0 3.5 3.0 2.5 2.0 1.5 1.0 0.5 0.0
f1 (ppm)

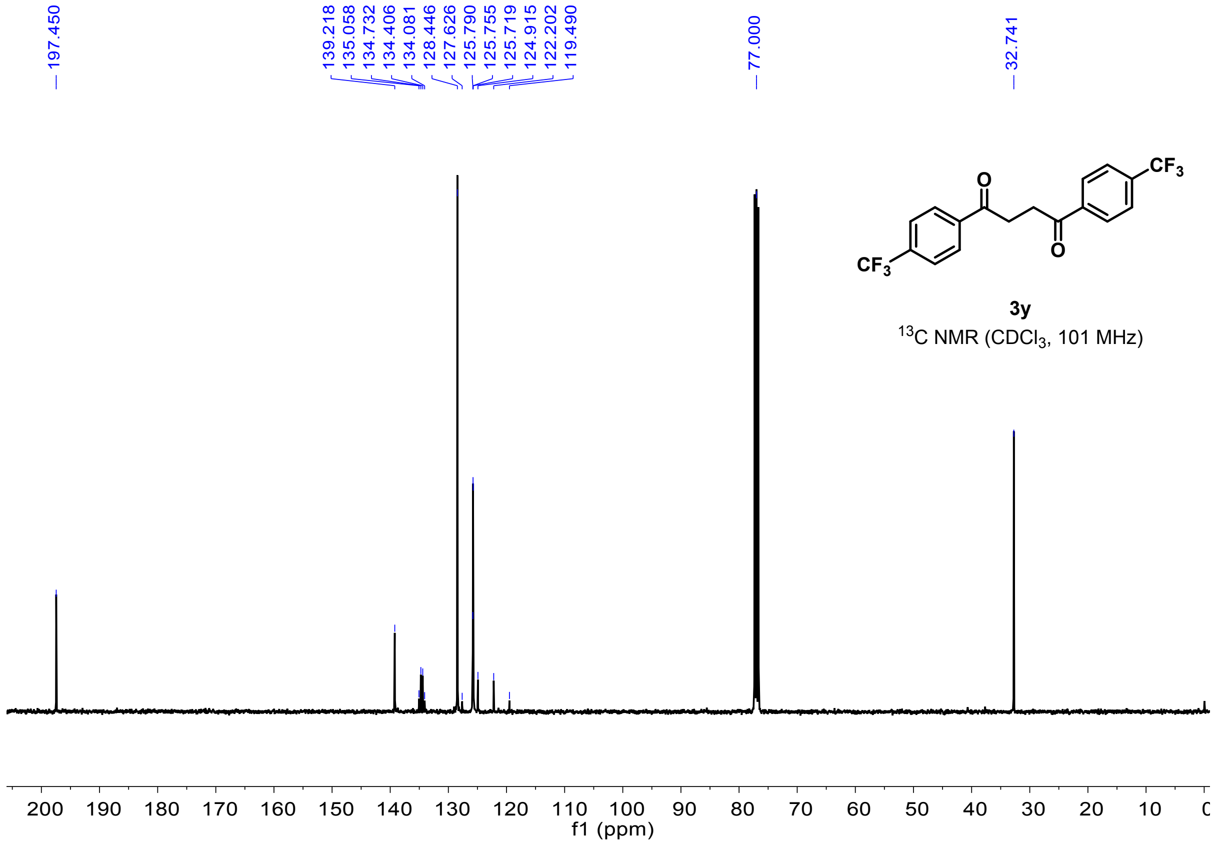
197.450
139.218
135.058
134.732
134.406
134.081
128.446
127.626
125.790
125.755
125.719
124.915
122.202
119.490
77.000
32.741
CF3
O
O
CF3
3y
13C NMR (CDCl3, 101 MHz)
200 190 180 170 160 150 140 130 120 110 100 90 80 70 60 50 40 30 20 10 0
f1 (ppm)

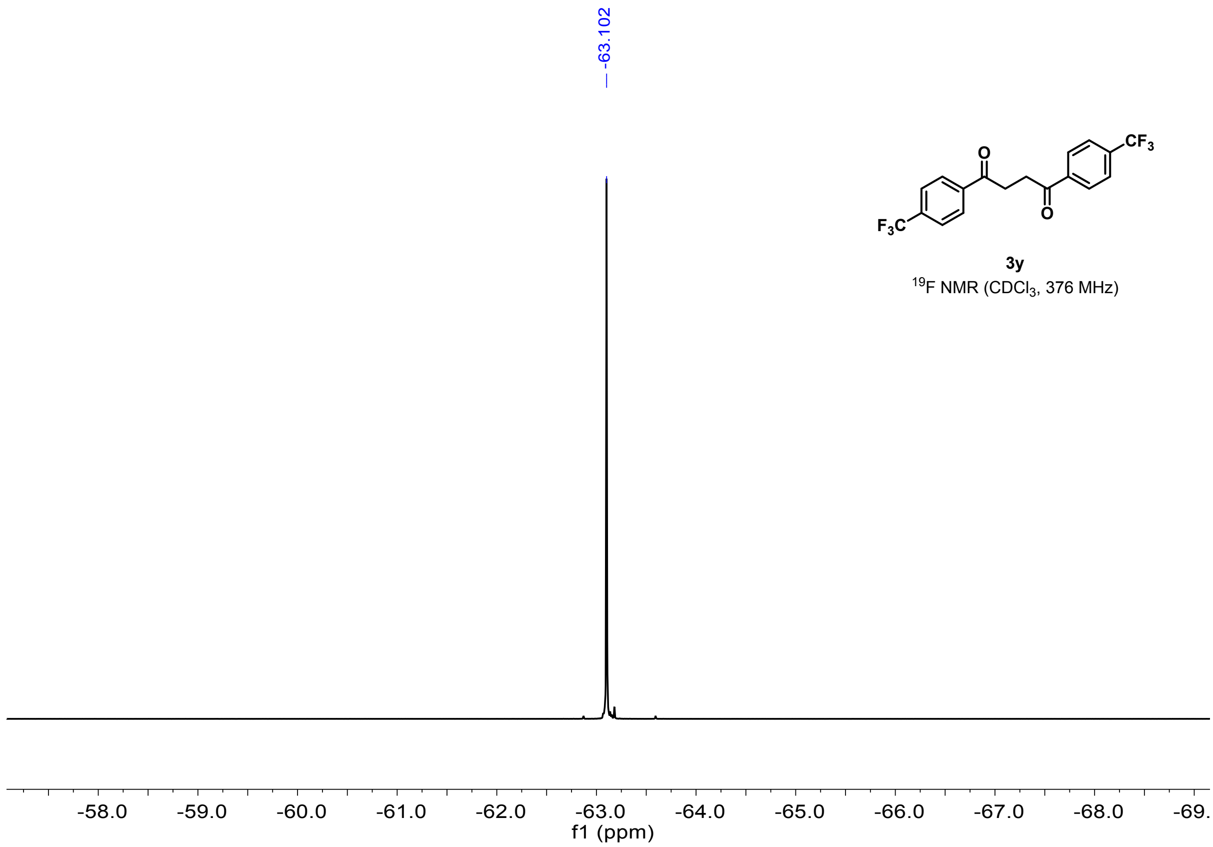

-63.102
CF3
O
O
F3C
3y
19F NMR (CDCl3, 376 MHz)
-58.0
-59.0
-60.0
-61.0
-62.0
-63.0
-64.0
-65.0
-66.0
-67.0
-68.0
-69.
f1 (ppm)

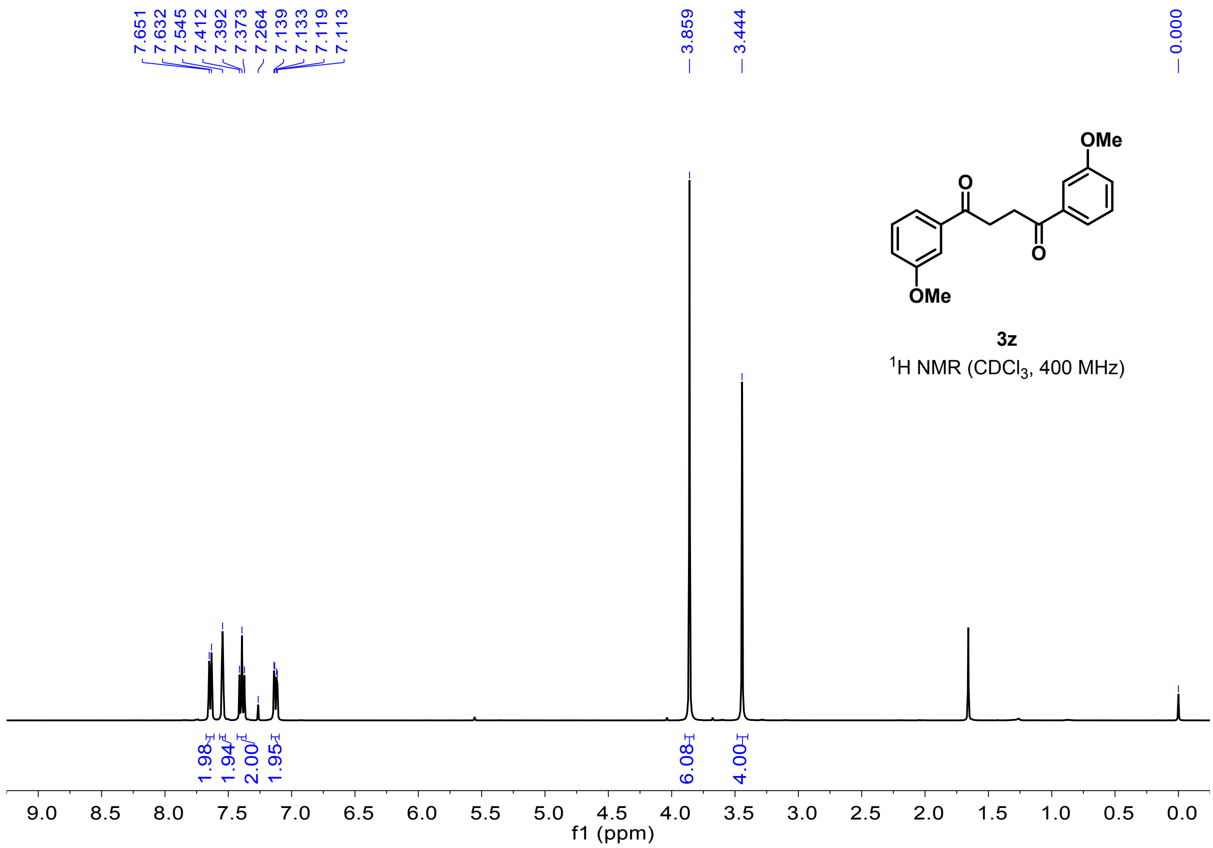
7.651
7.632
7.545
7.412
7.392
7.373
7.264
7.139
7.133
7.119
7.113
3.859
3.444
0.000
OMe
O
O
OMe
3z
1H NMR (CDCl3, 400 MHz)
1.98
1.94
2.00
1.95
6.08
4.00
9.0 8.5 8.0 7.5 7.0 6.5 6.0 5.5 5.0 4.5 4.0 3.5 3.0 2.5 2.0 1.5 1.0 0.5 0.0
f1 (ppm)

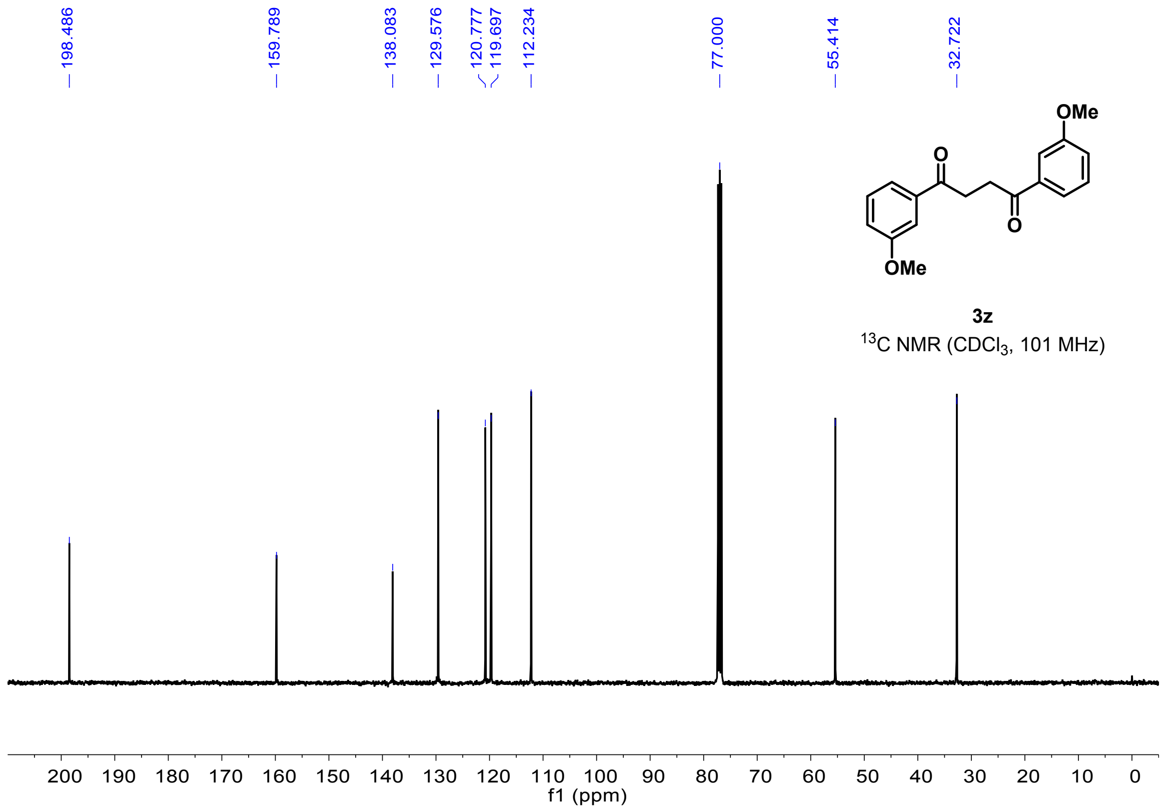
198.486
159.789
138.083
129.576
120.777
119.697
112.234
77.000
55.414
32.722
OMe
O
O
OMe
3z
13C NMR (CDCl3, 101 MHz)
200 190 180 170 160 150 140 130 120 110 100 90 80 70 60 50 40 30 20 10 0
f1 (ppm)

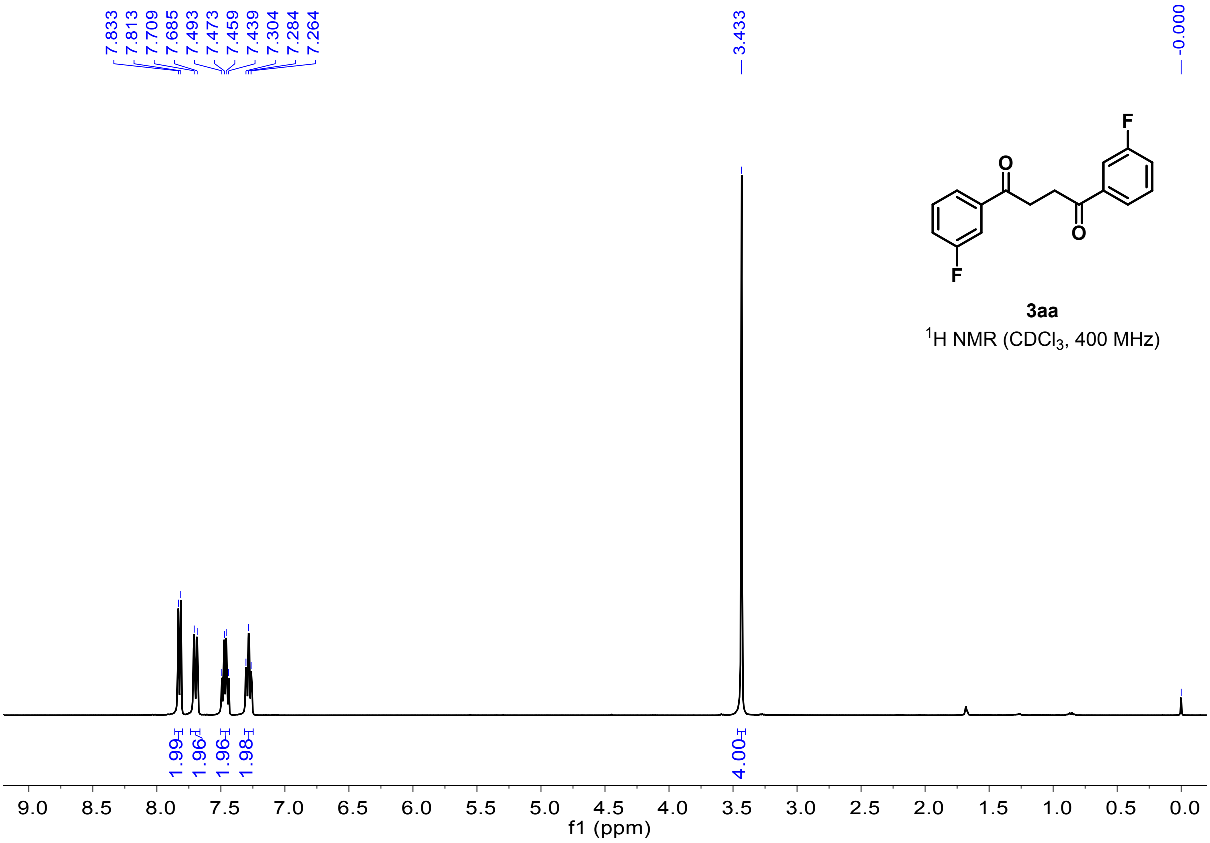


**3aa**

$^{1}H$ NMR ($CDCl_3$, 400 MHz)

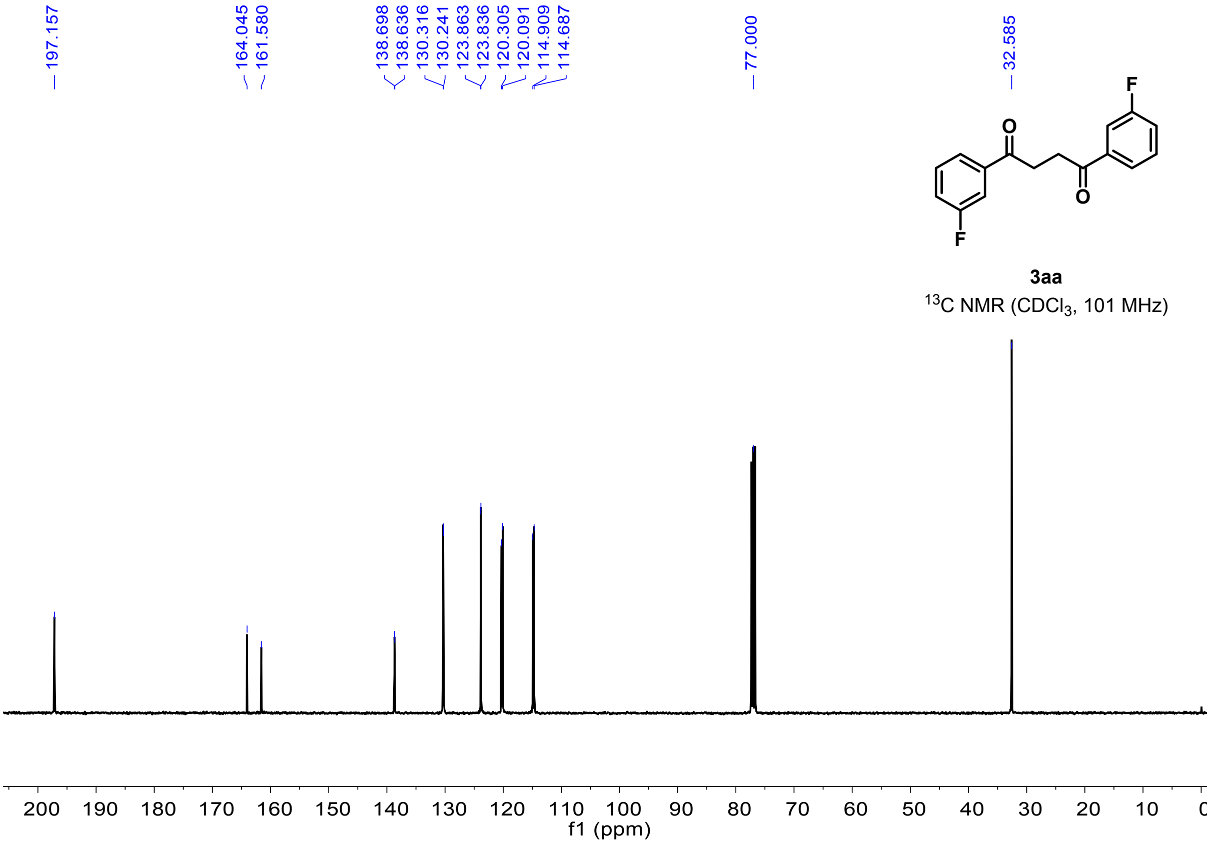


**3aa**

$^{13}C$ NMR ($CDCl_3$, 101 MHz)

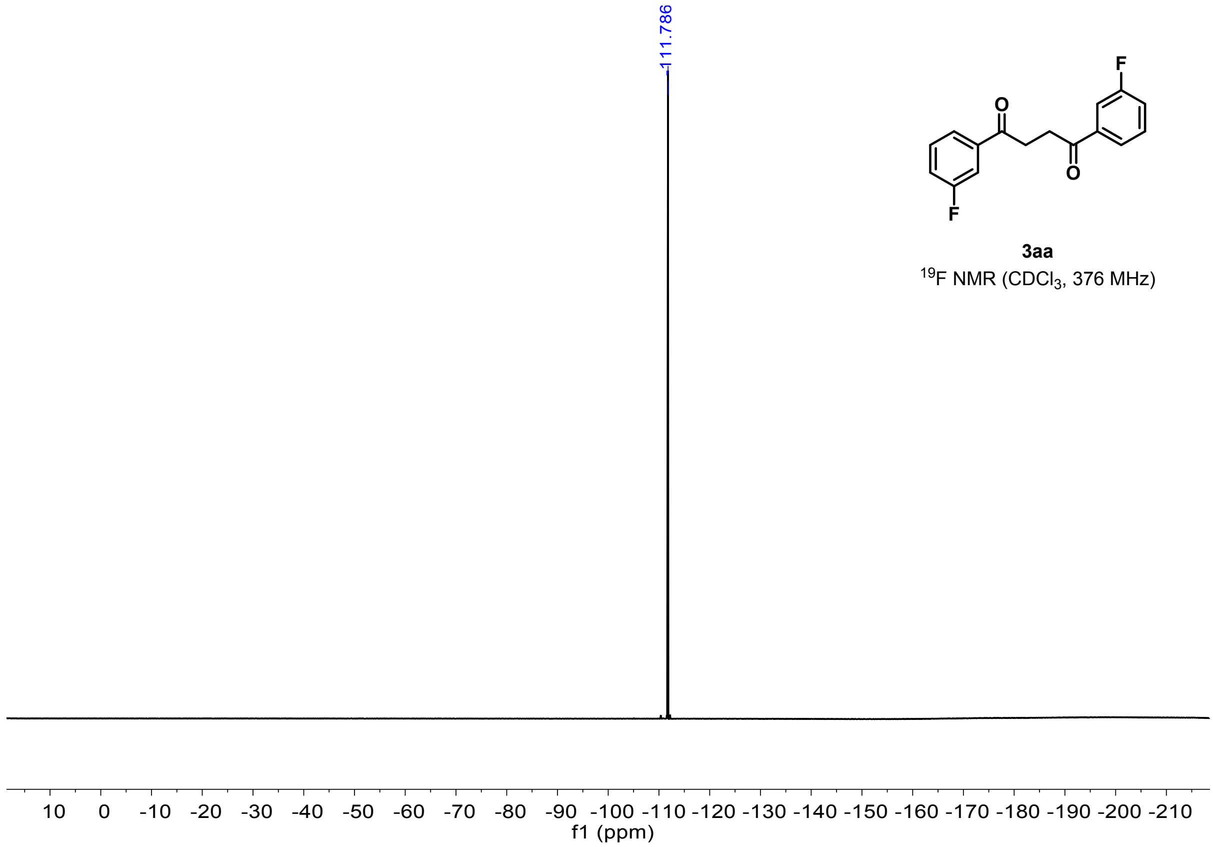

-111.786
F
O
O
F
3aa
19F NMR (CDCl3, 376 MHz)
10 0 -10 -20 -30 -40 -50 -60 -70 -80 -90 -100 -110 -120 -130 -140 -150 -160 -170 -180 -190 -200 -210
f1 (ppm)

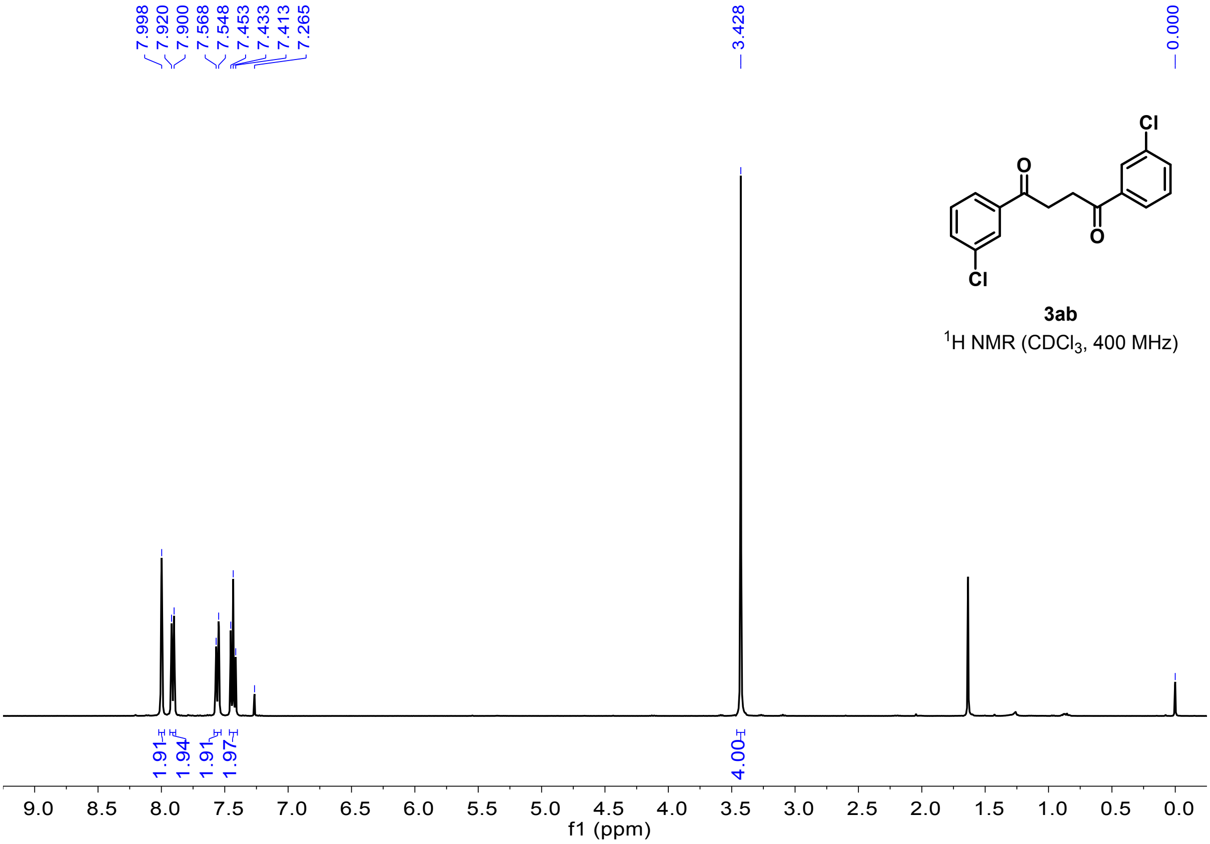


**3ab**

$^1H$ NMR ($CDCl_3$, 400 MHz)

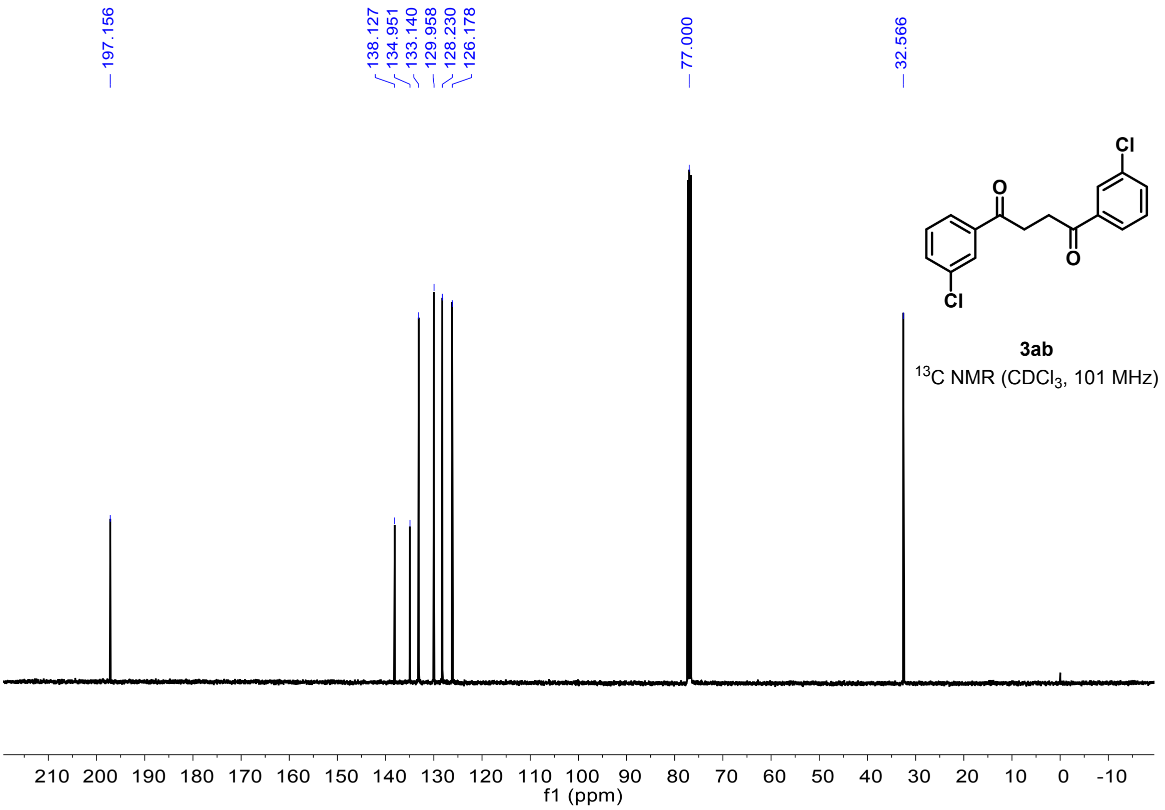


**3ab**

$^{13}C$ NMR ($CDCl_3$, 101 MHz)

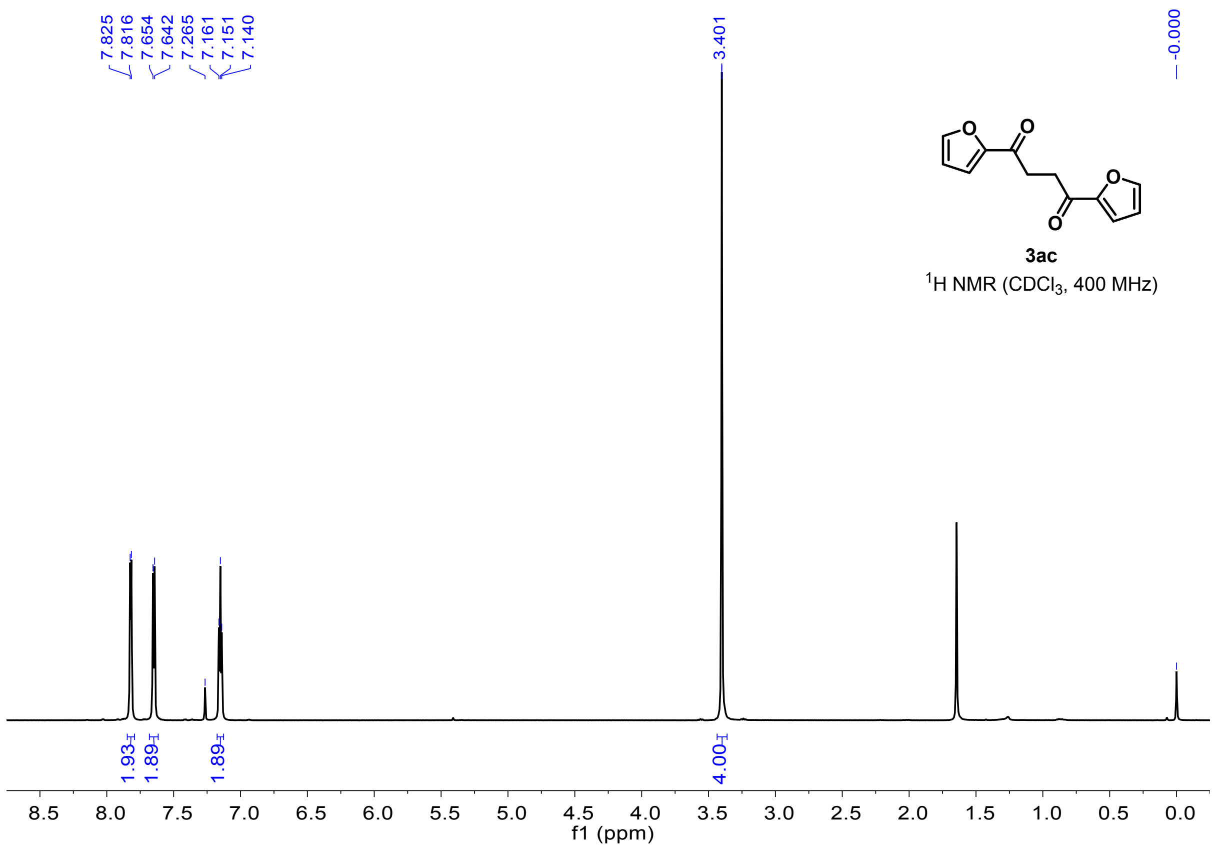

7.825
7.816
7.654
7.642
7.265
7.161
7.151
7.140
3.401
-0.000
3ac
$^1$H NMR (CDCl$_3$, 400 MHz)
1.93
1.89
1.89
4.00
f1 (ppm)


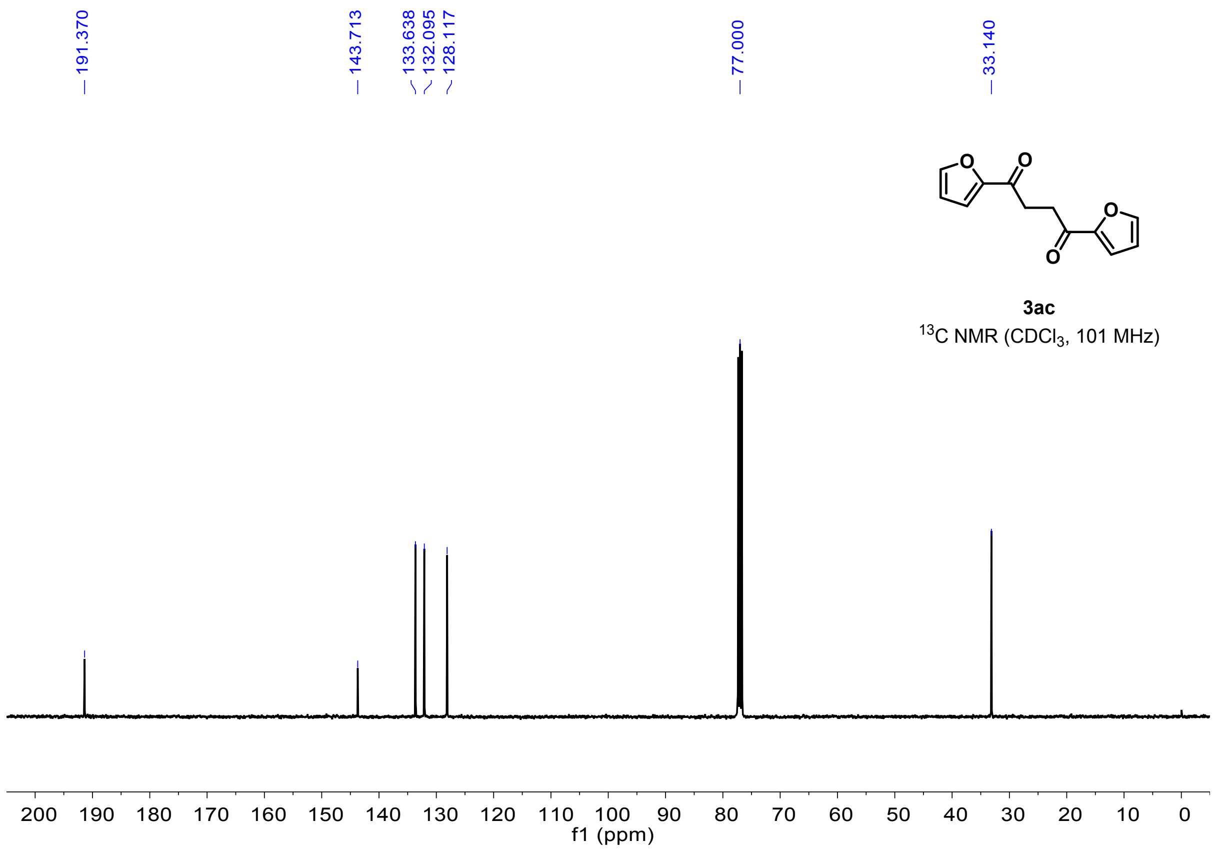

191.370
143.713
133.638
132.095
128.117
77.000
33.140
3ac
$^{13}$C NMR (CDCl$_3$, 101 MHz)
f1 (ppm)

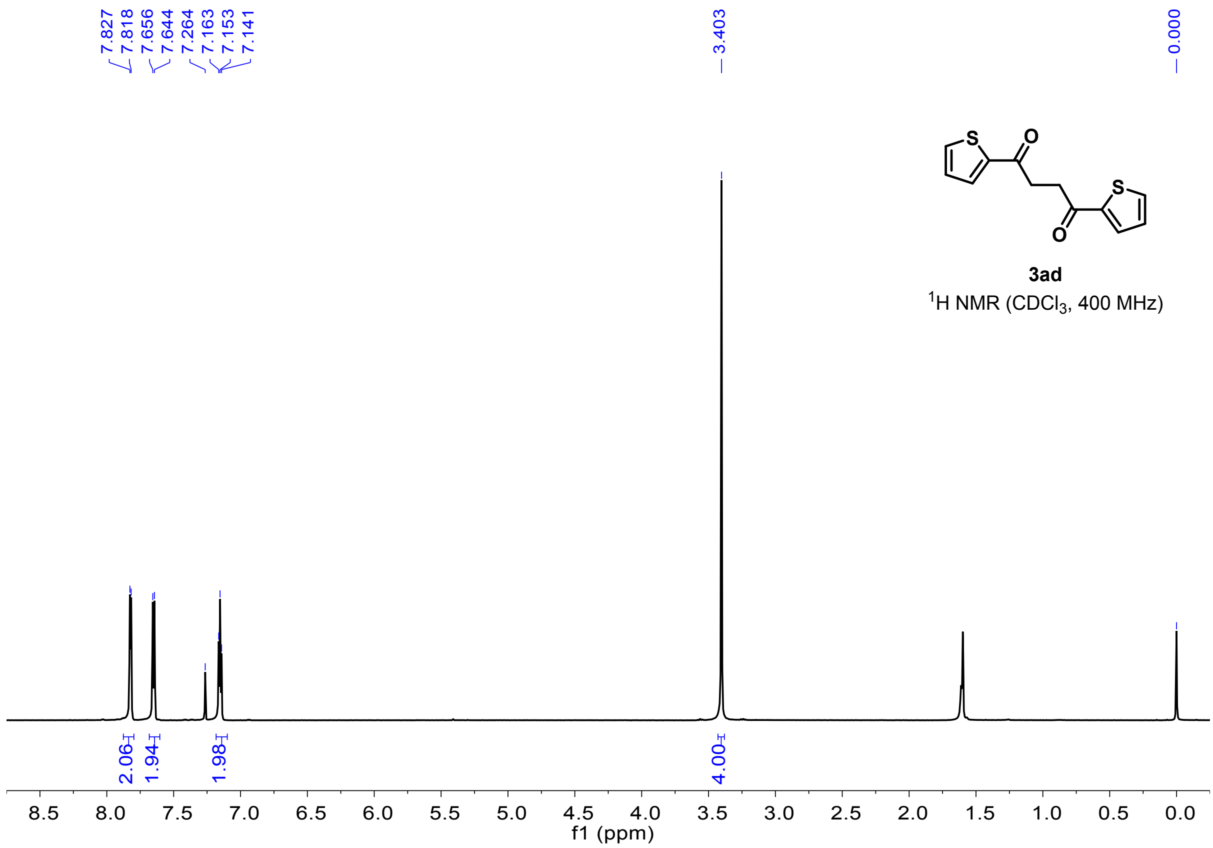
7.827
7.818
7.656
7.644
7.264
7.163
7.153
7.141
3.403
0.000
S
O
S
O
3ad
1H NMR (CDCl3, 400 MHz)
2.06
1.94
1.98
4.00
8.5 8.0 7.5 7.0 6.5 6.0 5.5 5.0 4.5 4.0 3.5 3.0 2.5 2.0 1.5 1.0 0.5 0.0
f1 (ppm)

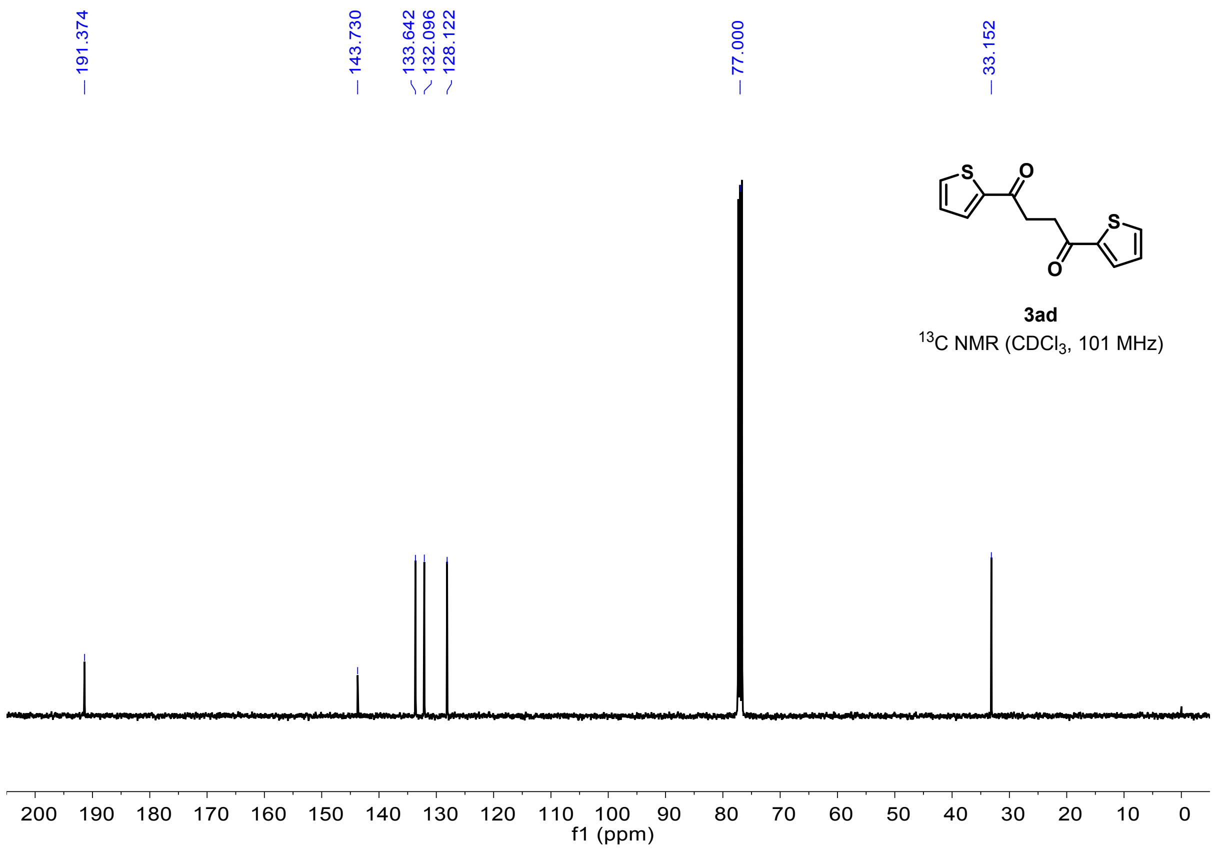
191.374
143.730
133.642
132.096
128.122
77.000
33.152
S
O
S
O
3ad
13C NMR (CDCl3, 101 MHz)
200 190 180 170 160 150 140 130 120 110 100 90 80 70 60 50 40 30 20 10 0
f1 (ppm)

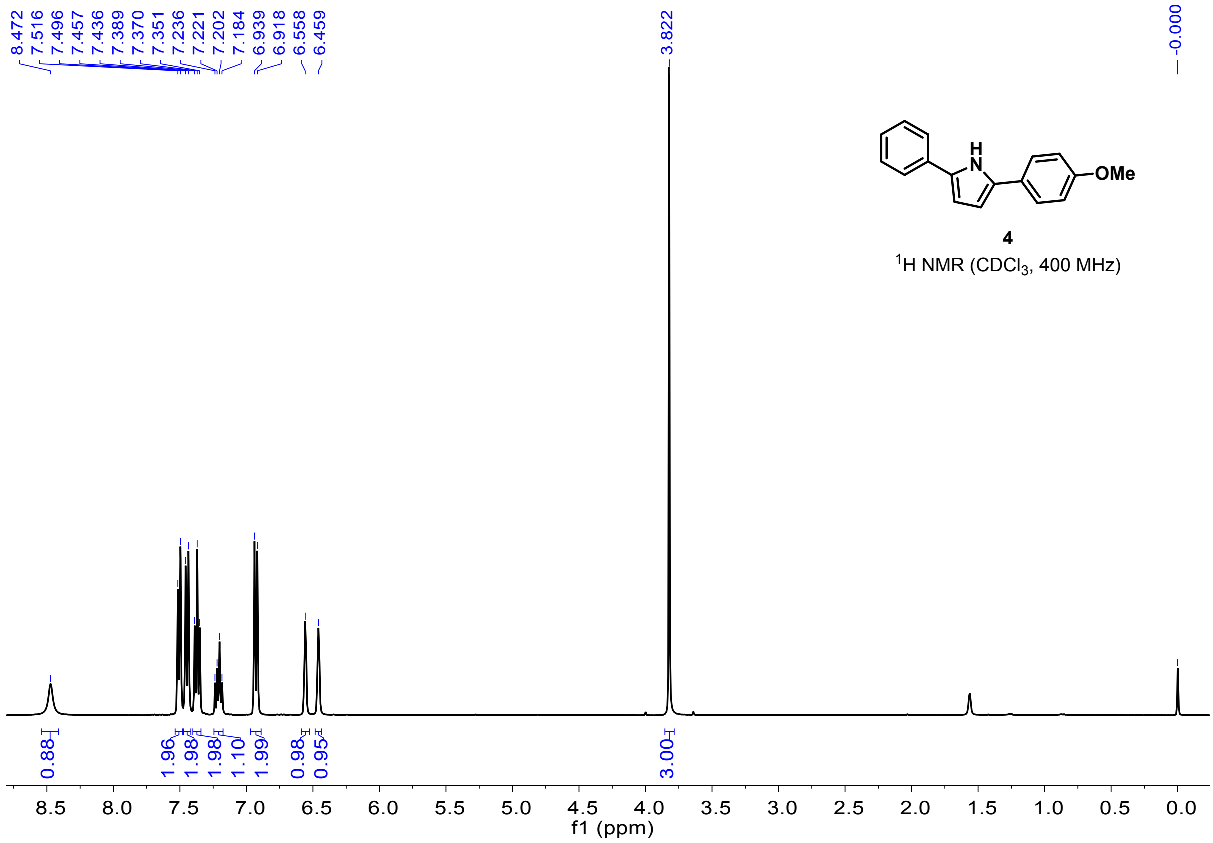
8.472
7.516
7.496
7.457
7.436
7.389
7.370
7.351
7.236
7.221
7.202
7.184
6.939
6.918
6.558
6.459
3.822
-0.000
H
N
OMe
4
$^{1}$H NMR (CDCl$_3$, 400 MHz)
0.88
1.96
1.98
1.98
1.10
1.99
0.98
0.95
3.00
8.5
8.0
7.5
7.0
6.5
6.0
5.5
5.0
4.5
4.0
3.5
3.0
2.5
2.0
1.5
1.0
0.5
0.0
f1 (ppm)

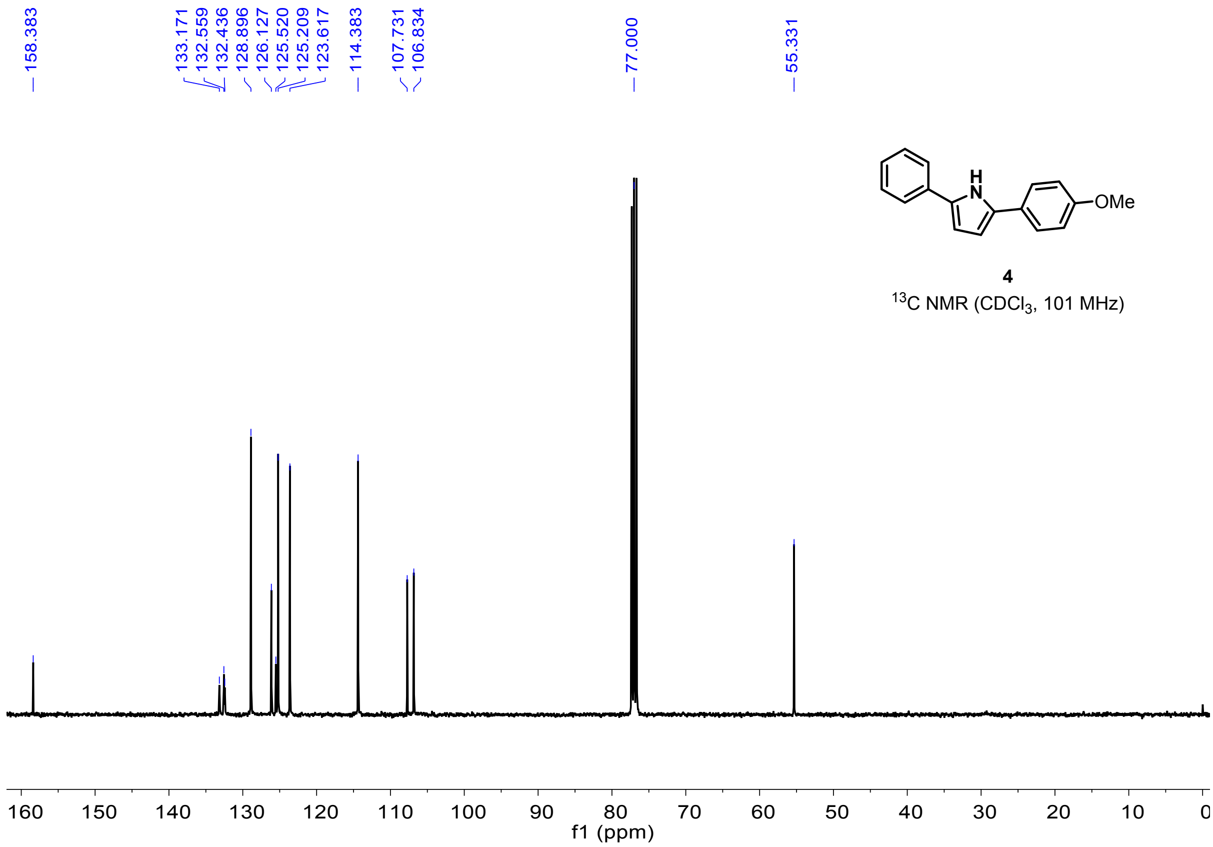
158.383
133.171
132.559
132.436
128.896
126.127
125.520
125.209
123.617
114.383
107.731
106.834
77.000
55.331
H
N
OMe
4
$^{13}$C NMR (CDCl$_3$, 101 MHz)
160
150
140
130
120
110
100
90
80
70
60
50
40
30
20
10
0
f1 (ppm)

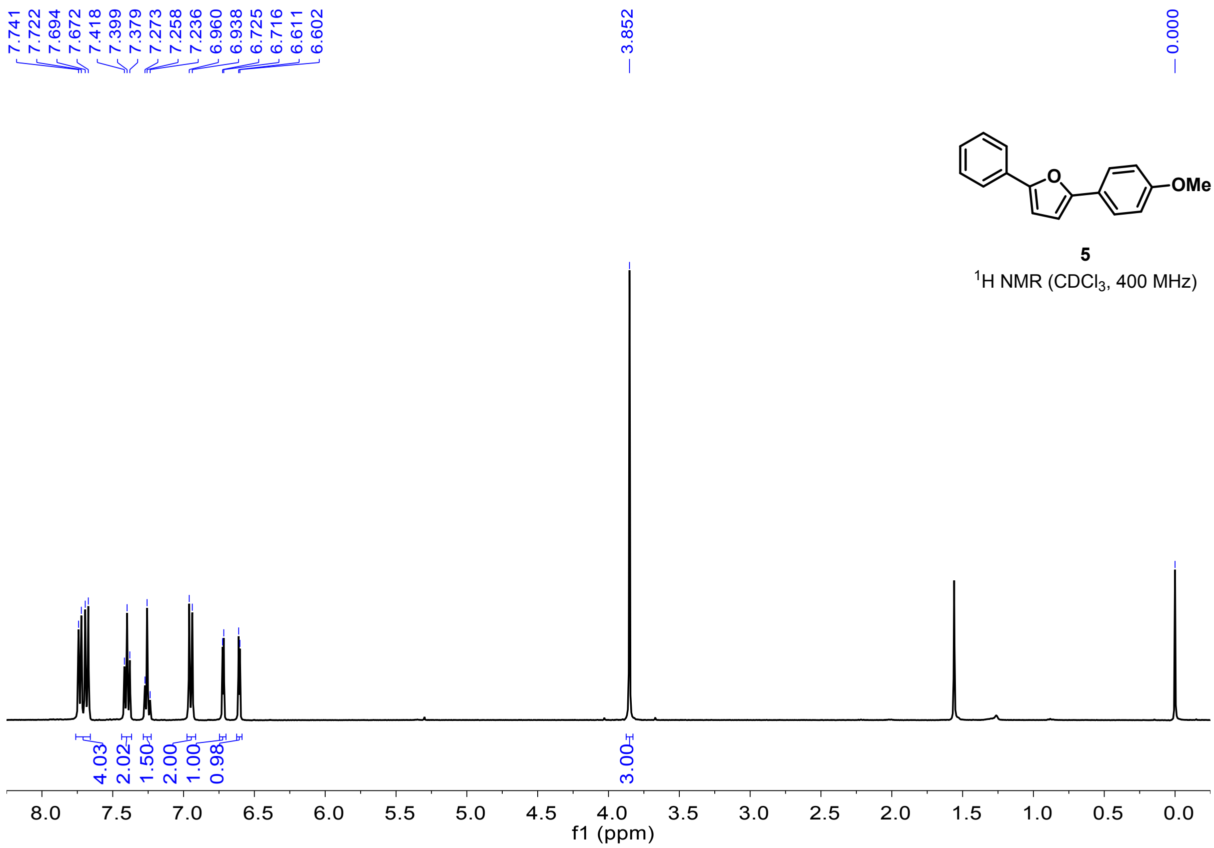
OMe
5
$^{1}$H NMR ($CDCl_3$, 400 MHz)
f1 (ppm)

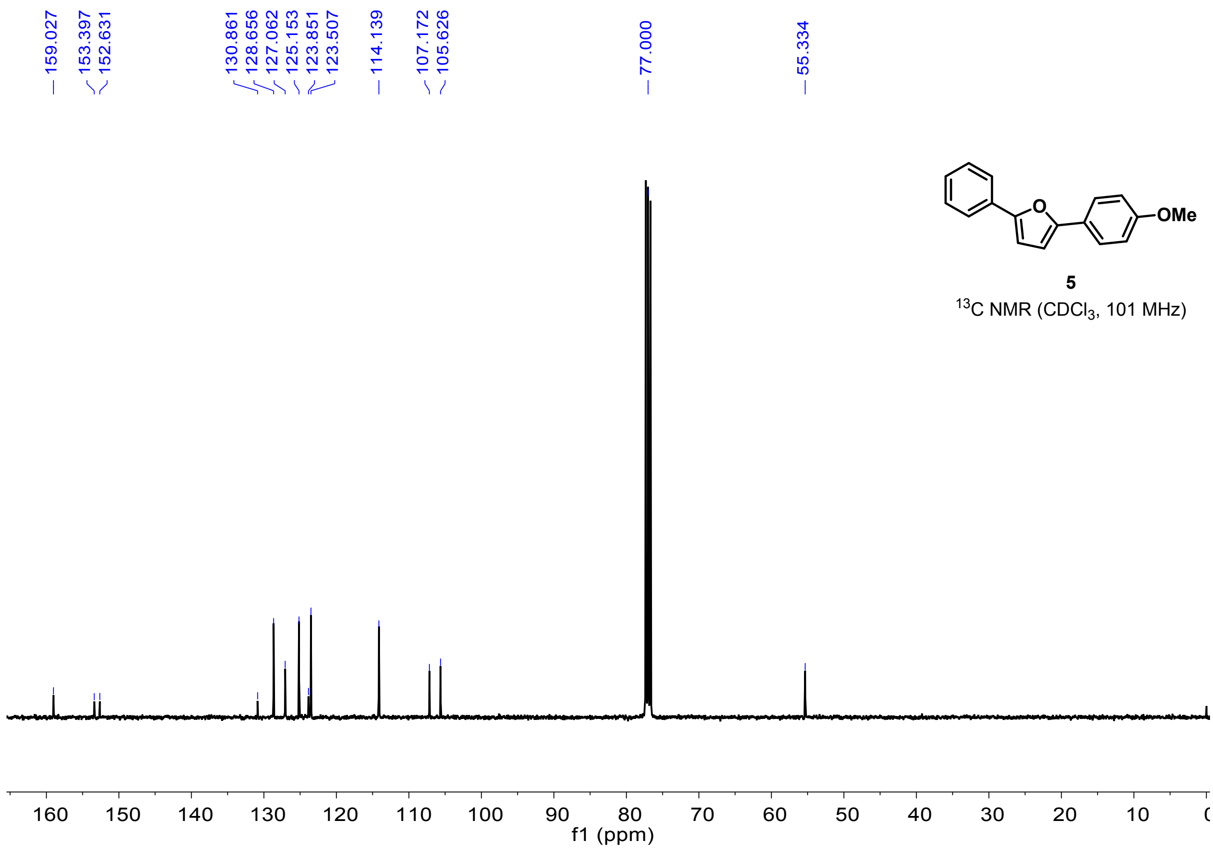
OMe
5
$^{13}$C NMR ($CDCl_3$, 101 MHz)
f1 (ppm)

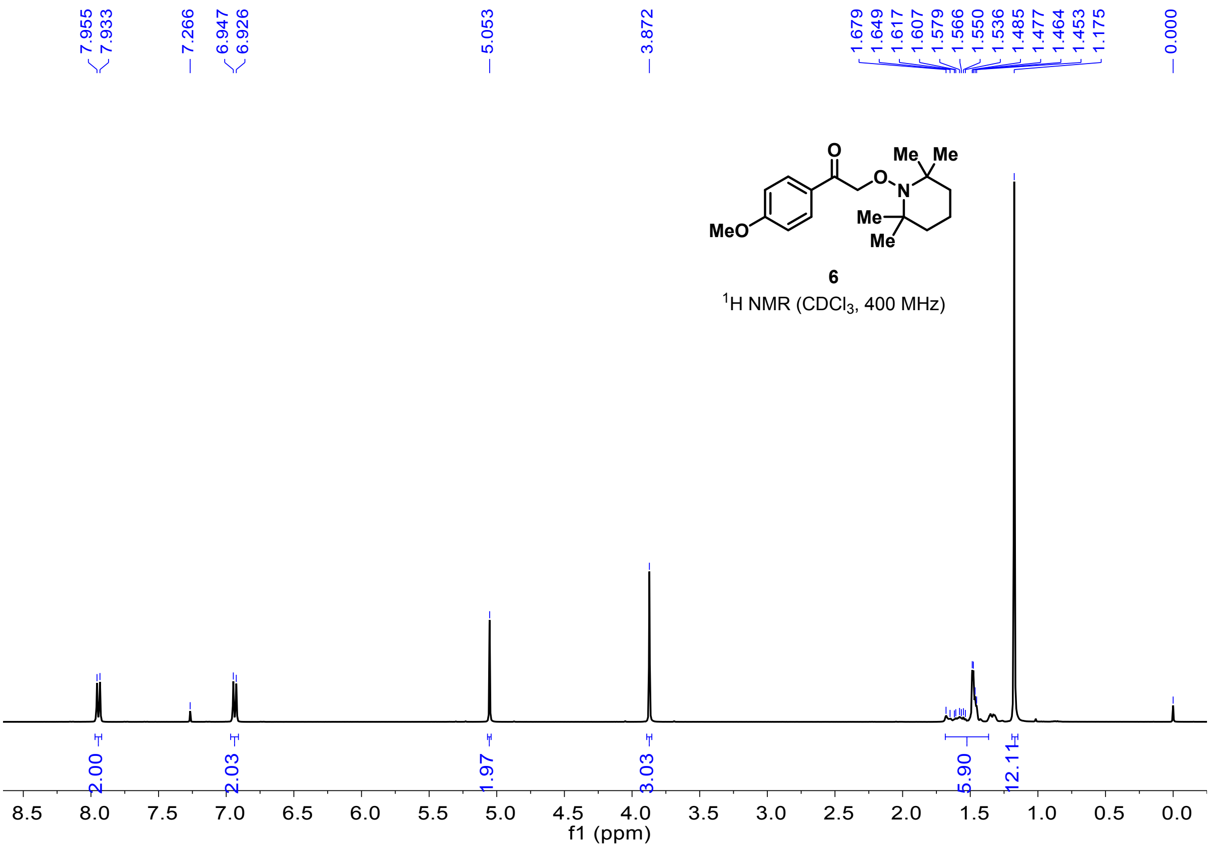
7.955
7.933
7.266
6.947
6.926
5.053
3.872
1.679
1.649
1.617
1.607
1.579
1.566
1.550
1.536
1.485
1.477
1.464
1.453
1.175
0.000
O
Me
Me
O
N
MeO
Me
Me
6
$^{1}$H NMR ($CDCl_3$, 400 MHz)
2.00
2.03
1.97
3.03
5.90
12.11
f1 (ppm)

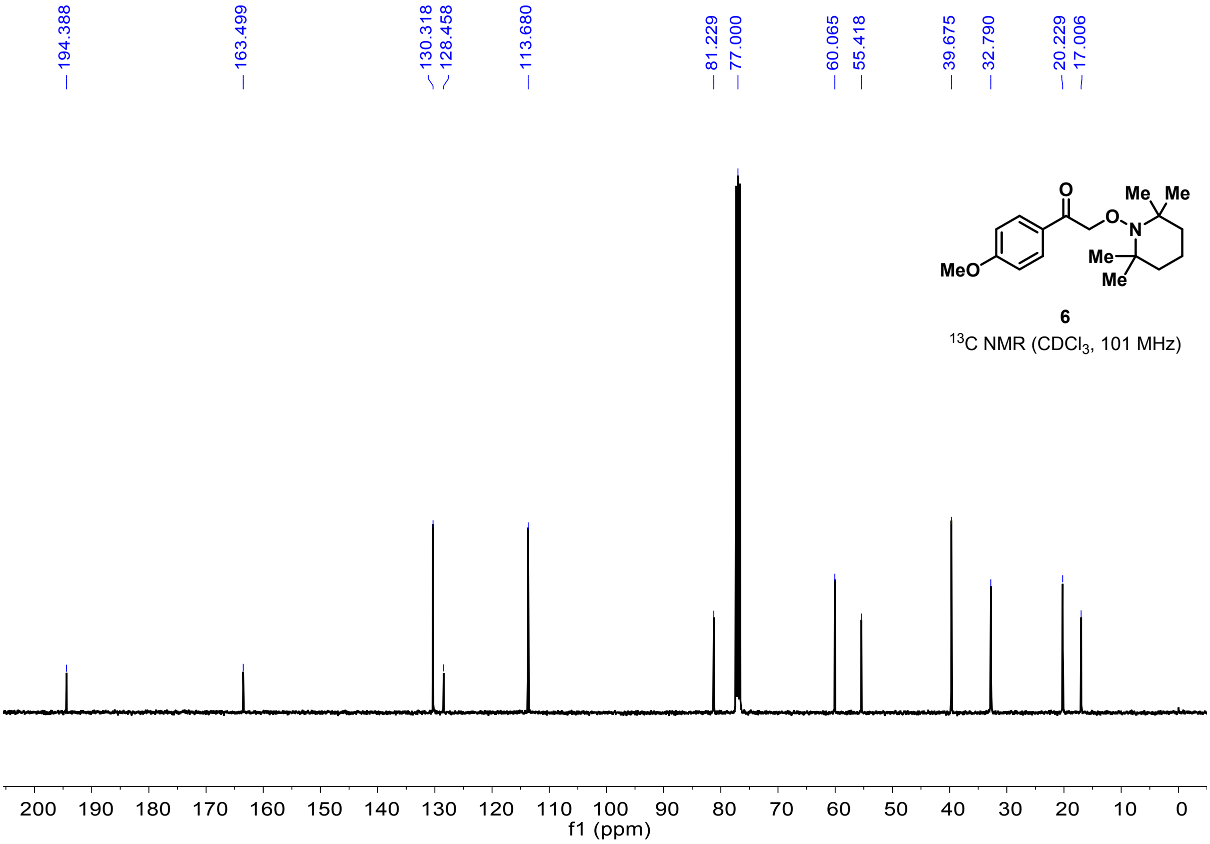
194.388
163.499
130.318
128.458
113.680
81.229
77.000
60.065
55.418
39.675
32.790
20.229
17.006
O
Me
Me
O
N
MeO
Me
Me
6
$^{13}$C NMR ($CDCl_3$, 101 MHz)
f1 (ppm)

## VI. Detection of phenyltrimethylsilane (PhTMS)

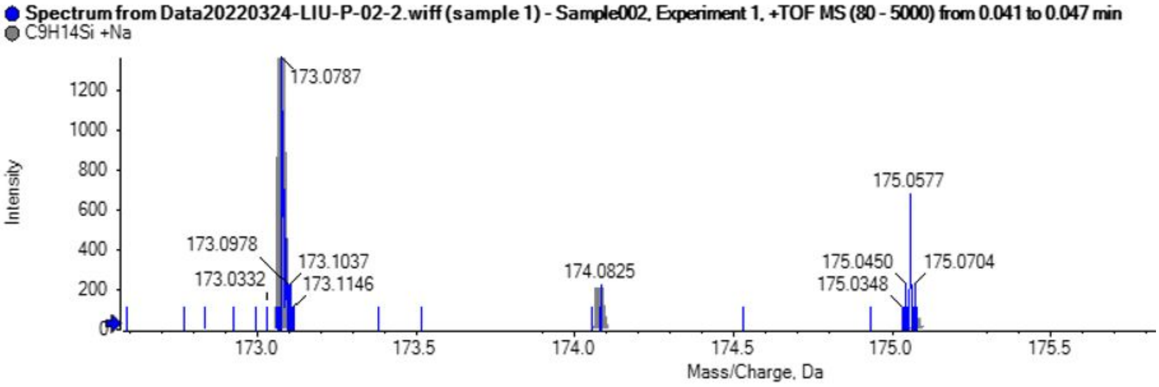


**Figure S1.** Detection of phenyltrimetylsilane (PhTMS) by ESI-MS in positive mode.

## VII. References


(1) (a) G. M. Sheldrick, SHELXS-97, Program for Solution of Crystal Structures, University of Gottingen, Germany, 1997. (b) G. M. Sheldrick, SHELXL-97, Program for Refinement of Crystal Structures, University of Gottingen, Germany, 1997.

(2) Fedorov, O. V.; Kosobokov, M. D.; Levin, V. V.; Struchkova, M. I.; Dilman, A. D. *J. Org. Chem*. **2015**, *80*, 5870−5876.

(3) Prasanna, R.; Guha, S.; Sekar, G. *Org. Lett*. **2019**, *21*, 2650-2653.

(4) Yin, H.; Nielsen, D. U.; Johansen, M. K.; Lindhardt, A. T.; Skrydstrup, T. *ACS Catal*. **2016**, *6*, 2982-2987.

(5) Si, S.; Wang, C.; Zhang, N.; Zou, G. *J. Org. Chem.* **2016**, *81*, 4364-4370.

(6) Esumi, N.; Suzuki, K.; Nishimoto, Y.; Yasuda, M. *Org. Lett.* **2016**, *18*, 5704-5707.

(7) Li, Y.; Shang, J.-Q.; Wang, X.-X.; Xia, W.-J.; Yang, T.; Xin, Y.; Li, Y.-M. *Org. Lett.* **2019**, *21*, 2227-2230.

(8) Peppe, C.; Pavão das Chagas, R. *Synlett.* **2004**, *7*, 1187-1190.

(9) Shen, Z.-L.; Goh, K. K. K.; Cheong, H.-L.; Wong, C. H. A.; Lai, Y.-C.; Yang, Y.-S.; Loh, T.-P. *J. Am. Chem. Soc*. **2010**, *132*, 15852-15855.

(10) Nevar, N. M.; Kel'in, A. V.; Kulinkovich, O. G. *Synthesis.* **2000**, *9*, 1259-1262.

(11) Ceylan, M.; Gürdere, M. B.; Budak, Y.; Kazaz, C.; Seçen, H. *Synthesis.* **2004**, *11*, 1750-1754.

(12) Xuan, J.; Feng, Z.-J.; Chen, J.-R.; Lu, L.-Q.; Xiao, W.-J. *Chem. Eur. J.* **2014**, *20*, 3045-3049.

(13) Cen, J.; Wu, Y.; Li, J.; Huang, L.; Wu, W.; Zhu, Z.; Yang, S.; Jiang, H. *Org. Lett*. **2019**, *21*, 2090-2094.

(14) Jiang, H.; Zeng, W.; Li, Y.; Wu, W.; Huang, L.; Fu, W. *J. Org. Chem*. **2012**, *77*, 5179-5183.

(15) Zawodny, W.; Teskey, C. J.; Mishevska, M.; Völkl, M.; Maryasin, B.; González, L.; Maulide, N., *Angew. Chem., Int. Ed.* **2020**, *59*, 20935-20939.
